# Recent Advances in Hole-Transporting Layers for Organic Solar Cells

**DOI:** 10.3390/nano12030443

**Published:** 2022-01-28

**Authors:** Cinthya Anrango-Camacho, Karla Pavón-Ipiales, Bernardo A. Frontana-Uribe, Alex Palma-Cando

**Affiliations:** 1 Grupo de Investigación Aplicada en Materiales y Procesos (GIAMP), School of Chemical Sciences and Engineering, Yachay Tech University, Hda. San José s/n y Proyecto Yachay, Urcuqui 100119, Ecuador; cinthya.anrango@yachaytech.edu.ec (C.A.-C.); karla.pavon@yachaytech.edu.ec (K.P.-I.); 2 Centro Conjunto de Investigación en Química Sustentable UAEMex-UNAM, Carretera Toluca Atlacomulco, Km 14.5, Toluca 50200, Mexico; bafrontu@unam.mx; 3 Instituto de Química, Universidad Nacional Autónoma de México, Circuito Exterior, Ciudad Universitaria, Ciudad de México 04510, Mexico

**Keywords:** hole transporting layer, organic solar cells, photoconversion efficiency, stability, metal oxides, metal sulfides, nanocarbon materials, conducting polymers, conjugated polyelectrolyte, small organic molecules

## Abstract

Global energy demand is increasing; thus, emerging renewable energy sources, such as organic solar cells (OSCs), are fundamental to mitigate the negative effects of fuel consumption. Within OSC’s advancements, the development of efficient and stable interface materials is essential to achieve high performance, long-term stability, low costs, and broader applicability. Inorganic and nanocarbon-based materials show a suitable work function, tunable optical/electronic properties, stability to the presence of moisture, and facile solution processing, while organic conducting polymers and small molecules have some advantages such as fast and low-cost production, solution process, low energy payback time, light weight, and less adverse environmental impact, making them attractive as hole transporting layers (HTLs) for OSCs. This review looked at the recent progress in metal oxides, metal sulfides, nanocarbon materials, conducting polymers, and small organic molecules as HTLs in OSCs over the past five years. The endeavors in research and technology have optimized the preparation and deposition methods of HTLs. Strategies of doping, composite/hybrid formation, and modifications have also tuned the optical/electrical properties of these materials as HTLs to obtain efficient and stable OSCs. We highlighted the impact of structure, composition, and processing conditions of inorganic and organic materials as HTLs in conventional and inverted OSCs.

## 1. Introduction

Solar energy has enough power capacity to satisfy the whole world’s demand [1,2]. According to Luqman et al. [3], the amount of solar energy irradiated at the Earth’s atmosphere ranges from 200 to 250 Wm^−2^ per day, of which ca. 70% is available for conversion into power generation [4,5]. Research on solar energy technology, which aims to convert sunlight directly into electrical energy, is vital to switch into low-carbon energy systems [6,7]. The intense developments concerning solar energy have boosted the investigations to optimize the efficiency and stability of emerging photovoltaic technology, such as dye-sensitized solar cells, organic solar cells (OSCs), perovskite solar cells, quantum dot solar cells, and so on, of which OSCs are one of the most promising technologies [8,9,10,11,12,13,14]. Since Kearns and Calvin’s pioneering work on OSCs in 1958, one significant breakthrough in solar energy technology has been the efficient electron transfer between a conjugated polymer and fullerene derivative [15,16]. It encouraged the interest in the light-harvesting of OSCs from the structure device into the materials used for their construction [16,17,18,19]. OSCs are based on organic semiconductors as active layers with unique advantages to achieve low-cost renewable energy harvesting, owing to their material and manufacturing advances [20,21]. OSCs have some advantages: low-cost fabrication, solution processes, light weight, flexibility, a great opportunity for large-scale roll-to-roll production, and a low environmental disposable impact. Furthermore, OSCs showed shortened energy payback time, that is, the time needed to recover the device fabrication energy [22]. OSCs’ record efficiency is over 18.2% in single cells [23,24] and over 18.6% in tandem cells [25]. In the last ten years, extensive research and development have been conducted in OSCs to improve lifetimes (7–10 years) and the power conversion efficiency (PCE) over 10% in roll-to-roll industrial manufacturing [23,26,27,28]. Some companies have developed OSCs commercially, such as Heliatek, infinityPV, and OPVIUS GmbH, which manufacture flexible OSC modules [29] representing 5% of the market [30]. OSCs have been used in small-scale applications as building integrated photovoltaics, e.g., incorporated on roofs and walls of storage buildings and solar parks [31].

The core of the OSCs is a blend of electron-donor materials (e.g., conjugated polymers) and fullerene-based or non-fullerene-based electron-acceptor materials [32]. This central layer is called the photoactive layer and absorbs solar radiation. A typical OSC has a bulk heterojunction (BHJ) structure that is a mixed-blend of donor and acceptor materials, which constitute the photoactive layer [33]. When the solar cell is irradiated, the photoactive layer absorbs photons to generate excitons (bound electron-hole pairs), which dissociate into free charge carriers in the donor-acceptor interface, producing separated holes and electrons. These free charges are then extracted and transported to the corresponding electrodes [34,35]. Interfacial layers are generally utilized to tailor the work function (WF) of electrodes for the maximization of charge carrier (e.g., electrons and holes) collection. They modify the interface to alter the photoactive layer morphology and minimize charge carrier recombination (improving the charge selectivity) at the interface between the active layer and transport layer [36]. Moreover, the interfacial layers help to form an ohmic contact between electrodes and active layers as well as tune the energy level alignment to facilitate the charge extraction [37,38]. Hole-transporting layers (HTLs), also called anode interfacial layers (AILs), facilitate hole extraction and transportation while blocking electron flux. Hole-transport materials are deposited between the photoactive layer and the anode, improving the device performance. HTLs, used in conventional polymer solar cells (PSCs), were first reported in the late 1990s after a similarly reported experimentation in organic light-emitting diodes (OLEDs) [39,40]. Some important characteristics are required for hole-transport materials such as a high conductivity, high transparency (since the sunlight is absorbed by the photoactive layer through the HTL on anode), solution processability and favorable stability, high WF (since the energy level of materials should be appropriate for charge collection), and predominantly good hole mobility [39].

Over the past five years, the research community has been working on achieving high efficiency and stability and low cost of production on emerging clean energy sources, such as OSCs, with a priority on interfacial layer engineering. Tian et al. analyzed the diverse molecular structures employed as HTL and electron transporting layers (ETLs) to minimize energy losses in non-fullerene OSCs [41]. Palilis et al. discussed the relationship between the optoelectronic and physical properties of inorganic materials and their functionality at the interface [42]. Gusain et al. showed the physical mechanisms involved with the interfacial issues and the routes adopted to address them [43]. Amollo et al. explored the physical and optical properties of polymers and metal oxides together with their hybrids and graphene to guide the choice of suitable interfacial materials [44]. Wu et al. showed the impact of nanotechnology and nanomaterials in manufacturing multifunctional interfacial layers to enhance OSCs’ performance [45]. Huang et al. reviewed the feasibility of tuning the optical and electrical properties of solution-processed ternary oxides, as potential carrier transports layers, from the large range of crystal structures and adjustable atomic ratio [46]. Herein, we presented an extensive state-of-the-art review about the advances in HTLs that show great potential for enhancing the efficiency (e.g., PCE) and stability of OSCs. The progress made on improving HTL properties of inorganic (metal oxides and sulfides), nanocarbon materials, conjugated polymers, and small organic molecules as HTLs in OSCs was discussed, focusing on solution-processing conditions, deposition methods, doping, composite/hybrid formation, and chemical modifications. Considering the numerous and highly dispersed literature, we tried to include relevant information reported in scientific journals. This review included a short section on the structure and characterization of OSCs and some remarks on HTLs followed by reports of the last five years in the use of hole-transporting materials as HTLs in OSCs. Summary tables of the photovoltaic device architecture and their performance are presented at the end of Section 4.3 and Section 4.5.

## 2. Structure and Characterization of Organic Solar Cells

**Figure 1:**
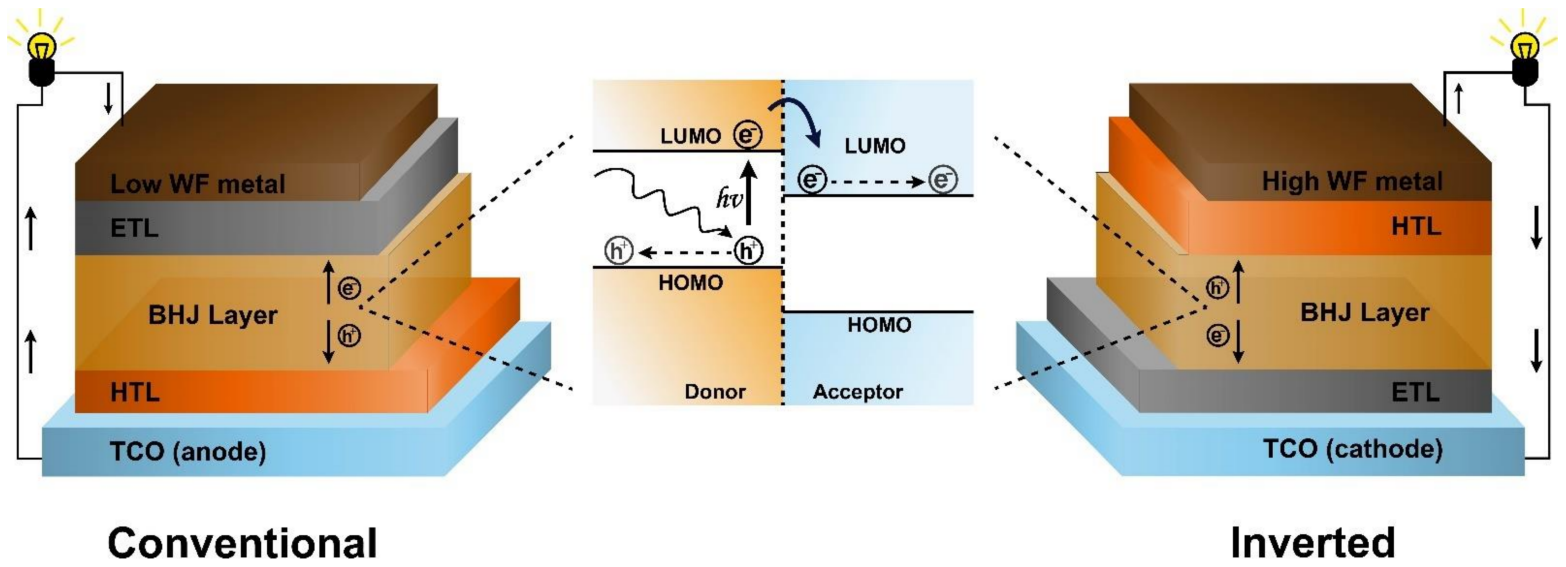
Schematic structure of conventional and inverted OSCs, and a simplified view of the operating principle in the active layer.

A conventional OSC consists of an active layer sandwiched between two electrodes with their respective extracting layers to ensure mobility, collection, and transport of the charge carriers [47]. At the bottom, the anode electrode is a transparent conductive oxide, such as indium tin oxide (ITO), and at the top, the cathode is a low WF metal, such as Ca and Al (see Figure 1) [48]. The OSCs based on two organic semiconductors in the active layer can have two architectures: the bilayer and the BHJ devices. Tang et al. presented the sequential stacking of donor and acceptor semiconductors to form the bilayer planar heterojunction in 1986 [17]. However, it has limitations, such as the small surface area between the donor/acceptor interface and the poor excitons’ dissociation. Then, the introduction of BHJ devices in 1990 solved bilayer devices’ issues [19]. They involve mixing donor and acceptor materials in the bulk body of an OSC to reduce phase separation. Donor and acceptor domains are twice the size of the exciton diffusion length (~10 nm). To expand the active layer’s absorption range, tandem OSCs have been proposed to stack two single-junctions with different absorption ranges [49,50]. According to the charge flow direction, OSCs can be divided into conventional and inverted devices (see Figure 1) [51]. Under light irradiation, photons are absorbed by the donor material in the active layer to form excited states, called excitons, which are bound electron-hole pairs. Excitons diffuse towards the donor/acceptor material interface and separate into free charge carriers. Holes and electrons move apart in the highest occupied molecular orbital (HOMO) and the lowest unoccupied molecular orbital (LUMO) levels, respectively (see Figure 1). Then, the separated charge carriers are transported and collected at the electrodes supplying a photocurrent [52].

**Figure 2:**
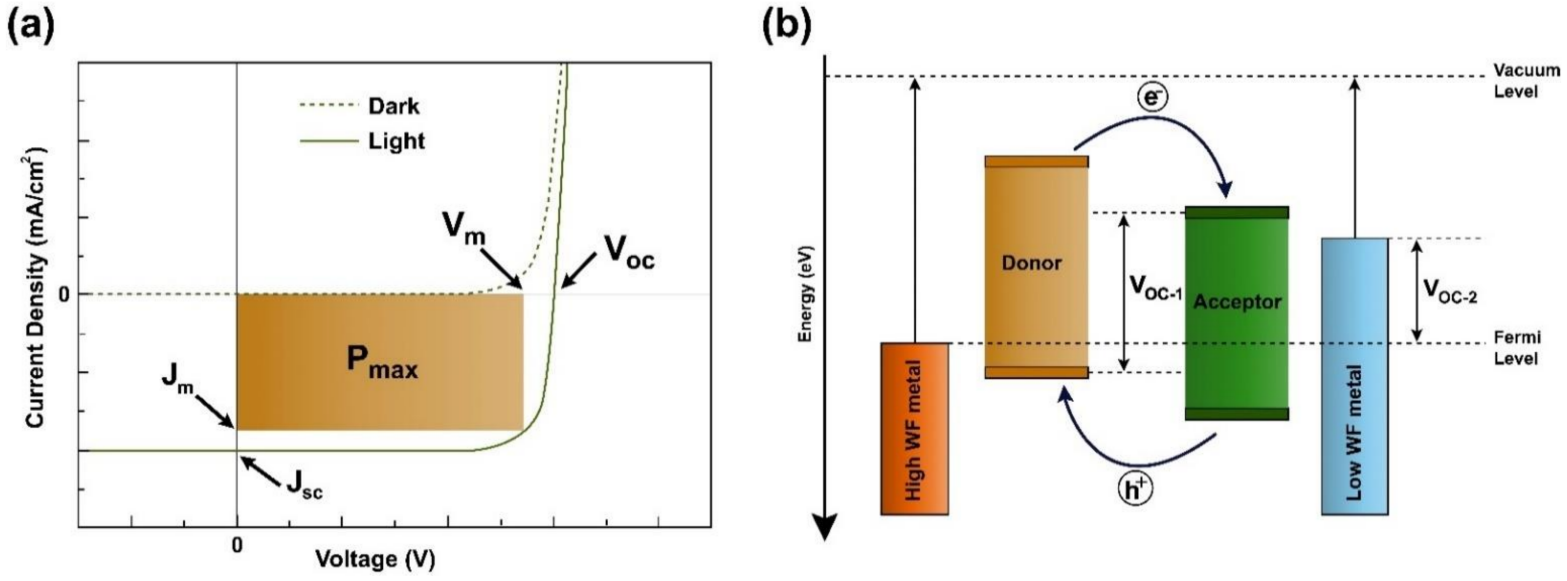
(**a**) Typical J-V curve and (**b**) schematic representation of the V_OC_ in a conventional OSC.

The current-voltage (J-V) curve of an OSC is characterized under 1000 W/m^2^ light of AM 1.5 solar spectrum [53]. Figure 2a shows a J-V curve of an OSC under darkness (dashed line) and illumination (solid line) conditions. Photocurrent is not flowing through the electrodes under dark conditions, just the current by the forward bias of contacts as a diode. Under irradiation, photocurrent is generated. PCE is determined by the product of three parameters: short-circuit current density ($J_{\mathrm{sc}}$), open-circuit voltage ($V_{\mathrm{oc}}$), and fill factor (FF) over the incident light power density ($P_{\mathrm{in}}$) as follows [54]:

(1) $$\mathrm{PCE}=\frac{V_{\mathrm{oc}}\times J_{\mathrm{sc}}\times \mathrm{FF}}{P_{\mathrm{in}}}$$

The ratio of collected photogenerated charges and the number of incident photons is related to the external quantum efficiency (EQE) of the OSC. $V_{\mathrm{oc}}$ is the main driving force for charge separation once the exciton reaches the donor/acceptor interface [55,56]. $V_{\mathrm{oc}}$ is the difference of WFs between the quasi-Fermi levels of holes (E_F.h_) in the HOMO level of the donor and the quasi-Fermi levels of electrons (E_F,e_) in the LUMO level of the acceptor in a BHJ under the formation of ohmic contacts with the cathode and anode (depicted as $V_{\mathrm{oc}-1}$ in Figure 2b). If a Schottky contact appears in both BHJ/electrode interfaces, the $V_{\mathrm{oc}}$ would decrease and would depend on the difference between the WFs of the two metal contacts (depicted as $V_{\mathrm{oc}-2}$ in Figure 2b) [57,58]. FF is the ratio between the maximum power output ($P_{\max}$) and the maximum attainable power output ($J_{\mathrm{sc}}{\;V}_{\mathrm{oc}}$). $P_{\max}$ describes the maximum power drawn from the device and is the product of the maximum current ($J_{m}$) and voltage ($V_{m}$) (see Figure 2a) [59], as follows:

(2) $$\mathrm{FF}=\frac{P_{\max}}{J_{\mathrm{sc}}{\;V}_{\mathrm{oc}}}=\frac{J_{m}{\;V}_{m}}{J_{\mathrm{sc}}{\;V}_{\mathrm{oc}}}$$

The main factors that influence the FF are the series resistance ($R_{s}$) and the shunt resistance ($R_{\mathrm{sh}}$). Their interaction determines the current flow. $R_{s}$ is attributed to the conductivity of electrodes, BHJ and extracting interface layers, as well as the contact resistance between them [60]. A small $R_{s}$ increases the mobility of the charge carriers and the performance of OSCs. $R_{\mathrm{sh}}$ reflects the current losses from the pinholes and traps in the film. Established relationships describing J-V behavior in OSCs and directly accounting for resistance effects on cell performance are the following [61,62,63,64]:

(3) $$J=J_{d}+J_{\mathrm{sh}}-J_{\mathrm{ph}}=J_{0}\left\{\exp\left[\frac{e\left(V-{\mathrm{JR}}_{s}\right)}{{\mathrm{nk}}_{B}T}-1\right]\right\}+\frac{V\;–{\;J\;R}_{s}}{R_{\mathrm{sh}}}-J_{\mathrm{ph}}$$

where, $J_{d}$ is the diode current density, $J_{\mathrm{sh}}$ is the leakage current density, $J_{\mathrm{ph}}$ is the photogenerated current density, $J_{0}$ is the reverse saturation current, e is the elementary charge, n is the diode ideality factor, k_B_ is Boltzmann’s constant, and T is temperature. $J_{\mathrm{sh}}$ is an undesirable current injected from the electrodes in the opposite direction to $J_{\mathrm{sc}}$. A suitable interface morphology decreases $J_{\mathrm{sh}}$ and increases $R_{\mathrm{sh}}$ independently of the light intensities [65]. Thus, the contact quality at the active layer/electrode interface is critical to optimize FF, $V_{\mathrm{oc}}$, and $J_{\mathrm{sc}}$. Interface transporting layers enhance all these parameters because they tune the energy level alignment at the active layer and electrodes, the surface morphology, and the contact to boost the efficiency and stability of the OSCs [66].

## 3. Hole-Transporting Layers

Interfacial layers are critical components of OSCs to enhance the collection efficiency of holes and electrons toward the anode and cathode electrodes. In photovoltaic devices, including OSCs, there are barriers to charge extraction by the non-ideal contact between the active layer and the electrodes [39]. This limited interfacial energy alignment inhibits the spontaneous charge transport, resulting in charge accumulation at the interface, thus decreasing Voc, FF, and PCE [67]. Interfacial layers with suitable WFs contribute to match the energy levels of donor and acceptor materials with the electrodes, favoring the charge transport and stability [38]. The interfacial layers must be charged selectively to avoid charge recombination at the electrodes in addition to the tuning of the energy levels. HTLs and ETLs increase the hole and electron mobility in the opposite direction to collect only one type of charge on each electrode [68]. In the 1990s, HTLs were introduced to the organic electronics field by Tokito et al., who showed that hole-injection increased from inserting vanadium, molybdenum, and ruthenium oxides layers into OLEDs [69]. HTL’s central role is the efficient hole extraction and transport from the HTL/active interface to the anode/HTL interface, increasing power generation [70]. To achieve high-performance OSCs, the materials used for HTLs need to show (i) high WF that matches with the HOMO energy level of the donor material and the anode energy level, (ii) transparency to increase the light absorption by the active layer, (iii) high hole mobility to lower the charge accumulation and recombination, (iv) a large band gap to block electron carriers, and (v) chemical resistance to external factors [38,71,72]. The first materials used as HTLs in OSCs were inorganic p-type transition metal oxides (MoO_3_, WO_3_, NiO, Fe_3_O_4_) or metal sulfides (MoS_2_), which showed high stability and performance [73,74,75,76,77]. Most of them required high vacuum for deposition, which, compared with organic materials, might be costly for industrial and large-scale processing [78]. Poly(3,4-ethylenedioxythiophene)-poly(styrene sulfonate) (PEDOT:PSS) is still the standard conducting polymer used as HTL in OSCs because of the low costs, minimal toxicity, facile solution processing, and high WF. However, it is not stable at standard conditions owing to its hygroscopic and acidic nature [79]. Currently, there is an excellent development of cost-effective low-temperature deposition strategies for industrial scaling to avoid the traditional vacuum method used in the manufacture of HTLs. Casting process deposits the material dissolved in liquid form in a solvent on the underlying substrate, followed by drying. Spray casting solves the lack of control in film morphology and uniformity [80]. Spin coating is the most common deposition method of PEDOT:PSS due to its high reproducibility in film thickness and morphology. It applies the spinning at a certain rotation speed of the substrate to dry the deposited liquid material. However, neither large area applicability nor film patterning are achievable by this technique [81]. Electrochemical deposition or electrodeposition allows depositing polymers and inorganic materials through an electric field [82]. The control on deposition has broader applicability for the formation of composites [83]. The roll-to-roll technique is usually utilized in flexible OSCs because the flexible substrate is unwound to pass through printing or coating machines, followed by being rewound on a roll. It opens the applicability for large-area production because substrates are not handled individually but instead in rolls [84,85]. Compared with the vacuum method, these deposition techniques offer the advantage of a continuous and large-area process at mild conditions, avoiding wasting raw materials.

## 4. Hole-Transporting Materials as HTLs in OSCs

### 4.1. Metal Oxides

#### 4.1.1. Molybdenum Oxide

**Figure 3:**
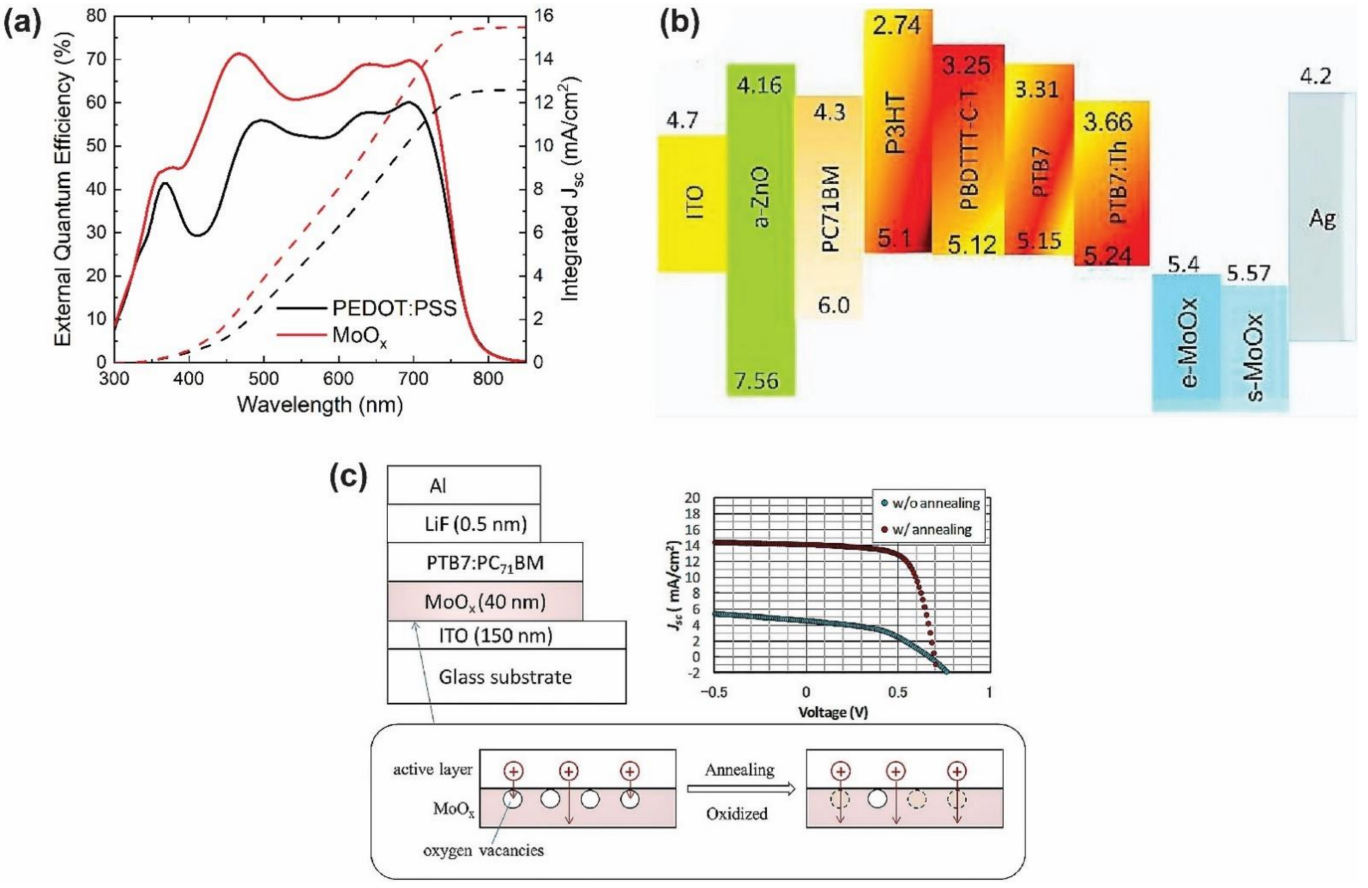
(**a**) External quantum efficiency of inverted OSCs with MoO_3_ and PEDOT:PSS HTLs. Adapted with permission from [98]. (**b**) Energy level diagram of inverted OSCs with different donor polymers. Adapted with permission from [99]. Copyright 2016, Elsevier. (**c**) Reduction of oxygen vacancies by annealing treatment. Adapted with permission from [103]. Copyright 2016, Elsevier.

MoO_x_ is an *n*-type material with a valence band edge around 2.5–3 eV below the Fermi level and a conduction band closer to the Fermi level [70]. MoO_3_ has a high WF (6.9 eV) and conductivity of 1.2 × 10^−7^ Sm^−1^ due to the different states of O and the multivalence of Mo in its three crystal phases (α-MoO_3_, β-MoO_3_, h-MoO_3_) [86,87,88]. MoO_3_ is a promising HTL due to its electronic structure, transparency, conductivity, and stability, enhancing the hole extraction and thus the efficiency of OSCs, compared with PEDOT:PSS [89]. Lee et al. reported that MoO_x_ HTL-based OSCs are more stable at a high operating temperature near 300–420 K than PEDOT:PSS [90]. Therefore, there is much research in strategies to optimize the solution-processing methods and the film properties of MoO_3_ [91,92,93]. Bortoti et al. obtained the orthorhombic phase of MoO_3_ (α-MoO_3_) by refluxing MoS_2_ in HNO_3_ and H_2_SO_4_ as the oxidant media, followed by heating at 120 °C for 10 min to evaporate the solvent [94]. The energy level of α-MoO_3_ well-matched with that of the P3HT. A PCE of 1.55% was obtained in a FTO/ZnO/P3HT:PC_60_BM/MoO_3_/Ag cell structure. Ji et al. used ammonium heptamolybdate (AHM) as the precursor solution to prepare a solution-processed MoO_3_ array on P3HT:PC_61_BM by the ultrasonic spray-coating method at 80 °C [95]. The solution-processed MoO_3_ micro arrays improved the charge transport between the active layer and the anode. Thus, the V_oc_ and FF increased to 0.59 V and 59.2%, and a higher PCE of 3.40% was achieved. MoO_3_ is adequate to attain a high built-in potential and V_oc_ because it can suppress interfacial reactions at the HTL/BHJ interface. MoO_3_ nanoparticles (NPs) can be added at the interface between the active layer and the PEDOT:PSS to take advantage of the localized surface-resonance plasmon (LSRP) effect of NPs and the electronic structure of MoO_3_ [96]. MoO_3_ NPs increased the path length of the absorbed light and blocked the electrons flow to the anode, resulting in a higher J_sc_ and FF, and thus a PCE of 4.11% was reached over a long period of 30 days [97]. The high transparency of MoO_x_ allows an enhanced back-reflected light into the active layer to enhance the photocurrent, as shown in the EQE curves (see Figure 3a) [98]. At low temperatures of 80–200 °C, Jagadamma et al. prepared an alcohol-based MoO_x_ nanocrystalline suspension processed directly over temperature-sensitive active layers (see Figure 3b) [99]. The water-free solvent and the fine MoO_x_ nanocrystal diameter (<5 nm) resulted in a compact and smooth film with a thickness around ~5–10 nm. All inverted OSCs reached a PCE above 9%, retaining 90% of their efficiency after five months of aging. MoO_3_ nanocrystals (NCs) with a size greater than 5 nm can form a composite of MoO_x_ with Ag nanowires (NWs) to lower the nanowire junction resistance by close packing Ag NWs. The Ag NWs/MoO_x_ composite also served as a barrier for Ag diffusion into the active layer’s bulk. Wang et al. added AgAl NPs into MoO_x_ HTL to prevent the Ag diffusion by forming AlO_x_ [100]. The PTB7-Th:PC_71_BM cell retained 60% of the initial PCE (9.28%) over 120 days. Cong et al. used ammonium molybdate and citric acid in 2-methoxyethanol as the precursor to prepare MoO_x_, followed by 10 vol.% of H_2_O_2_ to form a stable conductive film [101]. The presence of H_2_O_2_ induced oxygen vacancies to help in the polyvalence and conductivity of the MoO_x_ film. Jung et al. prepared a solution-processed MoO_x_ from the dissolution of MoO_x_ powder in ammonium hydroxide (NH_4_OH) and isopropanol solvent [102]. The Mo^5+^-OH bonds induced by hydroxyl radicals facilitated the charge transport with higher hole mobilities, of 2.3 × 10^−6^ cm^2^V^−1^s^−1^, than PEDOT:PSS, of 2.1 × 10^−6^ cm^2^V^−1^s^−1^. The gap states induced in the bandgap by the oxygen defects tuned the Fermi level of MoO_x_ with the HOMO of PBDB-T as the donor material, showing overall improvement in FF and J_sc_ with a PCE of 10.86%. The excess of oxygen vacancies during the film formation results in recombination sites which compromise the performance and stability of the OSC [37]. Kobori et al. improved the J_sc_ and FF when the as-deposited solution-processed MoO_x_ film was annealed at 160 °C for 2 min [103]. The enhancement in the efficiency from 1.40% to 6.57% is because of surface passivation of MoO_x_ HTL by annealing treatment, resulting in a reduction of oxygen vacancies in the MoO_x_ film (see Figure 3c). It helps the fabrication of OSCs with temperature-sensitive low-bandgap polymers, such as PTB7-Th:PC_71_BM and PCPDTBT:PC_71_BM. Li et al. reported that low-temperature annealing treatment could also enhance the preparation of solution-processed MoO_x_ films from peroxomolybdic acid organosol precursor solution at 150 °C, while also achieving passivation of the surface [104].

Ultraviolet (UV) annealing can retain a higher PCE over a longer period if compared with OSCs’ efficiency under no annealing or under thermal annealing (100 °C) [105]. UV annealing removed the adhered organic contaminants on the MoO_3_ film surface by two short wave UV lights at 185 nm and 285 nm. This radiation decomposed O_3_ into O_2_ and active O, which oxidized and removed any organic contaminant by transformation into volatile gases. Cai et al. achieved a PCE of 9.27% in the PBDB-T:ITIC BHJ cell using an ultraviolet-deposited MoO_3_ film [106]. Tan et al. developed a solution-processed, annealing-free aqueous MoO_x_ for non-fullerene OSCs [107]. By adding a small amount of water to MoO_2_(acac)_2_, the ligand of MoO_2_(acac)_2_ was removed from the MoO_x_ film, avoiding thermal treatments, and enhancing the PCE of PBDB-T-2F:Y6 cell up to 17.0%. In addition to the impurities in the precursor solution, external factors, such as air, create oxygen defects in the MoO_x_ film lattice, which change the electric properties (e.g., WF, energy levels) and the performance of the OSC [108,109]. Soultati et al. reported the microwave (MW) air annealing approach for recovering the WF in stoichiometric MoO_x_ and the efficiency of the FTO/MW-MoO_x_/P3HT:PC_71_BM/Al cell up to 5.0% [110]. The WF recovery resulted in the formation of a large interfacial dipole at the FTO/MW-MoO_x_/P3HT:PC_71_BM interfaces, favoring hole extraction via gap states.

**Figure 4:**
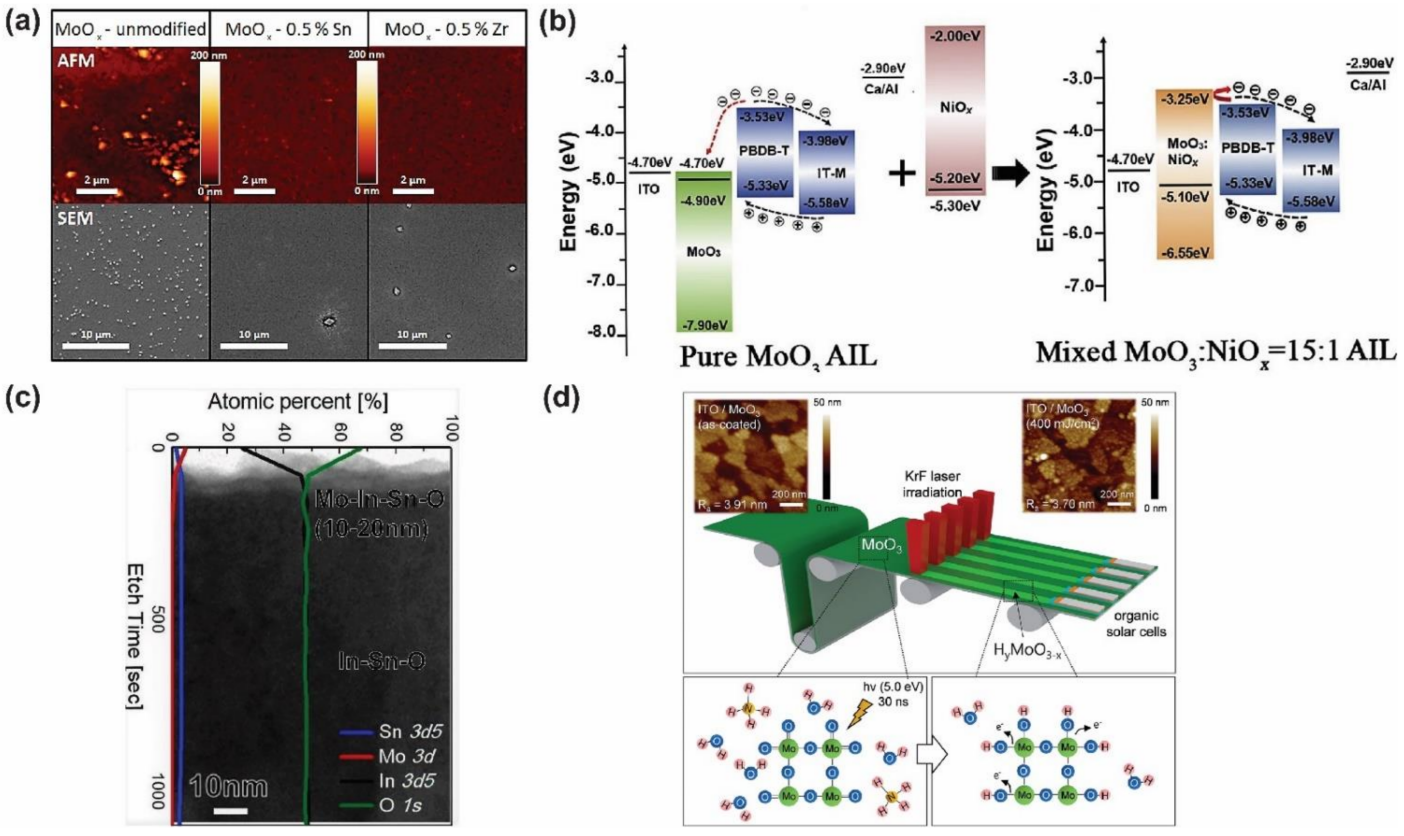
(**a**) AFM and SEM images of unmodified and modified MoOx HTL. Adapted with permission from [112]. (**b**) Energy levels of pristine and doped MoO_3_ with a NiOx layer. Adapted with permission from [113]. Copyright 2019, Elsevier. (**c**) XPS depth profile of MGI electrode. Adapted with permission from [126]. Copyright 2016, Elsevier. (**d**) Scheme of laser-assisted synthesis of H_y_MO_3-x_. Adapted with permission from [129]. Copyright 2016, Royal Society of Chemistry.

In addition to post-treatments, the film properties of the MoO_3_ HTL in OSCs also improve through strategies involving doping, composite/hybrid formation, multilayers, and deposition techniques. Chang et al. reported vanadium-doped MoO_x_ films at different ammonium metavanadate concentrations. The smallest band offset (1.13 eV) between the valence band edge of V_0.05_MoO_x_ and P3HT HOMO level favored the hole transport due to having the lowest resistance among all V-MoO_x_ films [111]. Marchal et al. reported a decrease of 3 nm in the surface roughness of MoO_x_ HTL by adding 0.5 mol% of Zr and Sn via a combustion chemical deposition method at low temperatures [112]. The Zr and Sn atoms also covered the surface defects of MoO_x_, forming a uniform and well-covered HTL film on the ITO electrode (see Figure 4a). Bai et al. employed a small amount of *p*-type NiO_x_ into *n*-type MoO_3_ in one step [113]. Since MoO_3_:NiO_x_ was highly transparent and had a conduction band of 3.25 eV and a WF of 5.10 eV (see Figure 4b), the MoO_3_:NiO_x_ film was able to block electrons while enhancing the contact to charge transport toward the anode, achieving a PCE of 10.81% in PBDB-T:IT-M BHJ OSCs. Li et al. showed the feasibility of the work function tuning of MoO_x_ to use as both HTL and ETL through the Cs intercalation approach [114]. MoO_x_ and the intercalated mole ratio MoO_x_:Cs (1:0.5) tested in P3HT-based conventional and inverted OSCs as HTL and ETL achieved PCEs of 3.50% and 3.20%. Besides, high PCEs of 7.35% and 6% were obtained in the PBDTDTTT-S-T-based conventional and inverted OSCs. The Cs-intercalation within the MoO_x_ acts as an *n*-type semiconductor [115] to tune the work function from 5.30 to 4.16, which favors the energy alignment at the interface and the reduction in the charge carrier losses. Yoon et al. synthesized a dual-HTL by mixing solution-processed copper iodide (CuI) and thermally evaporated MoO_3_ [116]. The interaction between MoO_3_ and the CuI increased the forbidden gap states in the MoO_3_ layer for the hole transport by forming small oxygen vacancies and Mo^5+^ defect states. Zhiqui et al. reported a composite of copper bromide (CuBr_2_) and molybdenum trioxide (MoO_3_) as the HTL for OSCs [117]. CuBr optimized interfacial contact to increase charge carriers, and MoO_3_ blocked electron transport, resulting in improved FF (65.20%), J_sc_ (19.65 mAcm^2^), and an increase in the PCE from 7.30 to 9.56%. Li et al. prepared CTAB-modified MoO_3_ nanocomposites by adding a small amount of cetyltrimethylammonium bromide (CTAB) solution into ammonium molybdate and annealing it at 200 °C in a glovebox [118]. CTAB passivated the surface traps of MoO_3_ films to avoid the recombination sites, resulting in a film with PCEs of 5.80 ± 0.13% in P3HT:ICBA and 8.34 ± 0.13% in PTB7:PC_71_BM OSCs. The formation of polynuclear metal-oxo clusters (PMC) of tungsten/molybdenum as HTLs showed PCEs of 14.3% and higher stability than PEDOT:PSS [119]. The variation in the W/Mo ratio allowed the increase of the hole transport from the polymer donor (PBDBT-2F) toward the anode due to the formation of an inorganic-organic charge transfer complex with a barrier-free interface. This unique characteristic of PCM clusters in OSCs might promote new insights for its utility in high-performance optoelectronic devices. Kwon et al. also boosted the efficiency by developing an alloy of molybdenum-tungsten disulfides films as HTL to replace PEDOT:PSS efficiently [120]. As was mentioned before, Ag NPs can be incorporated into MoO_3_ to enhance the electrical and optical properties of the HTL. Indeed, it can form a MoO_3_/AgNPs/MoO_3_ structure as HTL to improve the J_sc_ and reduce the recombination by the backscattering and surface plasmon effects of AgNPs [121]. Zhang et al. prepared a solution-processed MoO_3_/AgNPs/MoO_3_ (MAM) HTL in PTB7:PC70BM cells [122]. The MAM multilayer enabled an enhanced charge collection by suppressing charge recombination. The efficiency of the OSC was superior (7.68%) to that of the s-MoO_3_ (6.72%). The manufacture of OSCs has also been limited by the material’s finite availability, such as the transparent anode electrode, ITO [123,124]. An ITO-free flexible OSC obtained by Chen et al. used multiple layers of molybdenum oxide MoO_3_/LiF/MoO_3_/Ag/MoO_3_ as transparent electrodes, facilitating the transmittance and charge transport [125]. Lee et al. reported a reduced atomic percentage of In and Sn at the surface of ITO electrodes by graded sputtering of MoO_3_ HTLs (see Figure 4c) [126]. The MoO_3_-graded ITO (MGI) electrode formed three regions: (i) the bottom ITO region, providing high transparency (83.8%), (ii) the Mo-In-Sn-O graded interlayer, and (iii) the MoO_3_ region, which served as the HTL. For thin HTLs, the deposition method might cause defects or form compacted layers depending on the working conditions. Uniform s-MoO_x_ HTLs prepared by direct current (DC) magnetron sputtering showed enhanced charge transport with a FF of 50%, as the s-MoO_x_ film’s surface was smoother and controlled by DC in comparison with the conventional evaporated approach [127]. Chaturvedi et al. applied a DC voltage of 1 kV during the spray deposition of MoO_3_ HTL, obtaining a PCE of 2.71% [128]. The applied electric field controlled both the optical and electrical properties of the thin MoO_3_ film. Dong et al. used a laser-assisted method to obtain a hydrogenated molybdenum oxide H_y_MoO_3−x_ film for flexible OSCs (see Figure 4d) [129]. By controlling the energy of the KrF laser (λ = 248 nm) during the irradiation of photons on the AHM precursor solution, the WF (5.6 eV) and the hole transport of H_y_MoO_3−x_ film increased, allowing higher PTB7:PC_70_BM cell performance. The laser processing time lasts only 30 ns, so it is suitable in time and economically compared with the thermal evaporation method.

#### 4.1.2. Tungsten Oxide

**Figure 5:**
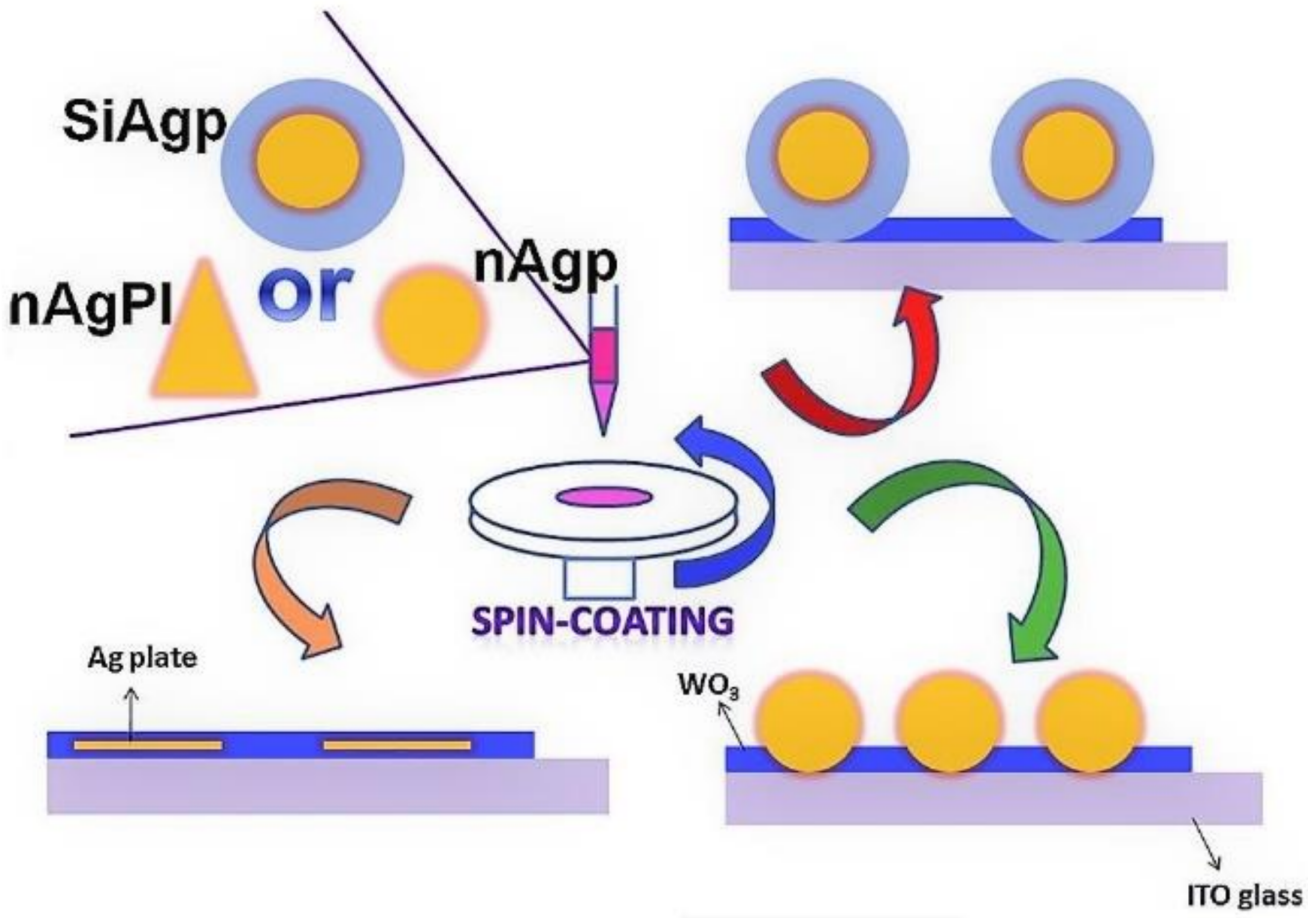
Schematic representation of different silver nanoparticles structures/WO_3_ layers by spin coating. Adapted with permission from [141]. Copyright 2016, Elsevier.

Tungsten oxide is an *n*-type material with a WF ranging from 4.7 to 6.4 eV depending on the film preparation [130,131,132,133]. Tungsten oxide is a hole extracting layer that can work efficiently in conventional and inverted OSCs using vacuum and solution-processing methods [74,134]. WO_x_ is an amorphous structure that (i) forms smooth surface morphologies, (ii) increases the charge mobility in the active layer, and (iii) enhances the charge collection because V_oc_ depends linearly on the anodic WF when there is not ohmic contact at the anode/donor interface [135]. Thus, the enhancement in solution-processing WO_x_-based OSCs is particularly focused on increased light absorption. Lee et al. designed an Au@SiO_2_-WO_3_ nanocomposite (NC) which works as a photon antenna for high light absorption [136]. The localized surface-plasmon resonance (LSPR) effect of AgNPs enhances the intensity of photon absorption in the P3HT:PC_61_BM BHJ cell, resulting in increased J_sc_. The favorable plasmonic effect is comparable to some reported literature for plasmonic nanomaterials-based optoelectronic devices [137,138,139]. Moreover, high hole mobility of WO_x_ NPs boosted the device PCE by up to 1.6%. The surface morphology of the Au@SiO_2_-WO_3_ NC film was kept uniform due to the SiO_2_ shell avoiding the aggregation effect of the Au NPs. Instead of SiO_2_, the aggregation effect can be avoided by controlling the concentration of Au NPs. Using 10 wt% of Au NPs, the Au-WO_3_ NC HTL decreased the surface morphology’s roughness, achieving a PCE of 60.37% [140]. Shen et al. enhanced the light absorption and the PCE of OSCs based on the LSRP effect of structure-differentiated silver nano-dopants in solution-processed WO_x_ HTL [141]. Three silver nano-dopants, (i) naked Ag NPs (nAgp), (ii) SiO_2_-covered Ag NPs (SiAgp), and (iii) naked Ag nanoplates (nAgPI), were synthesized. The triangular nAgPl reached the highest PCE of 4.6% while spherical nAgp reached the lowest. The spherical nAgp surface decreased the PCE because its surface can directly contact the donor/acceptor material of the active layer, resulting in excitons quenching and thus weakening LSRP effects (see Figure 5). The shape of NPs affects the overall performance of OSCs by tuning plasmon-electrical [142], plasmon-optical [143,144], and charge-storage effects [145]. Ren et al. reported the high efficiency of OSCs by incorporating gold nanostars (Au NSs) between HTL and the active layer [146]. The plasmonic asymmetric modes of Au NSs enhanced the optical absorption of the active layer and the balance of photogenerated charges by shortening transport path length in the HTL. The localized plasmonic effect of NPs manipulates transport paths of photogenerated carriers in bulk heterojunction OSCs and thus reduces the charge recombination sites and the space-charge-limit effect [147,148]. Li et al. reported comparable results by applying Ag nanoprisms to achieve higher PCE through the improvement in the broadband absorption [149]. Remya et al. performed a study between dehydrated and di-hydrated WO_3_ films as HTL in the inverted P3HT:PC_61_BM and PTB7:PC_71_BM cells [150]. The hydrated phase of WO_3_ enabled a suitable energy level alignment with the active layer by tuning the water coordination, resulting in a higher PCE of 5.1% and 7.8%, respectively.

#### 4.1.3. Vanadium Oxide

V_2_O_5_ is a hole-transporting/electron-blocking layer that acts as a protecting layer [151] avoiding surface reactions by the moisture from the working conditions, resulting in improved efficiency and stability [152]. The electronic structure of V_2_O_5_ corresponds to an *n*-type material with deep electronic states and WF ranging from 4.7 eV to 7.0 eV, depending on the processing method [73,153]. Li et al. reported the Cs-intercalation method to tune the work function of V_2_O_x_ and used Cs-intercalated V_2_O_x_ and V_2_O_x_ as both ETLs and HTLs in organic optoelectronic devices [114]. The work function tuning and the reduction in the interfacial barrier of Cs-intercalated V_2_O_x_ allowed for obtaining PCEs in P3HT and PBDTDTTT-S-T-based conventional and inverted OSCs up to 3.59% and 7.44%. Xu et al. reported a low-temperature solution-processed V_2_O_5_ by dissolving V_2_O_5_ powder into water at room temperature [154]. V_2_O_5_-based HTLs showed a PCE of 8.05% in ITO/V_2_O_5_/PTB7:PC_70_BM/LiF/Al OSCs compared with PEDOT:PSS-based HTLs with a PCE of 7.46%. V_2_O_5_ served as an optical spacer that increased light absorption, leading to a higher photocurrent. V_2_O_5_ powder can also be treated directly from the melting-quenching sol-gel method to obtain an easy tunable V_2_O_5_·nH_2_O HTL [155]. The energy positioning of the V_2_O_5_·nH_2_O HTL (with n=1) was closer to PEDOT:PSS [67], allowing an ohmic contact with the novel conjugated polymer donor (PBDSe-DT2PyT) and the acceptor of P_71_CBM; thus, a large V_oc_ and a PCE of 5.87% were obtained. The layered and hydrated phase of V_2_O_5_ is an affordable and tunable charge transport material. Although V_2_O_5_·H_2_O-based HTLs exhibit better performance than PEDOT:PSS-based HTLs, the melting–quenching sol-gel method might be an expensive method owing to the high melting temperature of V_2_O_5_ (~800°C). Cong et al. applied a green method to prepare vanadium oxide hydrate layers (VO_x_·nH_2_O) to enhance the PCE in organic PTB7-Th:PC_71_BM- and P3HT:PC_61_BM-based polymer solar cells up to 8.11% and 3.24% [156]. The combined H_2_O_2_ and ultraviolet ozone (UVO) in-situ treatments allowed for a smooth surface and improved wettability with the presence of dangling bonds on the HTL surface to enhance interfacial contact. The presence of V^4+^ in the composition analysis of VO_x_·nH_2_O accounted for a small amount of oxygen vacancies, causing *n*-type doping, which is essential to hole transport by extracting electrons through its conduction band [157]. Vishnumurthy et al. reported that V_2_O_5_ HTL optimized the efficiency of thienothiophene-diketopyrrolopyrole-based OSCs by up to 1.02% [158]. Remya et al. prepared an efficient hole-transport/electron-blocking hydrated vanadium oxide (HVO) from V_2_O_5_ powder with hydrogen peroxide [159]. In the P3HT:PC_61_BM and PBDTT-FTTE:PC_71_BM BHJ cells, HVO HTL performance was superior to PEDOT:PSS, obtaining 56% enhancement (7.12–11.14%) in the PCE for the PBDTT-FTTE:PC_71_BM-based inverted OSC with a lower degradation of 1.4% over 20 weeks. In addition to the V_2_O_5_ powder, V_2_O_5_ HTLs can be prepared by other precursors. Xu et al. reported an ammonium metavanadate ammonal water solution for processing VO_x_ HTLs in PTB7:PC_71_BM BHJ cells with a PCE of 7.7% [160]. This HTL showed a WF of 5.3 eV and high conductivity by air-annealing treatment at 210 °C for 5 min. The thermal treatment smoothed the surface film to reduce the leakage current, obtaining a higher J_sc_. Although the stability was better than PEDOT:PSS with a remaining 83% efficiency after four days, it was still low compared with other inorganic HTLs. Shafeeq et al. reported the formation of uniform and crystalline V_2_O_5_ nanorods by thermal decomposition of ammonium metavanadate NH_4_VO_3_ to enhance surface morphology and efficiency of OSCs [161]. Alsulami et al. obtained a stable V_2_O_x_ HTL by using vanadium (V) oxytriisopropoxide as the precursor, which converted into V_2_O_x_ by hydrolysis in air [162]. The PCE of the V_2_O_x_ HTL was insensitive to thermal annealing at 100 °C and 200 °C because its optical and electronic properties were comparable to the vacuum-deposited V_2_O_5_. Besides, the highly tunable V_2_O_5_ thin films prepared by the solution-processing method boost inverted OSCs because of their higher stability under air conditions [163]. To optimize the interface properties and OSC performance, VO_x_ NP can efficiently be mixed with PEDOT:PSS solution, resulting in a stable VO_x_:PEDOT:PSS HTL by the uniform molecular distribution of VO_x_ with PEDOT:PSS as reported by Teng et al. [164]. They achieved a PCE, of 10.2%, compared with PEDOT:PSS, of 5.27%, when VO_x_:PEDOT:PSS was used as HTL in the TPD-3F:IT-4F cells. Xia et al. reported a nanoparticulate compact V_2_O_5_ film as HTL using a facile metal-organic decomposition method to replace the traditional HTLs [165]. By adding polyethylene glycol (PEG) as an additive in the precursor, a uniform and compact film of V_2_O_5_ served as HTL in the PTB7:PC70BM, improving the interface contact, J_sc_, and the FF. Compared with the spin coating, the spray coating of V_2_O_5_ HTL has allowed the large-scale production of flexible OSCs in a roll coater [166]. Using a precursor solution of vanadium oxytriisopropoxide (VTIP) diluted in ethanol (1:100), V_2_O_5_ HTLs exhibited improved electrical properties. The mechanical stress on V_2_O_5_ HTL was mitigated by introducing a PEDOT:PSS binding-interfacial layer between V_2_O_5_ HTL and the Ag electrode in the inverted P3HT:PC_60_BM and PBDTTTz-4:PC_60_BM BHJ cells. Arbab and Mola also explored electrochemical deposition that resulted in 80% enhancement in PCE (2.43%) compared with PEDOT:PSS-based OSCs [167]. Kavuri et al. reported electrospray deposition (ESD) for V_2_O_5_ HTL in PTB7:PC_71_BM-based OSCs with a PCE of 7.61% [168]. Compared to the spin-coating, the ESD allowed more control in the deposition conditions and reduced the manufacturing costs of V_2_O_5_-based OSCs. Surface morphology, charge mobility, and interfacial contact were adjusted as a function of the solvent evaporation rate. V_2_O_5_ HTL has also been effective in ITO-free polymer solar cells with an optimized precursor solution (VTIP) of 0.005% [169]. The deposition of V_2_O_5_ HTL on PEDOT:PSS, as the anode, led to increase R_sh_ and conductivity with the active layer of P3HT:PC_61_BM by the hydrophobic surface of V_2_O_5_, resulting in an uniform and compact HTL with a PCE of 3.33%. V_2_O_5_ is also a potential material that increases the anode’s WF of indium zinc oxide (IZO), exhibiting a higher PCE of 2.8% than that flexible OSCs with only IZO [170].

#### 4.1.4. Nickel Oxide

**Figure 6:**
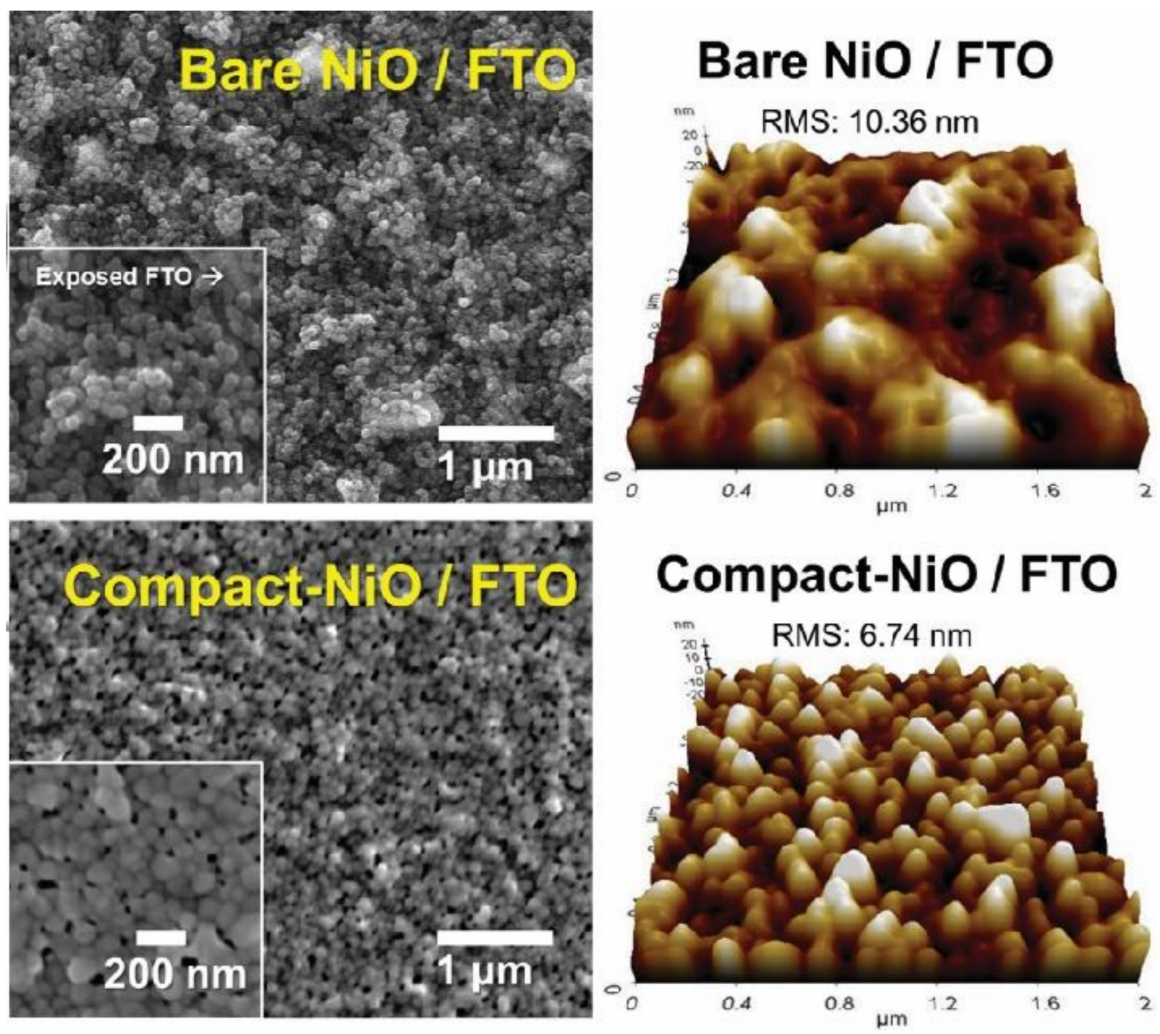
AFM and SEM images of bare nickel oxide on FTO (without PEG) and compact nickel oxide (c-NiO) on FTO (with PEG). Adapted with permission from [178].

Non-stoichiometric NiO_x_ is a wide bandgap *p*-type semiconductor [171]. NiO_x_ is an efficient electron-blocking layer to the anode due to its conduction band minimum, 1.8 eV, which is above the LUMO of the organic donor P3HT (3.0 eV) [75]. Due to the conduction band of NiO_x_ being closer to the vacuum level, it is able to suppress electron recombination at the anode [172]. The ohmic contact between NiO and P3HT allows holes to freely transport from the active layer to the anode through the Ni^2+^ vacancy-based hole-conducting anode band [173]. Parthiban et al. demonstrated an enhancement in OSC performance with a NiO HTL deposited via spin coating [174]. Using the precursor solution of nickel acetate and a simple post-annealing process (>300 °C) to reduce roughness, NiO HTL achieved a FF of 63.0% and a corresponding PCE of 4.45% in RP(BDT-PDBT):PC_70_BM solar cells. Although NiO-based HTLs exhibit better performance and stability than PEDOT:PSS, the high annealing temperature required to convert the nickel precursors into the NiO thin films make it expensive and not compatible with flexible substrates. Chavhan et al. reported a room-temperature approach to manufacture NiO_x_ films from a nickel formate precursor solution via UV-ozone treatment [175]. In terms of efficiency, the UV-ozone treatment results were ideal for increasing the WF by creating hydroxides at the surface, avoiding high processing temperatures. A high PCE of 6.1% in NiO_x_ HTL treated with UV-ozone was related to increased presence of NiO(OH) at the surface. Besides the precursor method, Jiang et al. used chemical precipitation to obtain non-stoichiometric NiO_x_ NPs at room temperature without any post-treatment [176]. The atomic ratio between Ni and O (1:1.14) reduced the R_s_ of the opto-electronic device as the *p*-type conductivity was enhanced by the presence of two oxidation states (Ni^2+^ and Ni^3+^) that favor Ni^2+^ vacancies. Thus, the FF and the J_sc_ increased up to 67.20% and 9.67 mAcm^−2^ to yield a PCE of 3.81% in P3HT-based conventional OSCs. The high performance of NiO_x_ NPs HTL-based OSCs was also demonstrated for low-bandgap polymers. Alternatively, *p*-type ternary metal oxides are promising candidates for enhancing electron-blocking ability due to their tunable electronic and optical properties through the hypocrystalline hydroxide-based method [177]. To date, the high surface roughness of fluorine-doped tin oxide (FTO) has limited its application in OSCs; however, the surface roughness can be decreased from 10.36 nm to 6.74 nm by fully covering it with an optimized NiO layer (see Figure 6) [178]. A polyethylene glycol (PEG) assisted sol-gel process altered the c-NiO/FTO surface because it has a stabilizing effect on NiO NPs, so it allowed the crystallization of a close-packed structure of NiO film. The further deposition of PEDOT:PSS led to the formation of a free-pinhole layer with an RMS roughness of 2.44 nm and selective hole transport, increasing the PCE from 5.68% to 7.93%. Although organic devices based on spin-coated NiO HTLs have emerged successfully in the photo-electronic field, it is vital to focus research efforts for printing technologies for large-area roll-to-roll production. Printing technology usually results in thick NiO films, increasing the interfacing between the active and HTL layers and shortening hole carriers’ migration due to its short lifetime [179]. Singh et al. obtained a thin film of NiO_x_ by controlling substrate-processing conditions and inkjet printing [180]. Optimal conditions of UVO pretreatment, drop spacing, and substrate temperature at 25 °C resulted in a PCE of 2.60% in the P3HT:PC_60_BM cell with superior environmental stability. Huang et al. used copper (5.0 at.%) as a dopant to increase the electrical conductivity of NiO_x_ film, resulting in a reduction of R_s_ from 11.25 to 9.98 Ωcm^2^ [181]. The Cu-doped NiO_x_ (Cu:NiO_x_) also improved the interface contact with the active layer and facilitated the charge transport, resulting in a higher PCE of 7.1% in PCDTBT:PC_71_BM-based cells. The enhancement in the optoelectronic properties, surface morphology, and stability of NiO_x_ HTL by doping is comparable with reported literature, as observed in the co-doping of NiO_x_ NPs with Li and Cu [182]. The co-doping favored the conductivity by increasing the Ni^3+^/Ni^2+^ ratio and kept the high transparency in the well-dispersed solution based on NiO_x_ NPs.

#### 4.1.5. Other Oxides

**Figure 7:**
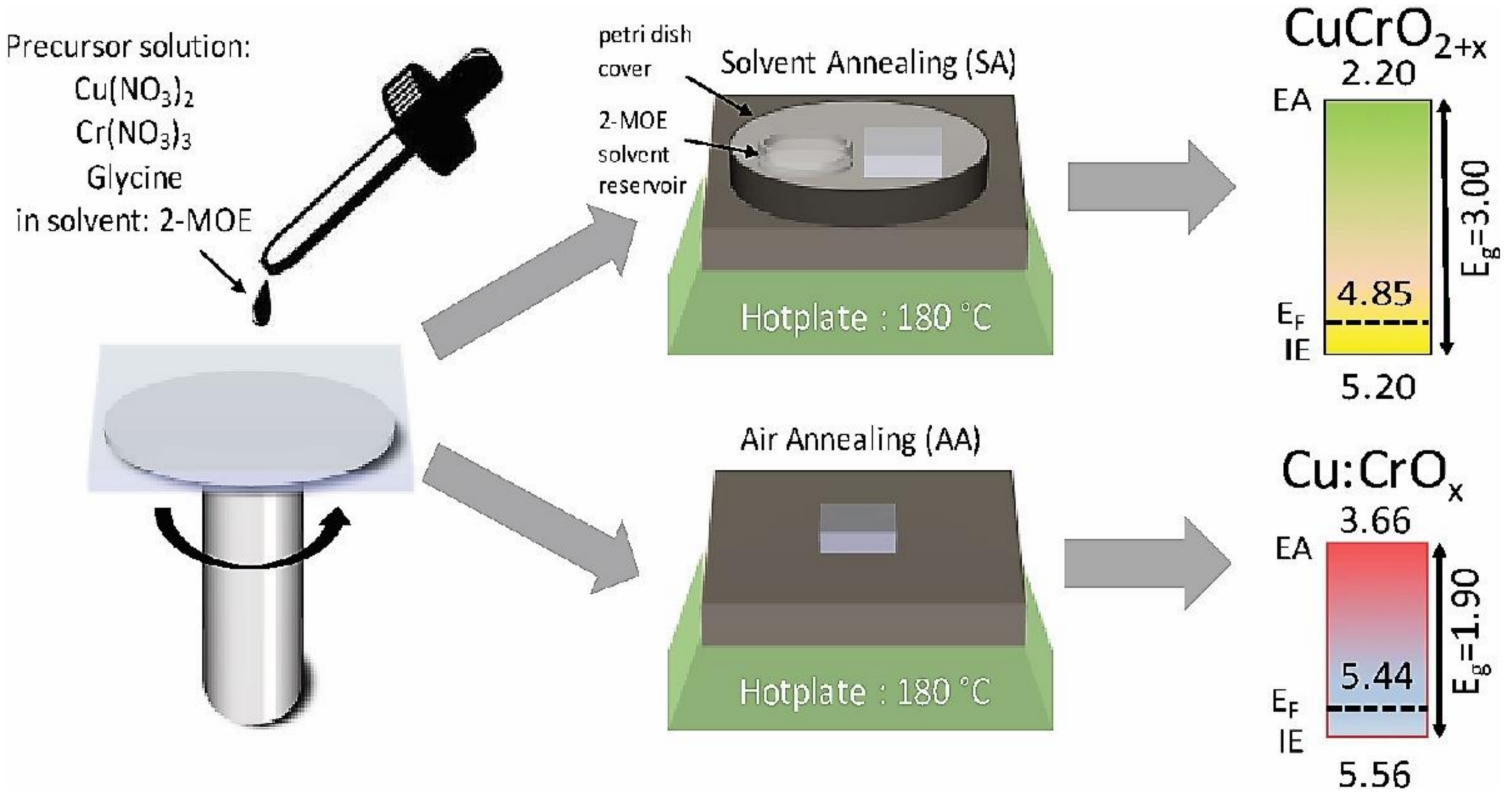
Schematic representation of the combustion synthesis. Adapted with permission from [194]. Copyright 2018, American Chemical Society.

CuO_x_ are *p*-type semiconductors with narrow band gaps of 1.3–2.0 eV for CuO and 2.1–2.3 eV for Cu_2_O [183,184,185,186]. HTLs of CuO_x_ spin-coated on ITO decreased the interfacial barrier using a green solvent of copper acetylacetonate (Cu(C_5_H_7_O_2_)_2_), improving cell efficiency of PTB7:PC71BM cell up to 8.68% [187]. After H_2_O_2_ and UVO treatment, CuO_x_ HTLs increased the WF to 5.45 eV, forming an excellent ohmic contact, while the V_oc_ increased to 0.74 V. Furthermore, the oxidation of CuO_x_ by UVO treatment enhanced the interfacial contact and the light absorption in the visible range, obtaining a high transmittance of 88%, low R_s_ of 2 Ωcm^2^, and higher hole transport to the anode. The OSCs’ initial performance (8.68%) dropped down to 47% over 50 h of storage in the air. The *p*-type CuCrO_2_ is a semiconductor that belongs to the delafossite compounds [188]. CuCrO_2_ HTLs are of great interest in optoelectronic applications due to their high transparency, large hole diffusion coefficient, high WF, and ionization energy, which are essential in the manufacture of OSCs [189,190,191]. Other strategies to boost the potential of CuCrO_2_ HTL involve In doping, in which optical transmittance and hole conductivity are increased [192]. New alternative techniques to produce efficient and cost-effective CuCrO_2_ HTLs for roll-to-roll manufacturing are developing, such as microwave assisted-heating to produce CuCrO_2_ nanocrystals with an efficient PCE of 4.9% [193], or the combustion synthesis to produce CuCrO_2_ thin films by low-temperature processing at 180 °C with a PCE of 4.6% (see Figure 7) [194]. Both methods are highly efficient and represent advances for lowering fabrication costs. UV-ozone post-treatment or annealing increases the metallic copper oxidation to Cu^+2^ to promote the electronic conduction by the hopping mechanism between Cu^1+^ and Cu^+2^ species. The higher oxidation state of Cu^2+^ enhanced the electronic properties, exhibiting deeper ionization energy (IE) and Fermi energy (E_F_). The Cu doping favored the surface-roughness reduction, resulting in an improved interfacial contact, and thus favored J_sc_, FF, and PCE.

Wahl et al. reported the first HTL based on ITO NPs in inverted OSCs [195]. The addition of ethylenediamine into ITO NPs stabilized it to deposit uniform HTLs on the underlying absorber layer. The deposition of the ITO NPs HTLs by doctor blading allowed controlling the thickness between 15 and 20 nm. Post-treatments of thermal annealing and plasma were beneficial for the film’s electronic properties, achieving a PCE of 3%. However, plasma application needs to be mild to avoid OSCs’ detrimental performance. The doping method using high-WF metals might be a good alternative over plasma treatments to develop high-quality films in OSCs. The solubility of metal oxides in common solvents such as DMF or water is another main factor for its application in the roll-to-roll manufacturing of OSCs. Bhargav et al. reported the suitability of DMF-soluble Co_3_O_4_ as HTLs in PCDTBT:PC_71_BM BHJ [196]. Co_3_O_4_ HTLs showed transparency around 81% and a smooth surface, allowing for a remarkably high FF of 49.1% and higher PCE (3.21%) compared with PEDOT: PSS-based OPVs.

### 4.2. Metal Sulfides

#### 4.2.1. Molybdenum Disulfide

**Figure 8:**
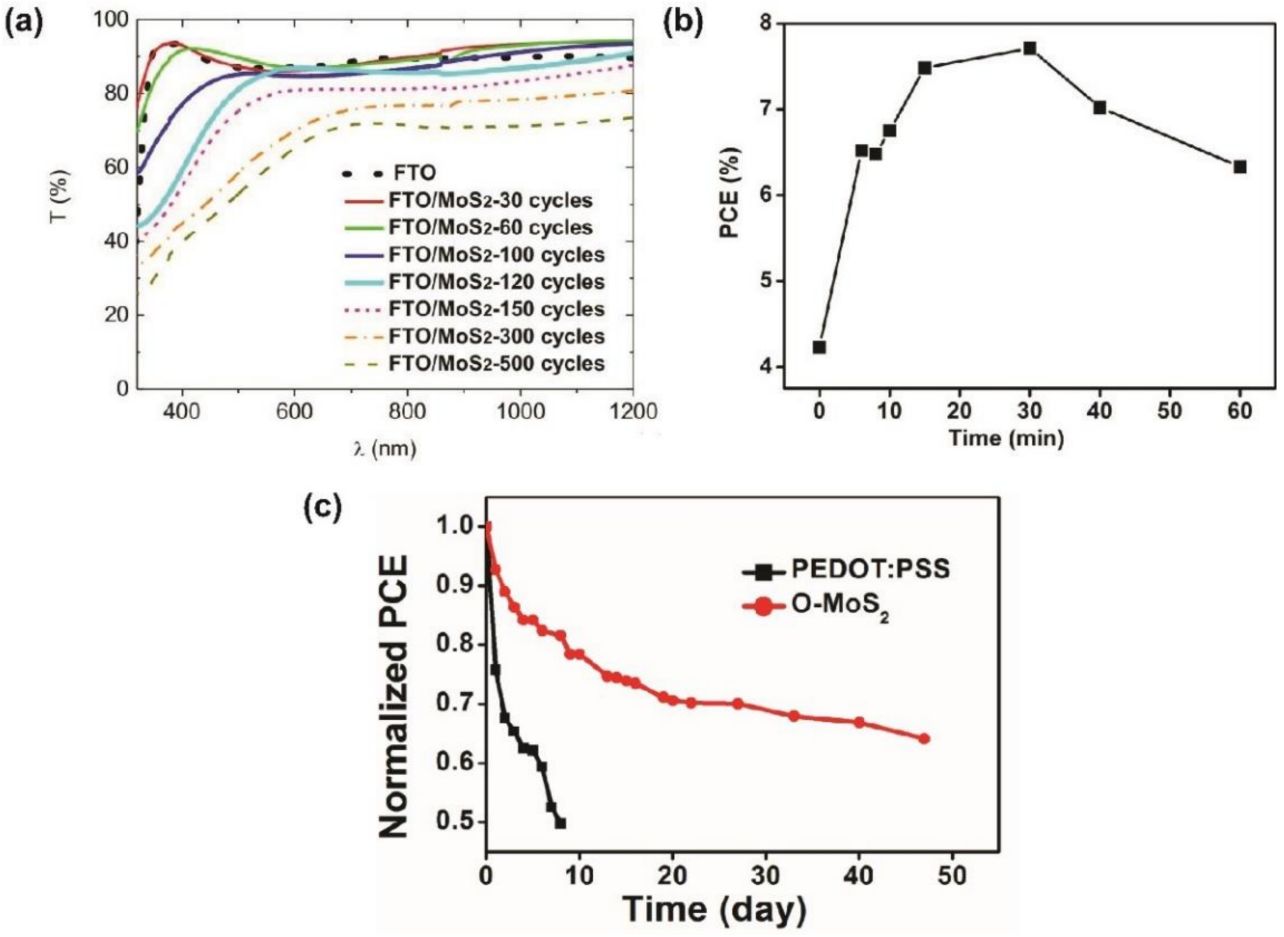
(**a**) Transmission spectra of FTO/MoS_2_ at various numbers of scan cycles. Adapted with permission from [199]. Copyright 2017, Elsevier. (**b**) Dependence of PCE on the duration of the UVO treatment and (**c**) stability of devices using UVO-MoS_2_ QDs compared with PEDOT:PSS. Adapted with permission from [200]. Copyright 2016, American Chemical Society.

MoS_2_ with a layered structure is a metal dichalcogenide (TMD) semiconductor that can display two phases under normal conditions, the traditional trigonal prismatic H-MoS_2_ phase and the distorted octahedral ZT-MoS_2_ phase with hole mobilities of 3.8 × 10^2^ cm^2^V^−1^s^−1^ and 5.7 × 10^4^ cm^2^V^−1^s^−1^, respectively [197]. Instead of using a vacuum or temperature-dependent process to prepare the traditional MoS_2_ HTL, Barrera et al. prepared suspensions of MoS_2_ via liquid exfoliation at room temperature [198]. The high WF of MoS_2_ resulted in enhanced charge mobility; however, the low transmittance of the film affected the J_sc_. An effective way to address films’ low transmittance is by using composites or hybrid layers with tunable transparency. Martinez-Rojas et al. reported a hybrid layer of MoS_x_:MoO_3_ on FTO substrates with high transmittance by a pulsed electrochemical method [199]. After 150 cycles of depositing MoS_x_ on the MoO_3_, the percentage of transmitted light decreased significantly due to the agglomeration of MoS_x_ (see Figure 8a). A hybrid layer with 100 cycles of deposition resulted in 10% higher PCE than the one obtained using MoO_3_ or MoS_x_ HTL. MoS_x_ was an efficient electron-blocking layer, while MoO_3_ increased conductivity, resulting in enhanced hole-transporting properties. The effectiveness of UVO treatment to form homogeneous films and increase the WF was tested in a layer of MoS_2_ quantum dots (QDs), showing a PCE of 2.62% and 8.7% for P3HT and PTB7-Th donor systems [200]. The solar cell efficiency increased after 30 min of UVO exposure, but longer UVO treatment periods degraded the HTL, resulting in decreased PCEs (see Figure 8b). The UVO-MoS_2_ QDs showed compact and uniform layers with a lower surface roughness of 1.19 nm than UVO-MoS_2_ nanosheets of 2.03 nm. The OSC achieved long-term durability due to the improved interfacial contact, showing 64% of its initial PCE after 47 days (see Figure 8c). Annealing treatments can also decrease the surface roughness and favor the optoelectronic properties of the film. At 300 °C, MoS_x_ flatted the surface morphology, enhancing the PCE by up to 7.5%, 52% of which was retained after two months [201]. However, an annealing treatment is not as efficient as a UVO treatment for temperature-sensitive devices.

#### 4.2.2. Tungsten Disulfide

Adilbekova et al. used a liquid-phase exfoliation technique to manufacture WS_2_ HTL using aqueous ammonia that does not require high-temperature post-treatments [202]. Stabilizers or post-processing treatments were excluded from obtaining WS_2_ nanosheets since stoichiometric quality and structural properties were unchanged after performing the top-down method. Due to the *p*-type character of the 2D nanosheets, the HTLs were selective to hole transport toward the anode, achieving a PCE of 15.6% in the PBDBT-2F:PC_71_BM BHJ cells. Following the same line, Lin et al. fabricated uniform WS_2_ layers on ITO [203]. WS_2_ flakes were wider and thinner than MoS_2_, covering the whole surface of ITO. The surface coverage was dependent on the shape and size of the selected material obtained by the exfoliation procedure and its interaction with the substrate. WS_2_-based HTL in ternary BHJ OSCs (PBDB-T-2F:Y6:PC_71_BM) increased PCE by up to 17%. Ram et al. demonstrated that the use of WS_2_ as HTL increased the PCE of PBDB-T-2F:Y6:SF(BR)_4_ ternary cells by 20.87% [204]. The low hygroscopic nature and low acidity of WS_2_ reduced the contact resistance between the active layer and the ITO.

#### 4.2.3. Nickel Sulfide

**Figure 9:**
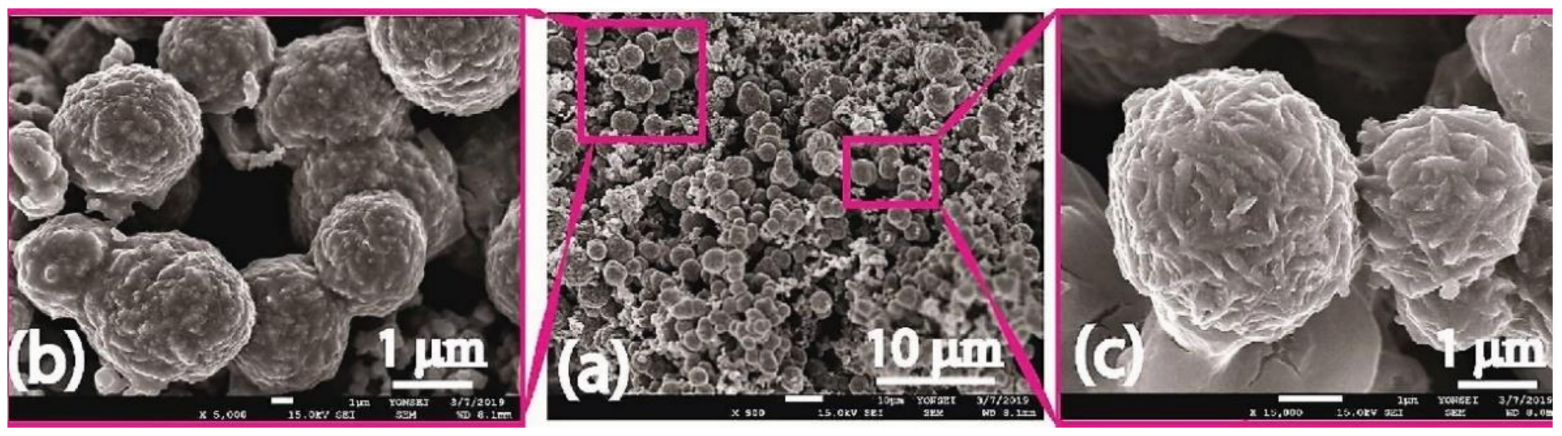
FE-SEM images of globular flower-shaped NiS at magnifications of (**a**) 10 µm and (**b**,**c**) 1 µm. Adapted with permission from [205]. Copyright 2019, Elsevier.

Taking advantage of the dependence of the phase diagrams of NiS with the sulfur content, Hilal and Han synthesized the hexagonal phase of NiS as HTL in OSCs processed by the simple solvothermal method at room temperature [205]. The surface morphology of NiS was smoothened by increasing the sulfur content to 2 g, forming a globular flower-like NiS morphology with increased surface area (see Figure 9). In addition to the enhancement in the hole transport, NiS stabilized the OSC; hence, P3HT:PCBM-based cells retained 26% of their initial efficiency value after 15 days.

#### 4.2.4. Other Sulfides

**Figure 10:**
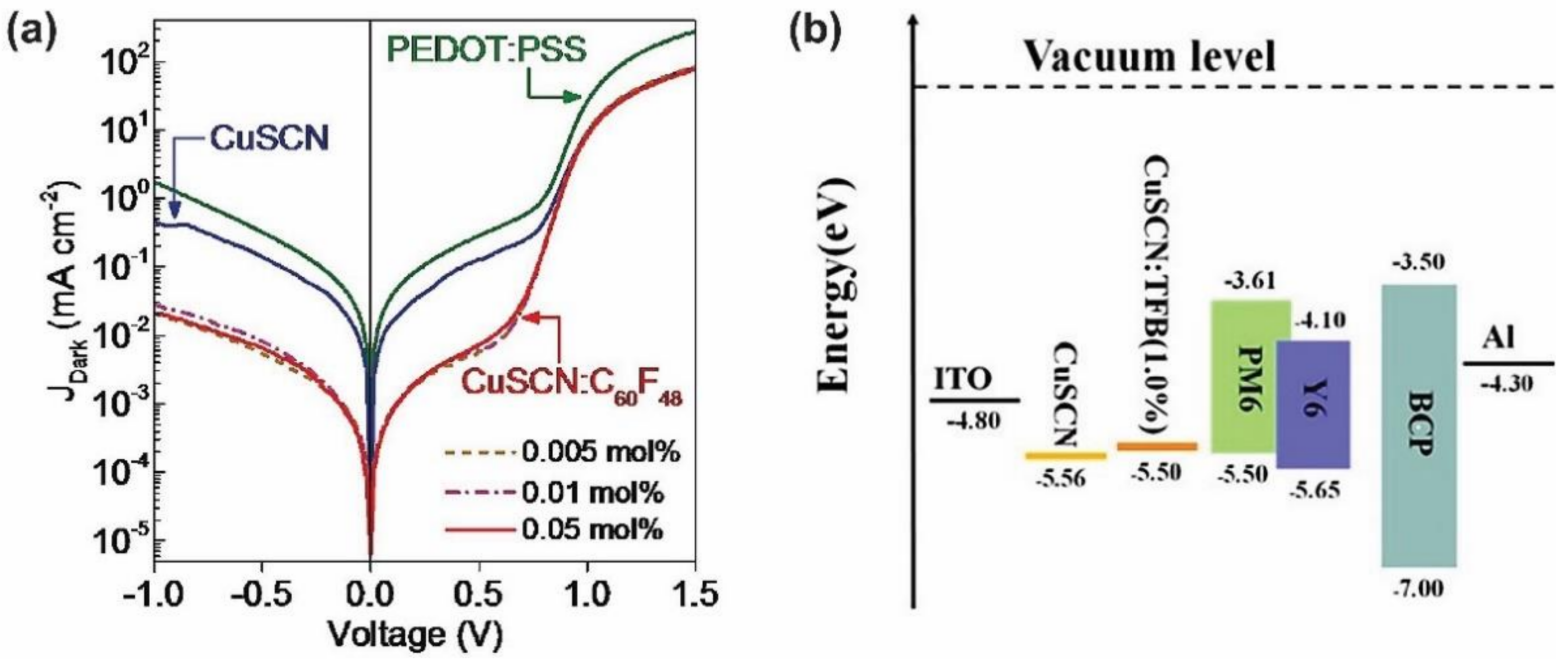
(**a**) J-V curves of CuSCN:C_60_F_48_ under dark conditions. Adapted with permission from [213]. Copyright 2018, John Wiley and Sons. (**b**) Energy level alignment of ITO/CuSCN, CuSCN:TFB(1.0%)/PM6:Y6/BCP/Al OSCs. Adapted with permission from [214]. Copyright 2020, American Chemical Society.

An efficient OSC was achieved by Bhargav et al. using an inorganic HTL made of CuS by a low-cost and efficient manufacturing process [206]. CuS thin films were deposited onto ITO by a solution process instead of vacuum deposition, resulting in a high transparency of 84%. Due to the decreased ohmic resistance, the device structure ITO/CuS/PTB7:P_71_BM/Al reached a high PCE of 4.32% due to the improved FF of 50.1%. A new room-temperature method known as Successive Ionic Layer Adsorption and Reaction (SILAR) was reported by Jose et al. to produce efficient *p*-type Zn-doped CuS HTLs [207]. Due to the high conductivity and low light absorption in the visible region, a PCE of 1.87% was obtained with enhanced charge mobility of 1.5 cm^2^ V^−1^s^−1^. The use of 2D materials like antimonene quantum dots (AMQS) in HTLs has emerged in OSCs production due to their facile synthesis and unique properties [208]. Wang et al. reached an enhanced PCE of 8.8% by the surface passivation of copper(I) thiocyanate (CuSCN) HTL with AMQSs [209]. The AMQSs smoothened the film surface of CuSCN, tuned the WF, and raised the exciton generation rate from 8.79 × 10^27^ m^−3^S^−1^ to 9.95 × 10^27^ m^−3^S^−1^. Compared with PEDOT:PSS HTLs, CuSCN/AMQSs HTLs were more stable at room temperature, retaining 68% of the initial PCE over 1 month not only in fullerene systems such as PTB7- Th:PC_71_BM, but also in non-fullerene systems. Other strategies involving triple-interface passivation [210], multifunctional interface layer using lead sulfide quantum dots (QDs) [211], and self-polymerization of the monomer have been also reported to passivate surface roughness and interface defects [212]. The surface passivation is key in the construction of OSCs to reduce non-radiative recombination losses which in turns affect the charge separation rate once excitons achieve the donor/acceptor interface, resulting in a low V_oc_ and FF. The *p*-doping of CuSCN with C_60_F_48_, an electron acceptor, is an effective method to obtain highly conductive HTLs for its application in OSC devices [213]. By adding 0.5 mol% of C_60_F_48_ that also acts as a nucleating agent, the CuSCN:C_60_F_48_ film was more dense than the pristine CuSCN surface. Moreover, reduced surface roughness, leakage current (see Figure 10a), and improved hole mobility of 0.18 cm^2^V^−1^s^−1^ were attributed to the percolation conduction mechanism, resulting in a PCE of 6.6% in the PCDTBT:PC_70_BM-based OSCs. Wang et al. achieved a PCE of 15.28% in OSCs based on the non-fullerene PM6:Y6 blend by doping CuSCN film with 1% of TFB (see Figure 10b) [214]. Worakajit et al. increased the hole mobility in CuSCN from 0.01 to 0.05 cm^2^V^−1^s^−1^ by passivating surface morphology and the crystallinity with diethyl sulfide (DES) molecules and acetone as antisolvent treatment [215]. Suresh Kumar et al. succeeded in fabricating Cu_2_CdSnS_4_ (CCTS) HTLs over ITO substrates deposited by spin coating at room temperature [216]. A PCE of 3.63% in the P3HT:PC_71_BM blend was achieved by controlling the distribution particle size. The bandgap decreases with an increase in the size of CCTS and layer thickness. Minimum surface roughness of 11.07 nm was found after deposition of three layers of CCTS thin films, improving the thin film’s compactness, hole-transport efficiency, and stability in environmental conditions.

### 4.3. Nanocarbons

#### 4.3.1. Graphene Oxide

Nanocarbon materials like graphene have been applied as HTLs in OSCs due to their unique electrical, optical, and structural properties [217]. Due to the low water dispersibility caused by the nonpolar sp^2^ hybridized carbon structure, the oxidized form of graphene, graphene oxide (GO), has also been used in OSCs by the high solubility in eco-friendly water solvents [218]. The hydroxyl groups and epoxy groups located in the basal plane of the graphene sheet and carboxylic acids at the edge limit the conductivity of GO [219]. In fact, an excess of 25% of oxygen atoms on the GO sheet’s surface reduced its conductivity until it became an insulator material [220]. Thus, it is crucial to control the concentration and thickness of GO for suitable performance as HTLs. Rafique et al. tested the thickness and concentrations of spin-coated GO, selecting 1 mg/mL to form thin conductive films in BHJ OSCs with a PCE of 2.73% [221]. The reduction process is another feasible way to increase the conductivity of GO layers. The reduction removes the excess of oxygen atoms from the GO surface and recovers the conjugated honeycomb structure [222]. Huang et al. succeeded in synthesizing eco-friendly reduced graphene oxide (rGO) by using a modified Hummer’s method to produce GO and thermal treatment to reduce it [223]. A mild temperature of 280 °C was used to obtain rGO and enhance OSCs’ conductivity based on P3HT:PC_71_BM and PTB7:PC_71_BM with a PCE of 3.39% and 7.62%, respectively. The dispersibility must be controlled to ensure good coverage of the underlying substrate. Lee et al. mixed highly dispersible semiconducting fullerenol surfactant with GO, obtaining water-dispersible and conductive films [224]. The conductivity increased from 5 × 10^−4^ Scm^−1^ for the pristine GO layer to 1 × 10^−2^ Scm^−1^ for the fullerenol-GO layer, resulting in a PCE of 3.15%. Chemical and physical methods involving the reduction of GO seek to tune the WF, improve electrical properties, reduce absorption, and increase hole mobility and charge collection capability. Kwon et al. obtained rGO by electron-beam irradiation with shorter processing times than reported gamma (γ)-rays [225]. Following the same line, Fakharan et al. applied a YAG-pulsed laser to produce rGO in formic acid for OPVs with a PCE of 4.02% [226]. They also highlighted the solvent’s role in manufacturing devices to achieve an rGO with superior physical and electrical features. Unlike the traditional chemical-reduction methods, pulsed laser or electron-beam allowed the reduction of graphene over the in-situ formation of reducing species selectively. Dericiler et al. reported graphene nanosheets prepared from the electrochemical exfoliation of graphene powder followed by dispersion in DMF solvent [227]. They used the graphene nanosheets suspension as an additive to PEDOT:PSS HTLs to enhance the stability and charge mobility in the P3HT:PC_60_BM, achieving 66% enhancement in the PCE compared with the reference cell based on pure PEDOT:PSS HTLs.

**Figure 11:**
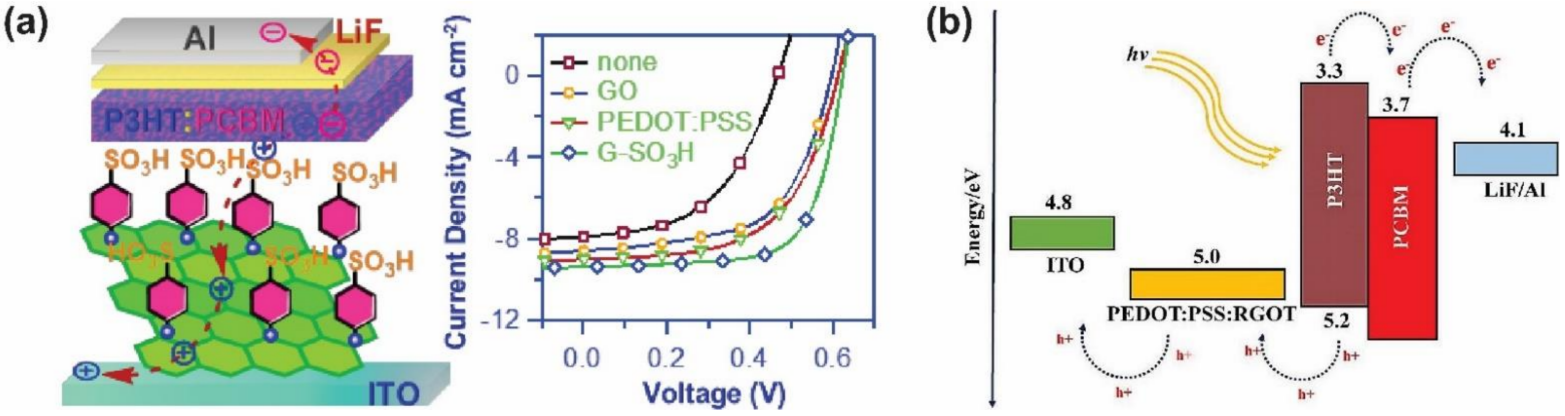
(**a**) J-V curve of the OSCs with G–SO_3_H films as HTL. Adapted with permission from [230]. Copyright 2018, American Chemical Society. (**b**) Energy level diagram of an OSC with RGOT-modified HTL. Adapted with permission from [237]. Copyright 2020, John Wiley and Sons.

The application of UVO irradiation has shown excellent efficiency in reducing GO in large-scale OSCs manufacturing. Xia et al. [228] and Rafique et al. [229] exposed GO to UVO treatment, resulting in optimized performance in P3HT:PC_71_BM and PCDTBT:PC_71_BM blend systems. UVO oxidizes the surface of GO and removes CO_2_ molecules, leaving a uniform, smoothed, and conductive film. Ultraviolet irradiation was controlled to remove only C-O bonds from the GO surface. UVO-treated GO films allowed for exceeding the value of FF and J_sc_ obtained from PEDOT: PSS. Taking advantage of graphene’s chemical structure, the functionalization is very promising for obtaining desirable properties in HTLs, such as high hole mobility, charge collection, transparency, and stability, among others. Zhao et al. fabricated highly stable P3HT:PC_71_BM-based OSCs with a PCE of 3.56% by forming covalent bonds between graphene and sulfanilic acid through C-N linkers [230]. The covalent functionalization increased the WF that enhance the interface’s charge transport and the overall photovoltaic characteristics (see Figure 11a). Ali et al. confirmed the potential for tuning the bandgap and electrical properties when reduced and sulfonated GO films were applied as HTLs for a wide range of donor-acceptor systems [231]. Other approaches like non-covalent phosphorylation and fluorination have been remarkably effective in enhancing the charge collection and transport via inducing low ohmic contact [232,233]. The presence of the phosphate ester or fluor in the surface of GO increased the WF of ITO/GO and tuned the HOMO level of the donor by the p-doping effect. Fluorinated GO (F_5_-GO) was reported to work as an interlayer between ITO and PEDOT:PSS [234]. This material improved hole transport, resulting in a low R_s_ of 2 Ωcm^2^ and a PCE of 7.67% for PTB7:PC_71_BM-based OSCs. Park et al. reported an orthogonal printable HTL by spray casting a highly stable dispersion of fluorine-functionalized reduced graphene oxide (FrGO) [235]. By decreasing the sheet size to 0.3 µm, the PCE increased to 9.27 and 9.02% for PTB7-Th:EH-IDTBR and PTB7-Th:PC_71_BM-based OSCs, respectively. This improvement was attributed to the hole-transport efficiency, decreased leakage current, and higher conductivity of the FrGO layers. Zhen et al. reported graphene-MoS_2_ hybrid thin films via liquid-phase graphene exfoliation, improving the charge transportation as an interlayer to achieve a PCE 9.5% [236]. This interlayer increased the device stability by retaining 93% of the initial PCE after 1000 h at room temperature. Shoyiga et al. reported reduced graphene oxide-anatase titania (RGOT) nanocomposites by hydrothermal synthesis [237]. RGOT HTL is an efficient charge-transport channel whose higher conductivity and exciton dissociation efficiency decreased the rates of electron-hole recombination (see Figure 11b), resulting in a high J_sc_, low R_s_, and, thus, improved photovoltaic performance.

**Figure 12:**
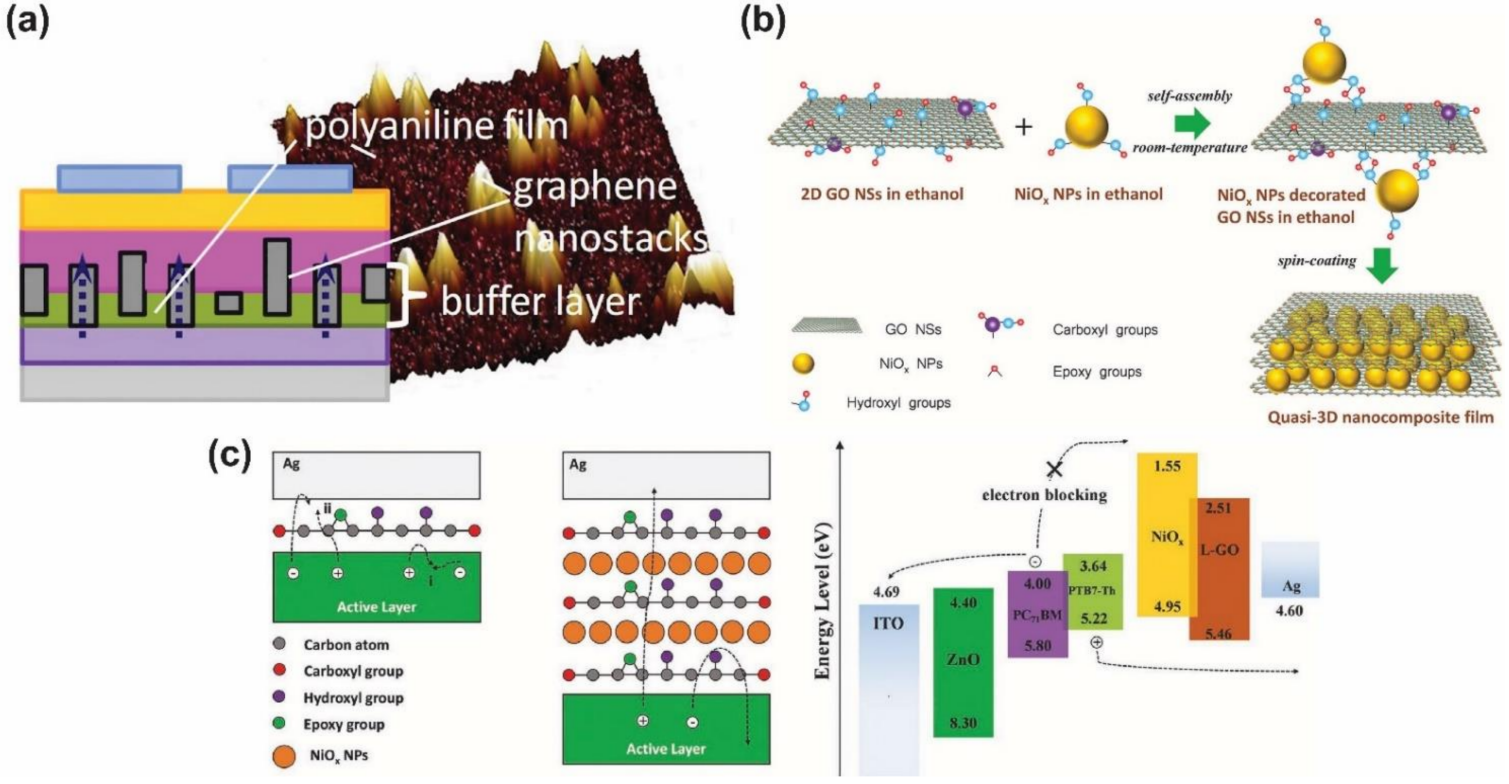
(**a**) Topography AFM-images of G/PANI–PAMPSA nanocomposite layer. Adapted with permission from [243]. Copyright 2018, Elsevier. (**b**) Schematic representation of the preparation at room temperature of self-assembled quasi-3D GO:NiO_x_ nanocomposite and (**c**) hole extraction properties and dynamics at the interface. Adapted with permission from [245]. Copyright 2018, John Wiley and Sons.

Lee et al. improved the performance of rGO by chemical doping with tetrafluorotetracyanoquino-dimethane (F_4_TCNQ) [238]. The *p*-doping of rGO with F_4_TCNQ increased the WF by 0.2 eV and the conductivity by inducing charge transfer between the F_4_TCNQ and the graphene layer. F_4_TCNQ enhanced the interchain interaction and crystallization of the P3HT film to improve the hole mobility from the active layer to the anode. Lee et al. [239] and Sun et al. [240] reported that GO modified with alkali chlorides such as AuCl_3_ or CuCl_2_ dopants in a conventional architecture exhibited an average PCE of 3.77% and 7.68%, respectively. The AuCl_3_-doped graphene increased the electrical conductivity (~2.0 × 10^5^ Sm^−1^) compared with the reported fullerenol-rGO layer (1 × 10^−2^ Scm^−1^). GO:CuCl_2_ layers formed a uniform and continuous film. Although the efficiency achieved by the dopants is even comparable to that of the control devices with PEDOT:PSS, the stability was superior. Graphene-based derivatives (GBD) do not corrode the metal substrate as PEDOT:PSS, to leading the development of OSCs’ efficient performance by controlling the properties and deposition conditions of GBD as reported by Capasso et al. [241]. Sarkar et al. embedded Au NPs into GO for increasing the light trapping in the active layer [242]. The exerted plasmonic effect and the plasmon-exciton interaction of NPs increased the light harvested by the active layer, resulting in enhanced J_sc_ and PCE. Besides, the enhanced conductivity of GO helped to reduce the leakage current, thereby improving the photogenerated current, R_s_, and FF of the device. A composite of 1 wt% of graphene nanosheet and water-dispersible polyaniline-poly(2-acrylamido-2-methyl-1-propanesulfonic acid) complex was used as HTL in OSCs [243]. The graphene nanostacks (GN) from the composite penetrated the BHJ of the OSC and facilitated the charge transport by forming additional pathways (see Figure 12a). The electric field generated from the edges of the GN increased the exciton dissociation. As a result, the composite performance raised the PCE from 2.12% (PANI) to 2.92% (G-PANI) in the P3HT:PC_70_BM cells. Aatif et al. also reported the surface morphology’s planarization after applying GO/molybdenum composite, resulting in a PCE of 5.1% with the PCDTBT:PC_71_BM-based OSCs [244]. Quasi-3D GO:NiO_x_ nanocomposites are potential *p*-type HTLs in ITO/ZnO/PTB7-Th:PC_71_BM/HTL/Ag architectures [245]. Using the solvothermal method, NiO_x_ NPs interacted with the low oxidized form of GO by hydrogen bonds to form the quasi 3-D arrangement (see Figure 12b). The high performance of these nanocomposite HTLs is due to the enhanced vertical conductivity with low recombination rates and enhanced electron-blocking ability by the small conduction band of NiO_x_ NPs (1.55 eV) (see Figure 12c). The metallic nature of NiO_x_ NPs improved the stability by retaining half of the initial PCE (12.3%) in environmental conditions. Dang et al. reported a solution-processed hybrid graphene-MoO_3_ (G-MoO_3_), via the hydrothermal method, to apply as HTL in OSCs [246]. The G-MoO_3_ exhibited higher transparency in the visible region compared with the thermal-evaporated MoO_3_. Moreover, the low injection barrier (0.2 eV) and the higher hole mobility in G-MoO_3_ (4.16 × 10^−5^ cm^2^V^−1^s^−1^) than in MoO_3_ (1.25 × 10^−5^ cm^2^V^−1^s^−1^) were beneficial to achieve a PCE of 7.07%. The rGO and perylene derivative 3,4,9,10-perylenetetracarboxylic dianhydride (PTCDA) nanohybrid HTL showed an increased cell performance up 4.70% in PBDTTT-CT:PC_71_BM-based cells [247]. The rGO:PTCDA nanohybrid HTL formed permanent dipoles by the PTCDA_rGO bond formation, increasing the hole extraction, electrical conductivity, and tuning the WF.

#### 4.3.2. Other Nanocarbons

**Figure 13:**
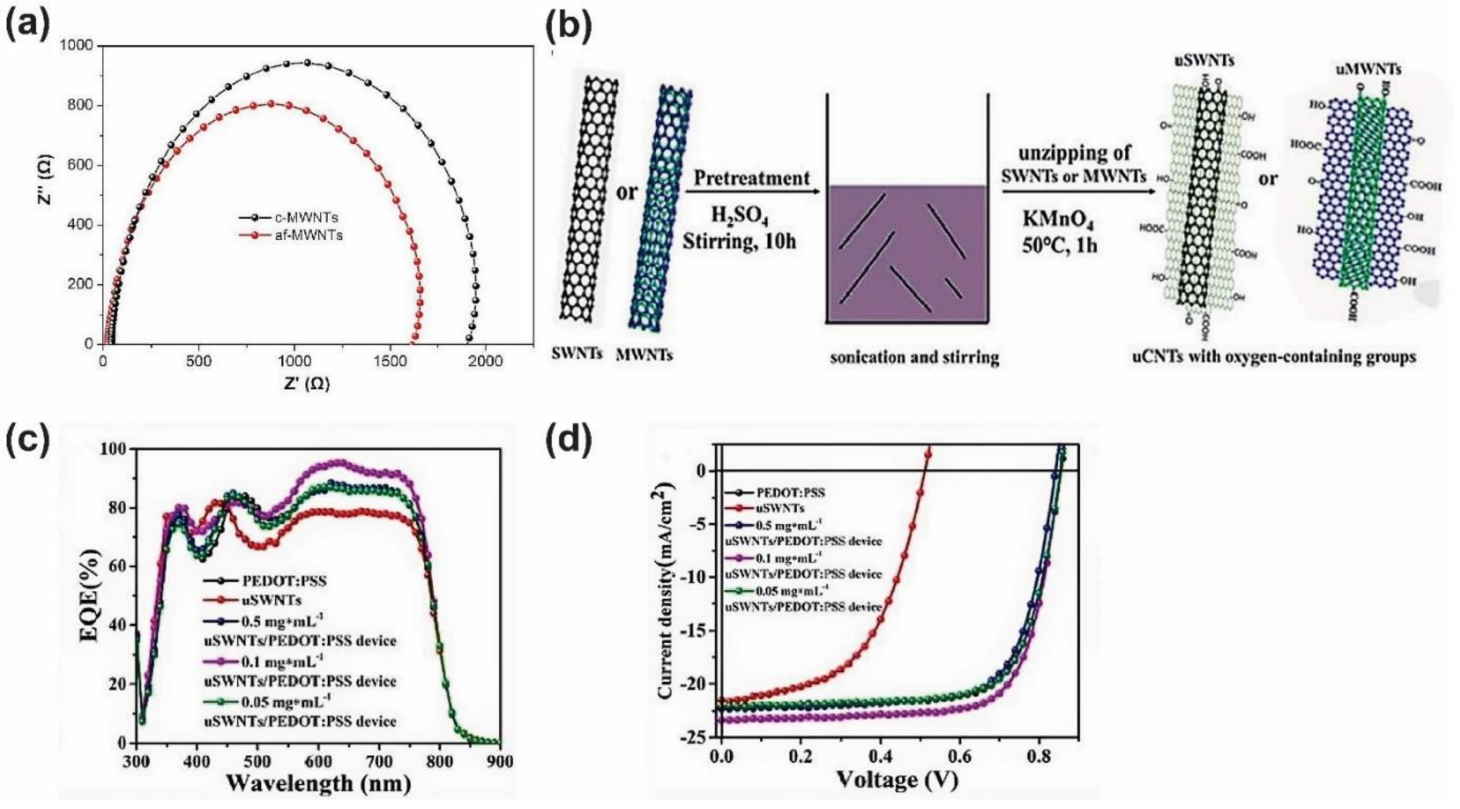
(**a**) Impedance spectra of OSCs with c-MWNTs and af-MWNTs HTLs. Adapted with permission from [252]. Copyright 2019, Elsevier. (**b**) Schematic representation of u-SWNTs and u-MWNTs synthesis, (**c**) EQE of u-SWNTs, SWNTs, and PEDOT:PSS, and (**d**) J-V curves of the OSCs with u-SWNTs HTLs. Adapted with permission from [254]. Copyright 2020, Royal Society of Chemistry.

The production efficiency of graphene quantum dots (GQDs) in the photovoltaic field has been limited by the expensive manufacturing methods, materials availability, and the time-consuming manufacturing [248,249]. However, the development of green and low-cost methods, such as the synthesis of GQDs from carbon fibers by acid treatment and chemical exfoliation or doping with nitrogen, has boosted its potential application in the fabrication of large-area OSCs [250]. Hoang et al. succeeded in the green synthesis of GQDs from graphene using the microwave-assisted hydrothermal method for 10 min [251]. An enhancement of 44% in PCE was achieved by doping the active layer with 2 mg of GQDs. The GQDs filled the interstitial positions between P3HT and PC_60_BM to increase the charge transport of holes and electrons and the photocurrent generation. Zhang et al. reported amino-functionalized multi-walled carbon nanotubes (a-MWNTs), via hydrothermal synthesis, as HTLs in conventional OSCs with the configuration ITO/a-MWNTs/PCDTBT/PC_71_BM/LiF/Al [252]. Compared with the carboxylic acids, the amino functionalization reduced the defects and the resistivity of a-MWNTs (see Figure 13a). The a-MWNTs enhanced the device’s charge mobility, collection, and performance by 6.9%. Single-walled carbon nanotubes (SWCNTs) are promising *p*-type transparent conductors owing to their superior hole mobility, conductivity, and facile tuning of the WF by doping method [253]. A highly-conductive composite of unzipped single-walled carbon nanotubes (u-SWNTs) and PEDOT:PSS was synthesized by a facile solution processing method as reported by Zhang et al. (see Figure 13b) [254]. The hybrid PEDOT:PSS doped with u-SWNTs decreased the surface roughness. Oxygen-containing groups of u-SWNTs improved the compatibility between u-SWNTs and PEDOT:PSS to block electrons and increase the hole transport. Using 0.1 mg mL^−1^ of u-SWNTs, the conductivity of the uSWNTs/PEDOT:PSS increased to 2.08 Scm^−1^. The R_s_ was insensitive to the layer thickness, resulting in improved charge-carrier transport through the gap of u-SWNTs (see Figure 13c). Thus, PBDB-T-2F:IT-4F devices with u-SWNTs/PEDOT:PSS HTLs exhibited an enhancement in the PCE from 13.72% to 14.60% (see Figure 13d).

Table 1 enlists a series of HTLs reviewed up to this point. The anode configuration with its work function, deposition technique for the HTL, active layer composition, and the OSCs performance parameter such as V_OC_, J_SC_, FF, and PCE are provided as well as the reference where the information is available.

### 4.4. Conducting Polymers and Their Composites

#### 4.4.1. PEDOT

**Figure 14:**
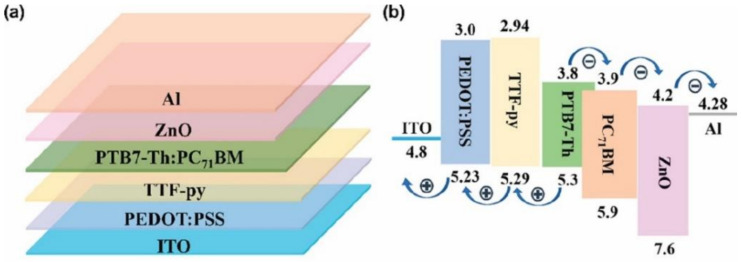
(**a**) Schematic illustration of device structure of ITO/PEDOT.PSS/TTF-py/PTB7-Th:PC_71_BM/ZnO/Al and (**b**) energy level diagram of an OSC with the TTF-py modified PEDOT:PSS as HTL. Adapted with permission from [268]. Copyright 2019, American Chemical Society.

PEDOT:PSS is the most common conducting polymer used as hole-transporting material in OSCs due to its easy solution processing, suitable WF around 5.1 eV, high conductivity, good transparency, good mechanical properties, and adapted wettability on the BHJ layer [255]. For instance, a patterning interfacial PEDOT:PSS layer formed by a nanoimprinting technique using poly(dimethylsiloxane) (PDMS) stamp was employed on OSCs based on poly(3-hexylthiophene):phenyl-C61-butyric acid methyl ester (P3HT:PCBM), showing an increased PCE of 1.53% [256]. PEDOT:PSS was incorporated on an inverted OSC based on P3HT:O-IDTBR with an evaporated Ag back electrode, showing an enhanced device performance [257]. The incorporation of PEDOT:PSS into P3HTN:PEG-C_60_ based OSCs increased the V_oc_ to 1.3 V, attributed to the large collection barrier [258]. PEDOT:PSS has strong acidic nature due to the polystyrene sulfonate (PSS, pH~2), which deteriorates the anode material and the photoactive layer, affecting the performance and stability of the device. Besides, this polyelectrolyte has high affinity for environmental water (hygroscopic), making it necessary to encapsulate the OSC’s before the durability test. Humidity is a major problem in this type of device. Accordingly, different modifications to the PEDOT:PSS layer have been developed to overcome these issues. Some post-treatments to the PEDOT:PSS layer have been tested using solvents, surfactants, and by exchange of PSS with less acidic dopants as well as the addition of small molecules. These modifications aim to reach a uniform morphology, increase the interface contact, and produce a neutral pH hole-transporting polymer to improve the stability and cell performance. The use of a layer composed of PEDOT and grafted sulfonated-acetone-formaldehyde lignin (GSL) instead of PSS resulted in a better photovoltaic performance than conventional PEDOT:PSS [259]. GSL is a less acidic copolymer of lignin. A homogeneous surface of the PEDOT:GSL HTL in a PTB7-Th:PC_71_BM/poly[(9,9bis(3′-(*N*,*N*-dimethylamino)propyl)-2,7-fluorene)-alt-2,7-(9,9dioctylfluorene)] (PFN)-based OSC resulted in a PCE of 8.47%. PEDOT:PSS treatment with solvents as isopropanol (IPA) also shows a better performance, mainly due to more uniform morphology, increased J_SC_, and improved cell-light absorption [260]. 2-Methoxyethanol (EGME) and dimethyl sulfoxide (DMSO) solvents were added to a PEDOT:PSS solution [261]. The conductivity after doping was about seven times higher and the OSCs based on P3HT:PCBM improved the PCE from 2.8% to 3.9% owing to increased J_sc_ 16.5 mA cm^−2^ and FF of 38.0%. Another approach includes the addition of commercial surfactants such as Zonyl FS-31, which improves the wettability of the interface between the hydrophobic photoactive layer and the PEDOT:PSS HTL [262]. The fluorination of PEDOT:PSS HTL by fluorinated molecules showed an increased device efficiency [263]. On the other hand, a PSS-free, stable PEDOT HTL was obtained by using solid-state polymerization resulting in robust, stable, and solution-processable OSCs based on PCDTBT:PC_71_BM [264]. Water-soluble polyelectrolyte poly(4-(2,3-dihydrothieno[3,4-b][1,4]dioxin-2-yl-methoxy)-1-butanesulfonic acid) (PEDOT-S), which shows the same PEDOT backbone containing an ethoxyalkylsulfonate branch, showed better performance than conventional PEDOT:PSS layers [265]. PEDOT-sulfonated polyelectrolyte complexes were also tested as an anode buffer layer [266]. Different commercial grades of PEDOT:PSS and additive solvent EG were used to form a hybrid PEDOT:PSS (PH 1000:Al 4083) layer tested as an HTL and anode electrode for inverted OSCs based on P3HT:PCBM [267]. An OSCs based on PTB7-Th:PC_71_BM was built using a hole-transport double layer made of pyridine-based tetrathiafulvalene derivative (TTF-py) on PEDOT:PSS [268]. This modification resulted in an increased short circuit current (J_sc_) of 17.19 mA cm^−2^ and a PCE of 9.37%. The anode configuration showed a WF of 5.28 eV for the TTF-py layer, resulting in a closer valence band toward the donor material (see Figure 14). PEDOT:PSS/TTF-py had better wettability and enhanced hole mobility, resulting in charge-loss reduction and charge-recombination suppression. Furthermore, TTF-py’s molecular structure allowed molecular π-π stacking and formed an orderly molecular arrangement for hole transfer. TTF-py modification also improved the device stability, retaining 96% of the initial PCE after storing for 28 days by the suppression PEDOT:PSS permeation.

**Figure 15:**
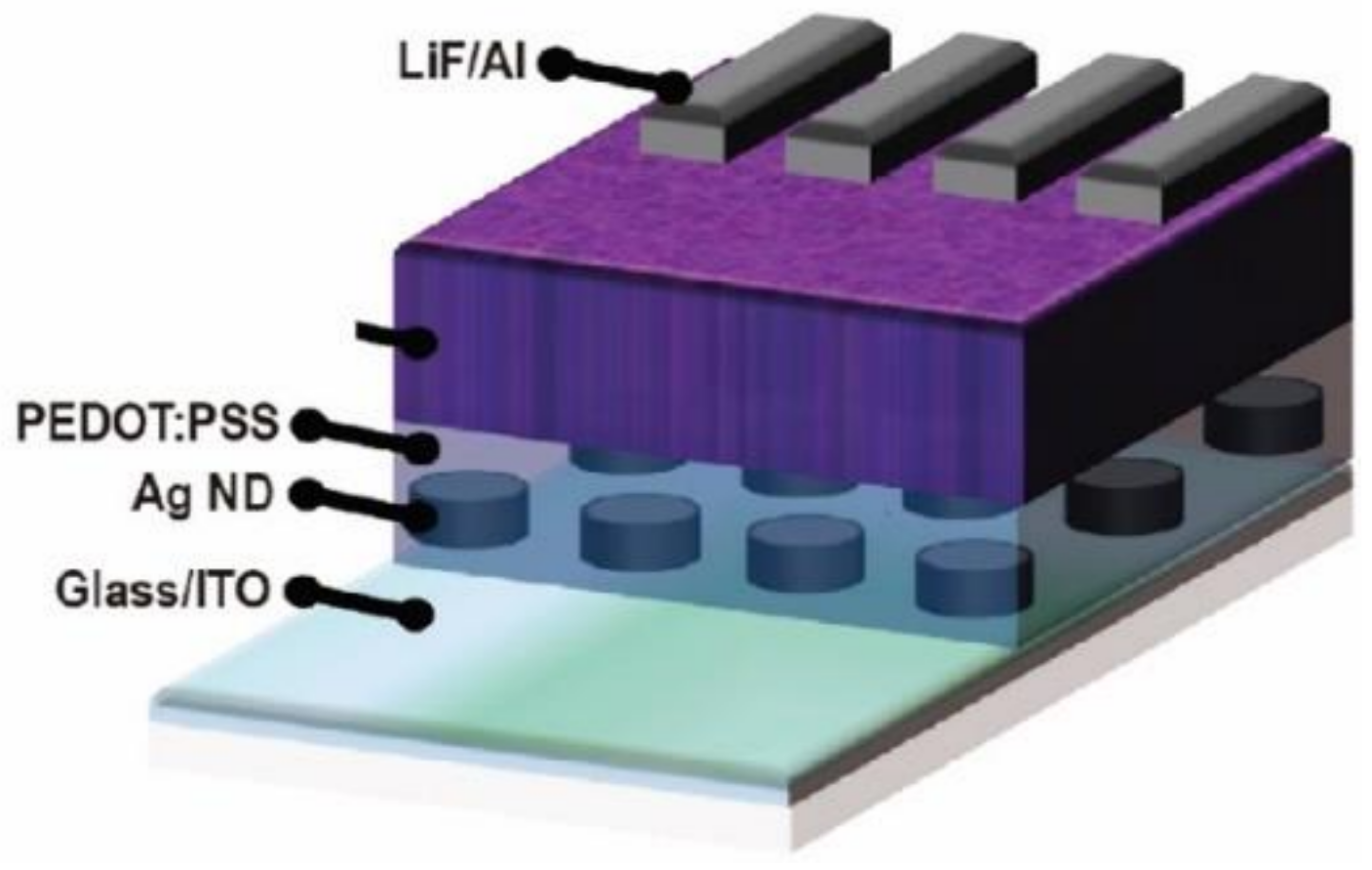
Schematic representation of an OPV device with Ag NDs. Adapted with permission from [291]. Copyright 2016, American Chemical Society.

Other modifications on PEDOT:PSS have introduced an inorganic transition metal salt, such as nickel formate dihydrate (NFD), to tune the surface free energy (γ_s_) and control the molecular orientation in the BHJ [269]. An enhanced PCE of 10.76% was achieved for the PM6:PC_71_BM-based OSCs. The NFD:PEDOT:PSS HTL had a WF of 5.01 eV that well-matched the donor material and an increased γ_s_ of 68.96 mN m^−1^, which led to increased FF and J_sc_. Polymeric donor material PM6 preferred a face-on molecular orientation. Enhanced molecular stacking was promoted with an increased γ_s_ of PEDOT:PSS induced by NFD. This modification improved the molecular orientation along the charge-transport direction; thus, carrier mobility was enhanced, and the charge recombination was suppressed. The modified HTL was also tested in non-fullerene OSCs based on PM6: IT-4F, obtaining an enhanced PCE of 14.08% with FF of 78.75%. Oxoammonium salts (TEMPO^+^ Br^-^, 2,2,6,6-tetramethylpiperidine-1-oxoammonium) were tested as a p-type dopant of PEDOT:PSS layers, resulting in an enhanced PCE of 16.1% in OSCs based on PM6:Y6 [270]. PEDOT:PSS was further oxidized by oxoammonium salt, improving the doping level of PEDOT:PSS. Doped PEDOT:PSS (TEMPO^+^ Br^-^) possess higher conductivity and better energy alignment. Metallo phthalocyanines (PC) such as vapor-deposited vanadylphthalocyanine (VoPC), NiPC, and SnPC were tested as buffer layers with PEDOT:PSS, enhancing the efficiency of P3HT:PCBM-based OSCs [271]. PEDOT:PSS:In_2_S_3_ was also employed as HTL material for OSCs based on PBDB-T:ITIC and PM6:Y6; these showed an enhanced PCE of 11.22% and 15.89%, respectively [272]. Improved device performance was observed because of increased J_sc_ and FF, and reduced R_s_ with bimolecular recombination suppression due to partial removal of PSS from the surface. PEDOT also suffered a benzoic-quinoid transition (coil-linear structure) which delocalized charge carriers, enhancing the layer conductivity. Furthermore, device performance stability showed a retained 36% PCE for modified HTLs after 48 h compared with a non-modified PEDOT:PSS HTL, which showed a retained 10% PCE. OSCs based on ITO/PEDOT:PSS-Dopamine (DA)/PM6:Y6/poly[[2,7-bis(2-ethylhexyl)-1,2,3,6,7,8-hexahydro-1,3,6,8-tetraoxobenzo[lmn] [3,8]phenanthroline-4,9-diyl]-2,5-thiophenediyl[9,9-bis[3′((*N*,*N*-dimethyl)-*N*-ethylammonium)]propyl]-9H-fluorene-2,7-diyl]-2,5-thiophenediyl] (PNDIT-F3N)/Ag showed an increased PCE from 16.01% to 16.55% [273]. The DA-doped PEDOT:PSS layer showed an enhanced conductivity ascribed to (i) a more regular stack by the enhanced intermolecular packing of DA:PSS, (ii) an increased WF of 5.14 eV compatible with HOMO level of PM6 donor polymer, and (iii) enhanced film uniformity. PEDOT:PSS-DA was also tested for devices based on different active layers such as PBDB-T:ITIC, PM6: IDIC, and P3HT:PCBM, resulting in improved performances as well. PEDOT:PSS was also used together with various polymers as HTLs, such as nanoimprinted poly(methylmethacrylate) (PMMA) [274], and conjugated polyelectrolytes (CPEs), e.g., poly[(9,9-bis(4-sulfonatobutyl sodium) fluorene-alt-phenylene)-ran-(4,7-di-2-thienyl-2,1,3-benzothiadiazole-alt-phenylene)] (PSFP-DTBTP), that resulted in a PCE improvement of 13% for PCDTBT:PC_71_BM-based OSCs [275]. Microporous polymer networks are a class of conjugated material that shows high specific surface areas and porosity with potential application in various fields including organic photovoltaics [276,277,278,279,280,281,282,283]. For instance, a porous organic polymer, poly(carbazolyl triphenylethylene) derivative (PTPCz), obtained by electropolymerization was used in the HTL PEDOT:PSS/PTPCz for OSCs based on PTB7:PC_71_BM, resulting in an smooth surface morphology, increased WF of 5.23 eV, J_sc_, and FF, reduced R_s_, and increased R_sh_, reaching an improved PCE of 8.54% [284]. Electropolymerized polytriphenylcarbazole fluoranthene (p-TPCF) and PEDOT:PSS were used for OSCs based on PTB7-Th:PC_71_BM, obtaining enhanced J_sc_, FF, V_oc_, and a PCE of 8.99% [285]. Modification of PEDOT:PSS with a neutral conjugated polymer electrolyte poly[9,9-bis(4′-sulfonatobutyl)fluorene-*alt*-thiophene] (PFT-D) composite layer improved the device performance (PCE from 7.8% to 8.2%) and the half-lives of PTB7-Th:PC_71_BM-based OSCs [286]. PFT-D molecular dipole screened the attraction between PEDOT and PSS chains; additionally, the –SO_3_^−^ ions of PFT-D act as a conjugate base of PSS, improving current generation. Poly(3-hexylthiophene)-*b*-poly(p-styrenesulfonate) (P3HT_50_-b-PSS_23_) block polymers were incorporated between HTL PEDOT:PSS and the active-layer P3HT:PCBM [287]. The OSCs with P3HT-b-PSS interfacial layer improved PCE by 12% due to increased V_oc_ and FF that compensate for the decreased J_sc_ caused by the blocked light irradiance to the P3HT. The energy level matching was improved. HOMO level of P3HT-b-PSS (−4.68 eV) was higher than P3HT of active layer, which facilitates the hole transport. In addition, P3HT-b-PSS film had a smoother surface than PEDOT:PSS, enhancing the interfacial contact and thus improving the FF of the device. Modification of the commonly used PEDOT:PSS with metallic NPs contributes with some features such as an enhanced localized field and light scattering by the localized surface-plasmon resonance (LSPR) that improves the absorption of the active layer [288]. NPs also assist in the charge transport at the interface. NPs are synthesized by different methods such as chemical reduction, the polyol method, and ultrasonochemical synthesis [289]. Hao et al. reported mixed AuNPs (rod, bone-like, cube and spheres shape) doped in PEDOT:PSS HTLs in OSCs based on PTB7:PC_71_BM [290]. The addition of mixed AuNPs generated wide absorption spectra covering from the visible to the near-infrared region and induced an increase of enhancement of internal field in the active layer, resulting in improved absorption and enhanced device performance up to 9.26%. AuNPs also contributed to decrease the bulk resistance of PEDOT:PSS. Periodic Ag nanodot (Ag ND) arrays were fabricated by laser-interference lithography (LIL) between ITO and PEDOT:PSS layers in OSCs based on PTB7:PC_70_BM (see Figure 15) [291]. This HTL showed increased J_sc_ of 23.26 mA/cm^2^, enhanced EQE induced by the plasmonic and light-scattering effect, and improved PCE of 10.11%. LSPR band matched optimally with the absorption of the photoactive layer, increasing its light-absorption.

**Figure 16:**
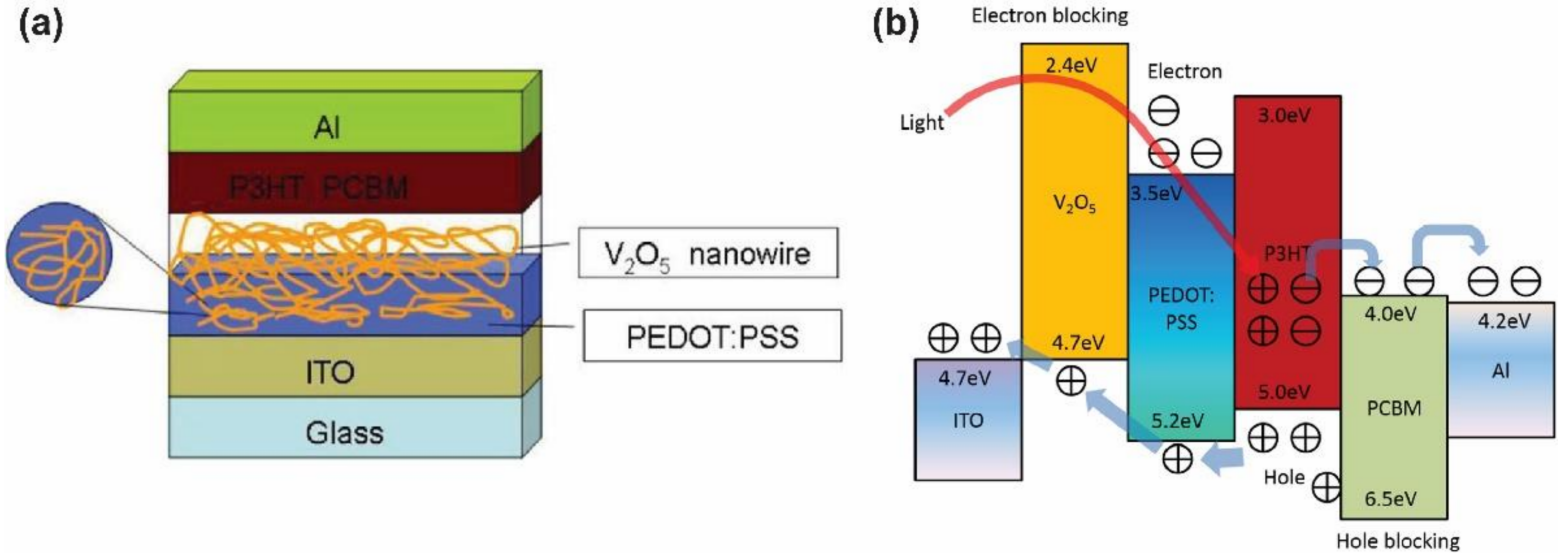
(**a**) Schematic representation of polymer photovoltaic cell based on PEDOT:PSS/V_2_O_5_ HTL and (**b**) energy level alignment for the cell components. Adapted with permission from [308]. Copyright 2016, Elsevier.

AuNPs and AgNPs blended with PEDOT:PSS were used as HTLs in OSCs [292]. Both PEDOT:PSS:NPs showed an enhanced device performance in comparison with pristine PEDOT:PSS. An optimized PCE of 5.65% was obtained for PEDOT:PSS:AuNPs HTLs in rrP3HT:PC_71_BM-based OSCs. Better device performances were obtained with NPs because of the surface-plasmon effect (at the visible region for AuNPs) that increased the photoabsorption length and the trapping of scattering and incident light. Segmented silver nanowires (AgNWs) were incorporated in PEDOT:PSS HTLs in OSCs with configuration ITO/PEDOT:PSS:AgNWs/P3HT:PC_61_BM/ZnO/Al [289]. These OSCs exhibited enhanced device performance with a PCE of 3.3%, increased J_sc_ and FF due to LSPR, and optical-scattering properties from the AgNWs. Sah et al. reported the fabrication of OSCs based on PTB7-Th:PC_71_BM with bimetallic Ag-Au-Ag nanorods (NRs) in PEDOT:PSS HTLs [293]. These devices showed improved performances up to 7.36% owing to an increased FF and J_sc_. The enhancement was ascribed to an improved charge transport, broad absorption region covering visible to the near-infrared region, light-scattering-induced absorption enhancement, electric field enhancement, and improved EQE by the LSPR effect. Incorporation of copper, a cheaper and more abundant metal, as Cu-Au NPs in PEDOT:PSS for OSC based on P3HT:PC_61_BM and PTB7-Th:PC_71_BM showed improved PCEs of 3.63% and 8.48%, respectively [294]. Cu-AuNPs:PEDOT:PSS presented an absorption enhancement by LSPR and light-scattering effect. The improved PCE in the device was attributed to an increased J_sc_, higher hole mobility, and reduced R_s_. However, the FF decreased compared with pristine PEDOT:PSS HTLs, mainly due to an induced charge recombination by the NP doping. Adedeji et al. employed copper sulfide NPs in PEDOT:PSS HTLs to fabricate OSCs based on P3HT:PC_61_BM [295]. These devices showed an enhanced PCE of 4.51% (an increase of 115% over the pristine PEDOT:PSS HTLs) and good stability, retaining up to 40% of their initial PCEs after 48 h. CuNPs exhibited surface-plasmon resonance absorption near-infrared region and induced an electric field beneficial to exciton dissociation and photon harvesting. The incorporation of nickel sulphide NPs in PEDOT:PSS layers exhibited an enhanced device performance of 6.03% in OSC based on P3HT:PC_61_BM [288]. An improved photogenerated current was attributed to the effective trapping of light through scattering and improved charge collection. OSCs based on NiS NPs:PEDOT:PSS HTLs showed reduced R_s_, indicative of an improved conductivity at the interface, and improved optical transparency enhancing the internal quantum efficiency. Furthermore, the device showed higher hole mobility, and thus reduced carrier recombination and enhanced charge transport. PCE enhancement was ascribed to the LSPR absorption (in the visible and infrared region) and light-scattering process. ZnSTe quantum dots (QDs) incorporated into PEDOT:PSS HTL showed an improved device performance in OSC based on P3HT:PC_71_BM due to improved mobility and enhanced light absorption, attributed to surface-plasmon resonance [296]. Zhang et al. reported OSCs based on PTB7-Th:PC_71_BM and PM6:IT-4F with high PCEs of 9.11% and 12.81%, respectively, by the insertion of black phosphorous quantum dots (BPQDs) on PEDOT:PSS [297]. BPQDs is a 2D p-type semiconductor, which, inside the device, formed a cascade band structure between the anode and the active layer. The valance band of BPQDs (−4.92 eV) was higher than the valance band of donor polymers PTB7-Th (−5.24 eV) and PM6 (−5.5 eV), providing enough driving force for the hole injection from the active layer to the BPQDs layer. The increased efficiency in devices with BPQDs interfacial layer was attributed to an increased J_sc_ and FF due to excellent hole mobility in BPQDs and better energy alignment in the device, indicating improved charge extraction and exciton dissociation. Other metal NPs incorporated in PEDOT:PSS involve Al micro-stars [298], Al NPs [299], and Au NPs, gold nanorods (Au NRs) [300], and Au QDs [301]. Up-conversion NP process converts low-energy photons into high-energy photons (in the absorption region of organic polymers) to enhance the optical-to-electrical conversion performance [302]. Mei et al. incorporated sodium yttrium fluoride (β-NaYF_4_):Er^3+^, Yb^3+^ up-conversion NPs into PEDOT:PSS HTLs in OSCs based on P3HT:PC_61_BM, resulting in enhanced J_sc_ and PCE of 3.02% [303]. These results were ascribed to light scattering and photoluminescence (PL) emission from up-conversion NPs. Silver-zinc bimetallic NPs were incorporated between the PEDOT:PSS HTL and the photoactive layer of P3HT:PCBM [304]. OSCs exhibited an improved PCE of 3.6%, which was 90% higher than the reference device. This effect was attributed to LSPR of the Ag:Zn NPs, which enhanced the optical absorption and charge-carrier collection. Another modification to PEDOT:PSS was reported by Michalska et al. using wet ultra-sonochemical synthesized titanium dioxide TiO_2_ anatase decorated with Ag NPs [305]. TiO_2_/Ag solution was added to PEDOT:PSS HTLs showing an improved PCE of 2.07%. Au NPs on PEDOT:PSS in vacuum-free OSCs improved the device performance resulting from the increased J_sc_ [306]. The absorption of the active layer and the device PCE were enhanced, especially by Au nanorods’ presence. Many studies have been focused on improving the performance of PEDOT:PSS HTL by addressing the acidic and hygroscopic nature of PEDOT:PSS that affects the stability and efficiency of the photovoltaic devices. Incorporating metal oxides (MO) can enhance the stability, efficiency, and electron-blocking properties of the HTL. Among these MOs that have been incorporated in PEDOT:PSS are vanadium oxides (V_2_O_5_), sol-gel synthesized VO_x_, continuous-spray pyrolyzed-synthesized molybdenum oxide (MoO_3_), and tungsten oxide (WO) [307]. Spin-coated V_2_O_5_ NWs prepared by the hydrothermal method on PEDOT:PSS HTL (see Figure 16a) showed improved V_oc_ and FF in OSCs based on P3HT:PCBM [308]. The PCE improved a 15.58% in comparison with the pristine PEDOT:PSS reference cell. The LUMO level of V_2_O_5_ (2.4 eV) is higher than P3HT LUMO, and thus better electron blocking properties than pristine PEDOT:PSS are expected (see Figure 16b). Modified HTL had increased incident light paths by reflection and refraction caused by V_2_O_5_ NWs.

**Figure 17:**
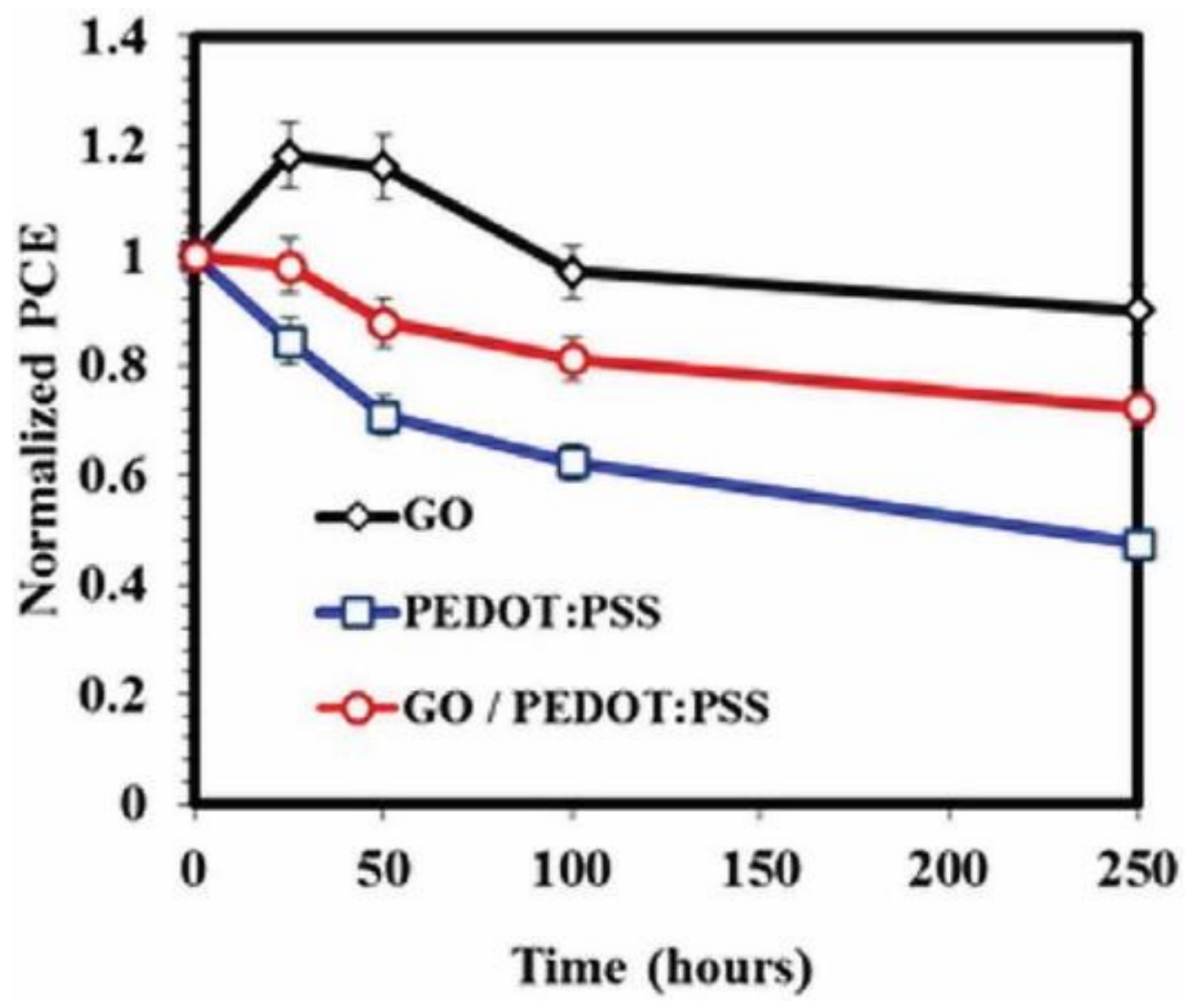
Stability test of various OSCs over 250 h. Adapted with permission from [328].

Li et al. reported a PCE of 9.44% for OSCs with V_2_O_5_:PEDOT:PSS as HTL due to an enhanced J_sc_ and FF, smaller R_s_, and larger R_sh_ [309]. The incorporation of V_2_O_5_ offered an effective path for exciton extraction and suppressed charge recombination, reflected by a larger hole mobility and a higher conductivity. The composite HTL surface was uniform and smooth, related to V_2_O_5_ NPs filling the pinholes in PEDOT:PSS. Furthermore, better wetting and physical contact were obtained between the photoactive layer and the HTL as well as enhanced crystallinity of the active layer. Molybdenum oxide (MoO_x_) NPs/PEDOT:PSS HTLs were blade coated in inverted OSCs based on PTB7-Th:PC_60_BM, resulting in an increased FF and enhanced PCE of 7.4% [310]. The modification of PEDOT:PSS with MoO_3_ mitigated the degradation of non-fullerene OSCs based on PM6:IT-4F by suppressing the interfacial reaction between PEDOT:PSS and IT-4F [311]. MoO_3_-PEDOT:PSS hybrid HTL improved the device’s operational stability, which was five times longer than reference devices. The hybrid HTL also improved the hole mobility favoring the charge extraction. Zinc oxide-doped single-carbon nanotubes (CNT) were incorporated in PEDOT:PSS as an anode buffer layer (ZnO:CNT/PEDOT:PSS), showing excellent transmittance and a smooth morphology [312]. P3HT:PCBM-based OSCs with an HTL containing 2.5% ZnO:CNT showed an improved PCE of 4.1%, enhanced J_sc_ and FF, and reduced R_s_. CNT provided surface homogeneity, and ZnO prevented humidity uptake. The device parameters decreased at a slower rate than PEDOT:PSS devices under a nitrogen environment. Zheng et al. fabricated fullerene-free OSCs with tungsten oxide WO_x_ NPs in PEDOT:PSS as HTL [313]. The system architecture ITO/WO_x_:PEDOT:PSS/PM6:IT-4F/PFN-Br/Al achieved a high FF of 80.79% and enhanced PCE of 14.57%. A more balanced hole and electron mobility was obtained for WO_x_:PEDOT:PSS based of BHJ OSCs measured as a ratio $\mu_{e}/\mu_{h}\;$ of 0.88, which contributed to increase the FF. The longer lifetime of carriers and faster extract time of WO_x_:PEDOT:PSS also benefited the device parameters. WO_3_/PEDOT:PSS bilayer was used as HTL in inverted SMD2: ITIC-Th-based OSCs [314]. An optimized cell achieved a high PCE of 10.3%, with enhanced J_sc_, V_oc_, and FF. The WO_3_/PEDOT:PSS device presented increased R_sh_ and decreased R_s_ by a well-matched energy level alignment, high hole mobility, a more balanced charge-carrier transport, and increased photostability. Furthermore, flexible inverted OSC modules were fabricated by slot-die coating achieving a PCE of 5.25% and a power output of 419.6 mW. A layer of hydrogen molybdenum bronze (H_x_MoO_3_) with PEDOT:PSS layer was also used in all solution-processed non-fullerene OSCs based on PM6:IDIC:Y6 [315]. Phosphomolybdic acid (PMA) in PEDOT:PSS layers were tested in different organic fullerene-based OSCs, showing good performances [316]. GO, a two-dimensional carbon material, has also been investigated to modified PEDOT:PSS as hole-transport materials in different OSCs. The Oleyamine-functionalized GO/PEDOT:PSS layer on PBDB-T:ITIC [317], PEDOT:PSS treated with GO layers on PTB7:PC_71_BM devices [318], on P3HT:PC_60_BM devices [319], on P3HT:PCBM devices [320], on inverted P3HT:PCBM OSCs [321], on inverted P3HT:PC_71_BM OSCs [322], and on reduced GO-germanium QDs modified PEDOT:PSS on P3HT:PCBM [323]. Raj et al. reported the fabrication of PTB7:PC_70_BM-based OSCs with PEDOT:PSS:GO, resulting in enhanced PCE of 7.68% [324]. The modified HTL showed a fine fiber-like structure that improved the conductivity. GO showed to increase the device resistance degradation. GO is generally prepared by variations of the Hummers method using graphite powder as the starting material [325,326]. PEDOT:PSS:GO was also tested on P3HT:PC_61_BM-based OSCs, showing an increased J_sc_, FF, and a 14% higher PCE than a reference device [327]. GO in the HTL reduced the HOMO-LUMO gap and the R_s_, improving the hole mobility and the energy level matching. A double-decked GO/PEDOT:PSS HTL in PCDTBT:PC_71_BM-based OSCs was reported by Rafique et al. [328]. The modified HTL provided a better hole extraction and transportation by a suitable WF of GO (4.9 eV) and PEDOT:PSS (5.1 eV) that well-matched energy levels. This device showed an improved PCE of 4.28% ascribed to an increased charge-carrier mobility, J_sc_, V_oc_, and FF, and a reduced R_s_. Besides, better stability than PEDOT:PSS was reached, since GO served as a barrier that protected ITO corrosion due to the acidic nature of PEDOT:PSS (see Figure 17). Similarly, improved photovoltaic stability was achieved with GO/PEDOT:PSS HTLs in P3HT:PC_60_BM devices [329]. This device showed an increased R_sh_ and a decreased R_s_, which facilitate the hole transportation. The composite HTL was smooth and uniform, contributing to an improved device performance with a PCE of 4.82%. Nitrogen-doped graphene quantum dots (nGQDs) were blended with PEDOT:PSS HTLs in PTB7:PC_71_BM-based OSCs, resulting in an enhanced PCE of 8.5% [330]. The modified HTL improved the charge-carrier transport, increased the hole mobility, and suppressed charge recombination. The nitrogen doping led to a high content of quaternary nitrogen, enhancing the electrical conductivity of GQDs. UV-ozone (UVO)-treated GO/PEDOT:PSS bilayer in OSCs based on PCDTBT:PC_71_BM presented an improved PCE of 5.24% [331]. An increased J_sc_, V_oc_, and FF were obtained in the cells using the modified HTL and improved ambient stability, retaining above 90% of initial PCE after 240 h. The enhanced conductivity was ascribed to the reduction of oxygen content in GO after UVO treatment.

**Figure 18:**
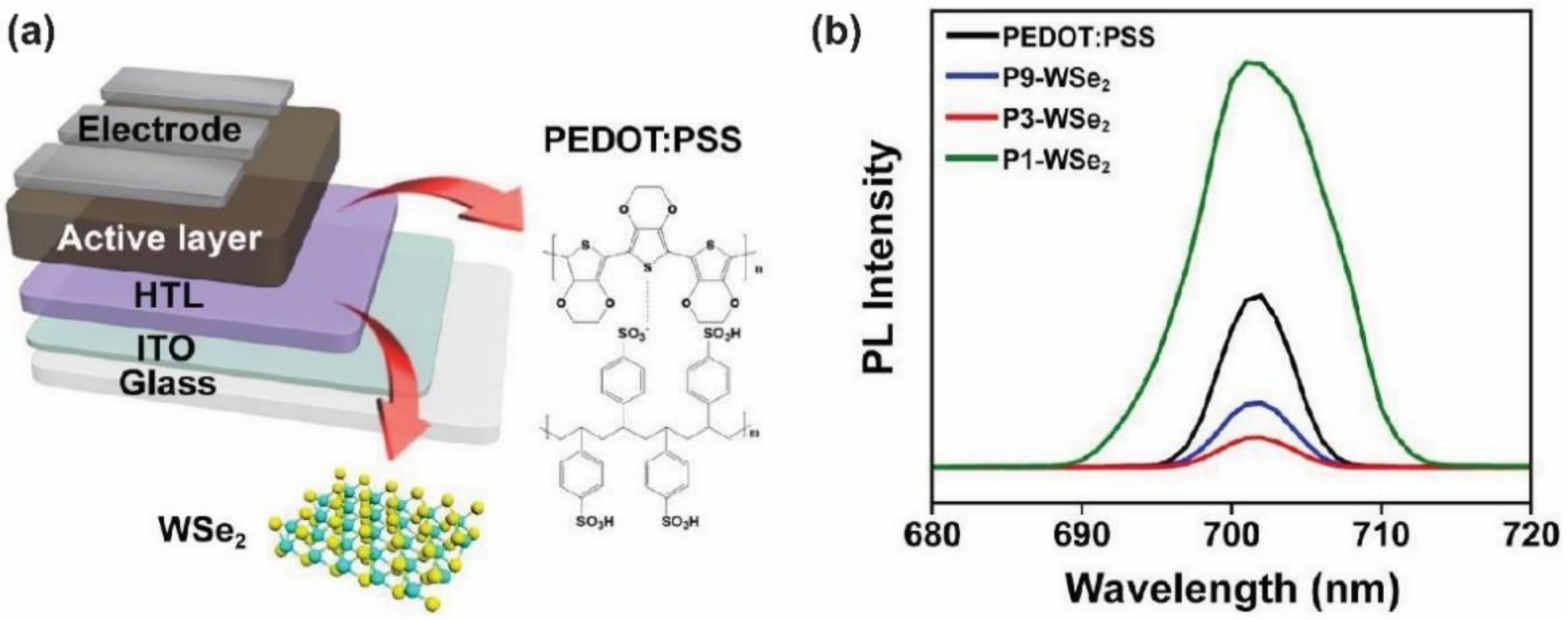
(**a**) Schematic representation of a WSe_2_-PEDOT:PSS HTL-based OSC and (**b**) PL spectra measured from structure of glass/PEDOT:PSS and P-WSe_2_-based devices. Adapted with permission from [333]. Copyright 2018, IOP Publishing Ltd.

Other modifications on PEDOT:PSS have also been reported with a graphene analog, the two-dimensional transition metal dichalcogenides. For instance, hybrid PEDOT:PSS/WS_2_ was incorporated as HTL in OSCs [332]. PEDOT:PSS worked as an effective exfoliating agent to the WS_2_ 2D structure. The photovoltaic device based on P3HT:PC_61_BM and PTB7-Th:PC_71_BM exhibited enhanced PCE of 3.07% and 7.24%, respectively, attributed to increased J_sc_ and FF as well as to enhanced hole mobility and enhanced conductivity of the PEDOT:PSS/WS_2_ layer. Besides, PEDOT:PSS/WS_2_-based OSCs had improved stability, retaining 77.3% of initial PCE after 36 days. Koo et al. fabricated PTB7:PC_71_BM-based OSCs with tungsten diselenide (WSe_2_)/PEDOT:PSS HTLs (see Figure 18a), showing an enhanced PCE of 8.5% [333]. The composite HTL exhibited a homogeneous film formation. WSe_2_ negative surface induced the segregation of PEDOT and PSS, which enhanced the layer conductivity. Furthermore, photoluminescence peak intensity decreased, indicating diminished recombination (see Figure 18b). Thus, PEDOT:PSS-WSe_2_ showed improved hole-transport ability and a better charge extraction than the reference device. Oleyamine-functionalized molybdenum disulfide MoS_2_ has also been reported in the modification of PEDOT:PSS HTLs [334].

Boronic acid functionalized multi-walled CNs (bf-MWCNTs)-doped PEDOT:PSS HTLs showed excellent hole mobility and electrical conductivity [335]. The OSC with PEDOT:PSS:bf-MWCNTs showed reduced R_s_ and increased R_sh_, exhibiting excellent hole collectivity. A 28% increased PCE for PCDTBT:PC_71_BM based OSC was attributed to an enhanced J_sc_ and FF. PL intensity of HTL doped with bf-MWCNTs was reduced, indicating an enhancement in charge transport from the active layer. The WF increased to 5.39 eV that well-matched with the HOMO energy level of PCDTBT. Graphitic carbon nitrile (g-C_3_N_4_) was used as a secondary dopant for PEDOT:PSS in OSCs based on PM6:Y6, leading to an improved PCE of 16.38% [336]. The g-C_3_N_4_:PEDOT:PSS HTL showed a higher conductivity, an improved charge transport, and a suppressed charge recombination. This modified HTL had increased hole mobility, leading to more balanced charge transport. The g-C_3_N_4_ insulated the PSS moiety, so the conducting PEDOT chain was exposed. A two-dimensional titanium carbide (Ti_3_C_2_T_x_) bilayer was incorporated into PEDOT:PSS HTLs in non-fullerene PBDB-T:ITIC and PM6:Y6 OSCs [337]. This bilayer enhanced the conductivity of PEDOT:PSS by a reduced coulombic attraction between PEDOT and PSS, causing the conformational transition of PEDOT from coil to linear structures. The HTL roughness increased upon Ti_3_C_2_T_x_ incorporation, which enlarged the contact area between HTL and the photoactive layer. The hole mobility increased because of the interconnected conducting network between PEDOT and Ti_3_C_2_T_x_. The PL peak was reduced, indicating improved hole transmission. As a consequence, the PCE of devices improved to 11.02% and 14.55% for PBDB-T:ITIC- and PM6:Y6-based OSCs, respectively. Moreover, PEDOT:PSS/Ti_3_C_2_T_x_ HTLs enhanced the nitrogen atmosphere’s long-term stability, retaining 79.67% of the initial PCE after 300 h.

#### 4.4.2. Other Conjugated Polymers

**Scheme 1:**
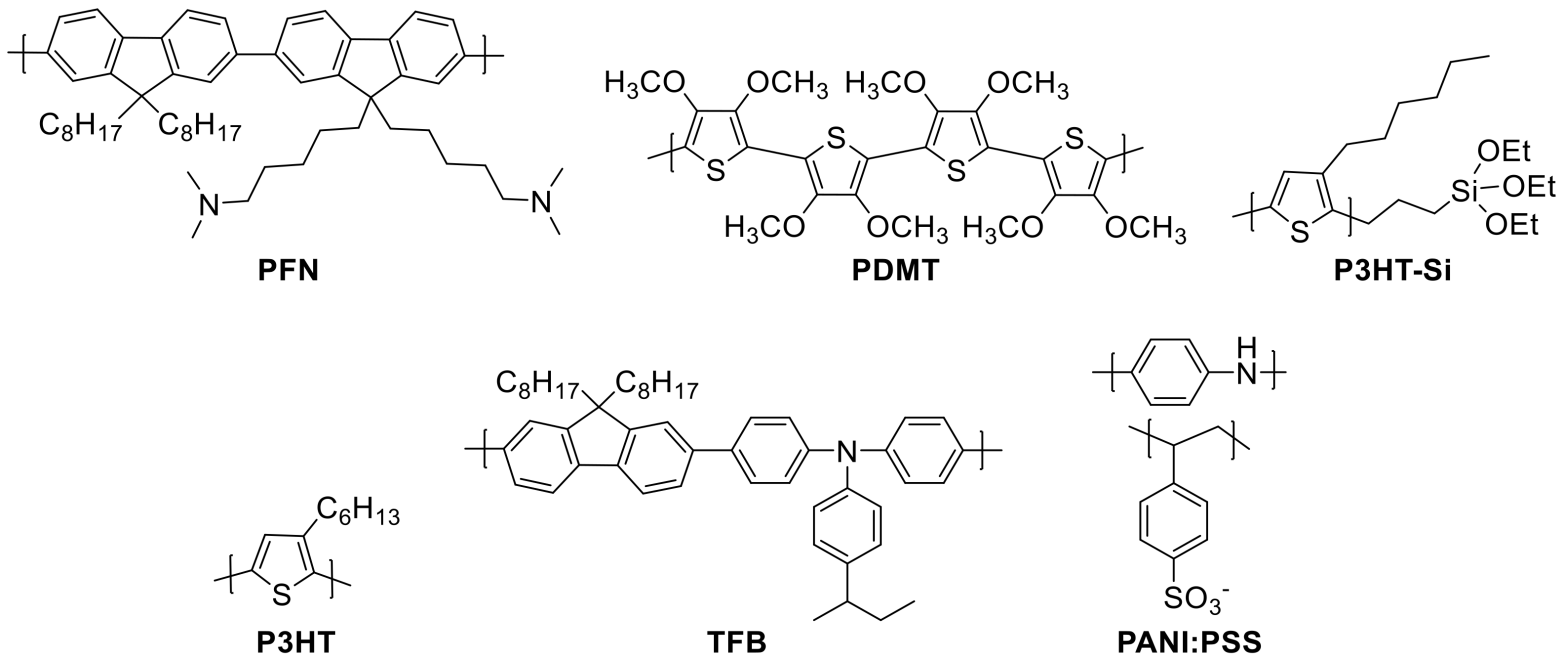
Chemical structure of some conjugated polymers used as HTLs.

Another approach aims to replace the use of PEDOT:PSS with different conjugated polymers. The chemical structure of a series of conjugated polymers used as HTL for OSCs are shown in Scheme 1.

A HTL nanocomposite based on fluorene derivatives, poly[(9,9-bis(3′-(*N*,*N*-dimethylamino)propyl)-2,7-fluorene)-alt-2,7-(9,9-dioctyl)fluorene] and nickel oxide (PFN/NiO_x_), showed a PCE of 6.2% in PBDTTBO-C_8_:PC_71_BM-based OSCs [338]. The device performance improvement was related to the interaction between PFN and NiO_x_, p-doping effect in NiO_x_, and good energy alignment. A blend of 5,6-difluorobenzothiadiazole conjugated polymer and metal oxide (Cu_2_O/FBT-TH4) produced a PCE of 9.56% for OSCs based on PffBT4T-2OD:PC_71_BM [339]. Better charge transfer properties and stability were determined, maintaining 75% of the original PCE for up to 30 days. This result was attributed to the hydrophobic character of the HTL. Poly(3,4-dimethoxythiophene) (PDMT) deposited via oxidative chemical vapor deposition were also used as hole-transport materials in OSCs [340]. Awada et al. fabricated OSCs based on hydrophobic triethoxysilane-terminated poly(3-hexylthiophene) (P3HT-Si) HTLs exhibiting a slightly enhanced device stability [341]. Some other polymers and composites used as hole-transport layers include P3HT:SWCNTs [342] and polyaniline/gold and silver NPs composites (Au_10_Ag_10_PANI) [343]. The interconnected network of grafted CNTs, polythiophenic agents, and conjugated PANI bottlebrushes (CNT-g-PDDT:P3ThEt-g-PANI) were used as HTL in OSCs based on PBDT-DTNT:PC_61_BM, showing smooth morphology, low sheet resistance, and a PCE of 5.65% [344]. A network of CNTs and polythiophene/polyaniline bottlebrushes (CNT:P3ThEt-g-PANI) was tested as HTL in OSCs based on P3HT:PC_71_BM, reaching an improved PCE of 5.30% [345]. Another approach involves the use of low acidic water-stable PSS-doped PANI as HTL based on P3HT:ICBA OSCs [346]. The PANI:PSS layer presented a well-matched WF, high conductivity, and transmittance around 90% that resulted in OSCs with PCE of 4.5%. Additionally, PANI:PSS HTL has also been tested for indoor photovoltaics [347,348]. The OSCs based on P3HT:ICBA showed a lower PCE than a device using PEDOT:PSS, but possessed better stability over 1176 h, retaining 39% of its initial PCE. PANI was also tested with GO as an acid-free composite HTL in OSCs based on P3HT:PCBM and PCDTBT:PC_71_BM, resulting in optimized performance for the nanocomposite with a GO loading of 7.3 wt% [349]. A hole-transporting bilayer of copper(I) thiocyanate and poly[(9,9-dioctylfluorenyl-2,7-diyl)-alt-(4,4′-(*N*-(4-butylphenyl)))] (CuSCN/TFB) was tested in OSCs based on non-fullerene PM6:Y6 and fullerene PTB7-Th:PC_71_BM by Dong et al. [350]. Better photovoltaic performance with the CuSCN/TFB bilayer than with pristine CuSCN HTL was related to enhanced J_sc_ and FF. The decreased roughness and increased contact angle of the bilayer favored the interfacial contact of the HTL and the active layer, leading to better energy matching and device performance (up to 15.10%). Furthermore, the CuSCN/TFB-based device presented improved hole mobility, higher exciton dissociation efficiency, and lower recombination loss, which contributed to its enhanced exciton dissociation and charge transportation and extraction.

**Scheme 2:**
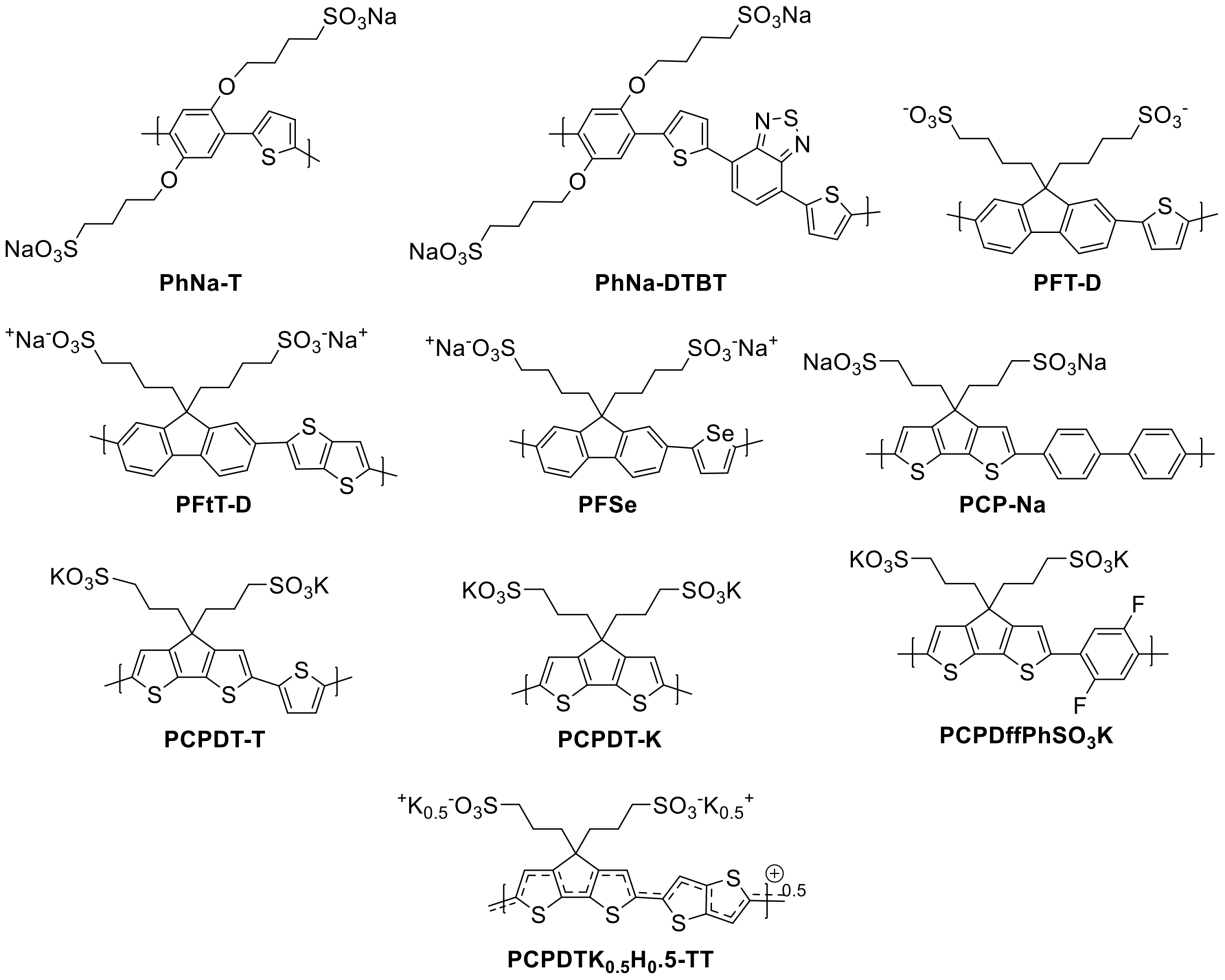
Chemical structure of some CPEs used as HTLs.

CPEs composed of conjugated backbone and side chains containing ionic groups are attractive materials due to their intrinsic dual electronic and ionic conductivity, and good solubility in polar solvents [351,352,353,354,355,356]. Some examples of the chemical structure of CPEs used as HTLs are shown in Scheme 2.

**Figure 19:**
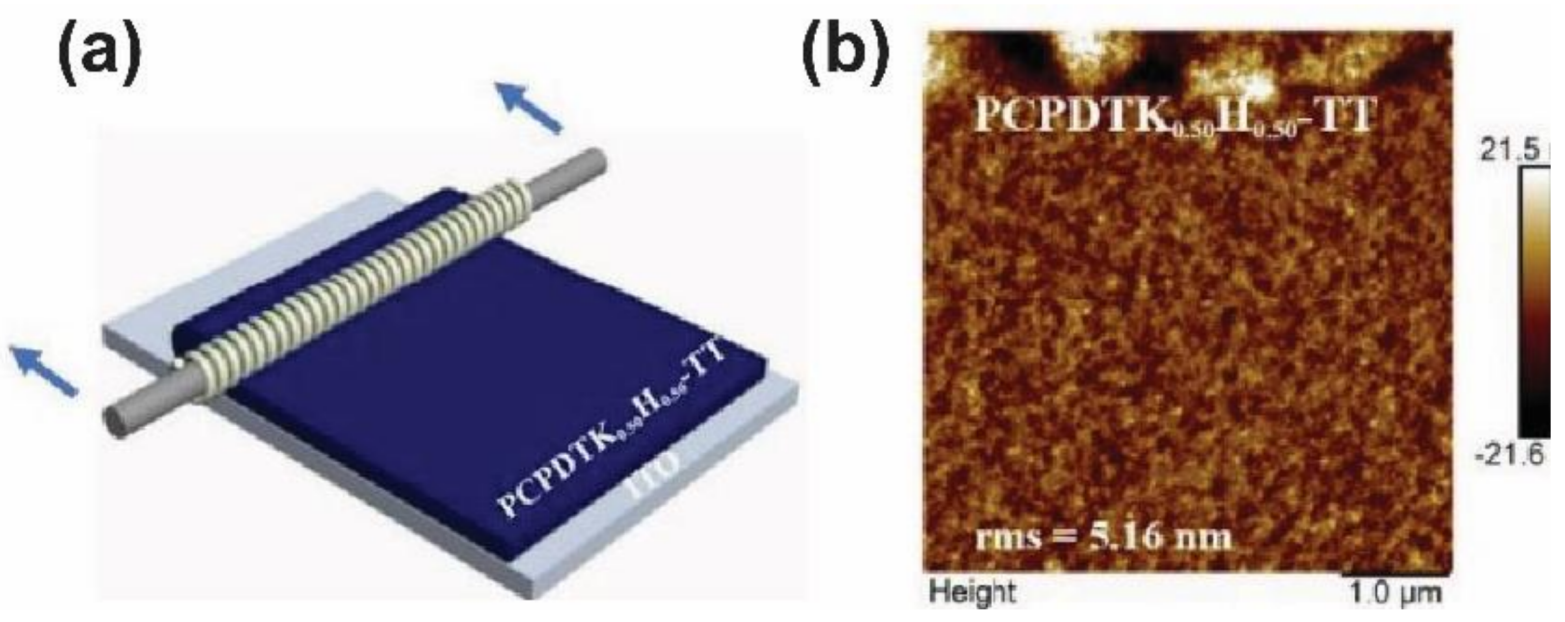
(**a**) Wire-bar coating process and (**b**) AFM image of the PCPDTK0.50H0.50-TT film prepared by the wire-bar coating process. Adapted with permission from [365]. Copyright 2021, American Chemical Society.

Poly[1,4-bis(4-sulfonatobutoxy)benzene-thiophene] (PhNa-1T) self-doped in a neutral state achieved an increased WF of 5.21 eV, resulting in PCEs of 9.89% and 8.38% for ITO/PhNa-1T/PTB7-Th:PC_71_BM/fullerene derivative (bis-C_60_)/Ag and ITO/PhNa-1T/PTB7:PC_71_BM/TiO_2_/Al cells, respectively [357]. The enhanced device performance was ascribed to improved interfacial properties, a high WF, and a smoother surface resulting in a favorable contact, improved charge extraction, and an efficient hole collection. PhNa-DTBT CPE, which is based on a weakly electron-donating 2-phenyl thiophene, an electron-acceptor, 2,1,3-benzothiadiazole, and sulfonate sodium salt as an ionic functional group were used in PTB7-Th:PC71BM OSCs, reaching a PCE of 9.29% [358]. PhNa-DTBT showed a high electrical conductivity, improved J_sc_ and FF, and high WF (5.39 eV). The device also showed improved stability with a retained PCE of ca. 40% after 96 h. A pH-neutral self-doped polymer based on phenyl and thienyl units, poly[2,6-(4,4-bis-(propane-1-sulfonate sodium)-4*H*-cyclopenta[2,1-b;3,4-b′]dithiophene)-alt-(4,4′-biphenyl)] (PCP-Na), was used as HTL for PBDT-TS1:PC_71_BM-based OSCs [359]. PCP-Na had a suitable HOMO level, smooth surface, and a high electrical conductivity due to the presence of polaronic states (radical cations). PCP-Na exhibited appreciable hole collection and charge-transport properties. The photovoltaic device using PCP-Na showed a PCE of 9.89% that resulted mainly from an enhanced FF. Following the same line, pH neutral poly[9,9-bis(4′-sulfonatobutyl)fluorene-alt-selenophene] (PFSe) was used as HTL in OSCs with architecture ITO/PFSe/PTB7:PC_71_BM/PFN/Al, exhibiting a PCE of 7.2% [360]. The increased J_sc_ and FF were ascribed to a strong dipole moment at the interface. A WF of 5.15 eV of PFSe assured a good ohmic contact and a better matching energy level. Moreover, the air stability of the cell was improved by the neutral nature of the HTL polymer. Xu et al. reported a pH-neutral CPE, 3,4-dithia-7*H*-cyclopenta[a]pentalene and thienyl units (PCPDT) used in OSCs based on PTB7-Th:PC_71_BM with a PCE of 9.3% [361]. Improved device performance by using PCPDT HTL was attributed mainly to a reduced leakage current and R_s_. A tuned WF of –4.87 eV, enhanced transmittance, and improved and homogeneous mobility of HTL were related to the strong p-type self-doped nature of this HTL. Moreover, the hole layer showed improved interface compatibility, evidenced by the reduced surface energy (30.7 mN m^−1^). The use of PCPDT-K HTL in OSC based on P3HT:PCBM showed improved device performance, increased J_sc_, reduced R_s_, a smooth surface, and better stability than a PEDOT:PSS reference device [362]. PCPDffPhSO_3_K, a neutral CPE based on 3,4-dithia-7*H*-cyclopenta[a]pentalene and 1,4-difluorobenzene units, was used as HTL for ITO/HTL/PTB7-Th:PC_71_BM/PFN/Al OSCs, resulting in PCE of 9.5% [363]. The self-doping effect in PCPDffPhSO_3_K improved its conductivity. A WF around −5.18 eV ensured a better energy level alignment, achieving a higher V_oc_, J_sc_, and hole mobility. Lee et al. utilized poly[9,9-bis(4′-sulfonatobutyl)fluorene-alt-thieno[3,2-b]thiophene] (PFtT-D) HTLs showing a PCE of 8.3% for OSCs based on PTB7-Th:PC_71_BM [364]. The WF of the modified electrode with PFtT-D was 5.19 eV, which resulted in superior ohmic contact due to well-matched energy levels facilitating the hole transportation. The modification of WF was attributed to the molecular dipole orientations. The device showed an improved lifetime because of the neutral nature of CPE; the PCE slowly decreased with a half-life of 153 h. PCPDTK_0.50_H_0.50_-TT, a neutral self-doped CPE, was used as HTL for the OSCs based on PM6:Y6:PC_71_BM [365]. Potassium ions were exchanged to protons through ion-exchange chromatography using acid-sulfonated polystyrene resin. PCPDTK_0.50_H_0.50_-TT HTL had a higher WF and increased mobility, and it also exhibited a higher hole-extraction efficiency. The device performance with this HTL was improved with a PCE of 16.3%. The V_oc_ increased due to the improved hole mobility, and the J_sc_ and FF were also improved, ascribed to reduced carrier recombination and reduced bulk resistance. The device showed improved stability, and the PCE was retained by a longer time than PEDOT:PSS-based reference cells. OSCs with an area of 1.0 cm^2^ prepared by wire-bar coating achieved a PCE greater than 10%, showing potential for large-area printing techniques (see Figure 19).

### 4.5. Small Organic Molecules

**Scheme 3:**
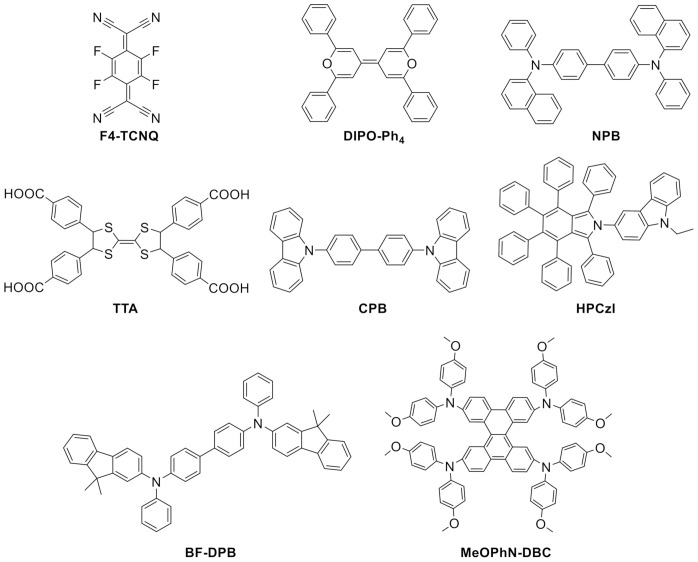
Chemical structure of some small organic molecules used as HTLs.

As an alternative to conjugated polymers, small organic molecules can be used as HTLs for photovoltaic applications [36]. Polymeric materials shown in the previous section have several drawbacks e.g., complicated synthesis, costly purification processes, and precise control of their molecular weight. In general, polymeric HTL materials used in OSCs usually have molar mass over 10,000 g mol^−1^, which makes them expensive [366]. Moreover, the hole mobilities of polymers such as PTAA are sensitive to molecular weights, polydispersity indices, and purities [367]. HTLs based on small organic molecules, compared with inorganic and polymeric materials, present a variety of benefits such as simple synthesis, structural versatility, high purity, and tunable energy levels [368]. Some examples of the molecular structure of small molecules used as HTL are shown in Scheme 3.

**Figure 20:**
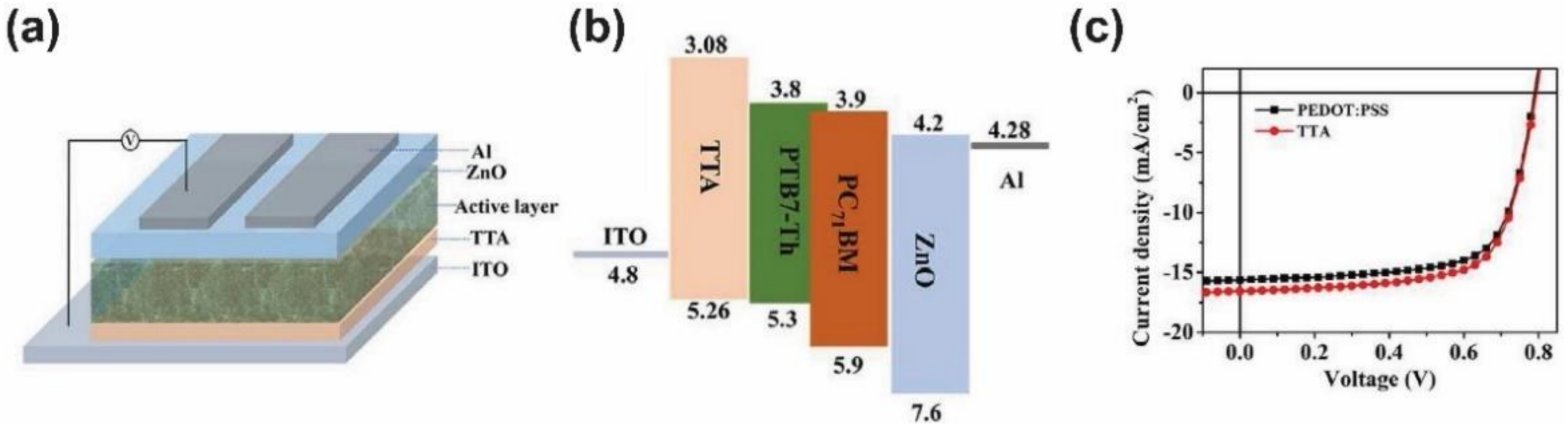
(**a**) Illustration of OSCs structure based on TTA, (**b**) energy level diagram of different layers, and (**c**) J-V curves of devices with TTA and PEDOT:PSS as HTLs. Adapted with permission from [377]. Copyright 2020, Elsevier.

NDP9 doped *N*,*N*′-((diphenyl-*N*,*N*′-bis)9,9,-dimethyl-fluoren-2-yl)-benzidine (BF-DPB) was used as a hole-transport material in OSCs based on zinc phtalocyanine (ZnPC):fullerene C_60_ [369]. Spin-coated BF-DPB HTLs over AgNWs electrodes exhibited a PCE of 4.4%. BF-DPB smoothed the AgNWs topography. Cheng et al. fabricated NiOx/2,3,5,6-tetrafluoro-7,7,8,8-tetracyanoquinodimethane (F4-TCNQ) composite HTLs for the fabrication of OSCs without pre-treatment of ITO nor post-treatment on the HTL [370]. The device performance of one-step ethanol-processed NiOx:F4-TCNQ in P3HT:PC_61_BM-based OSCs was 15.8% better than one-step PEDOT:PSS-based OSCs. NiOx:F4-TCNQ HTL was also used on PTB7-Th:PC_71_BM-based OSCs, resulting in an enhanced PCE of 8.59%. A planar quinoid molecule, 2,2′,6,6′-tetraphenyl-dipyranylidene (DIPO-Ph_4_), was tested as an anodic interfacial layer with PEDOT:PSS in P3HT:PCBM OSCs [371]. Vacuum-deposited DIPO-Ph_4_ (10 nm thickness) on spin-coated PEDOT:PSS (5 nm thickness) increased OSCs’ current and enhanced efficiency to 4.6%. DIPO-Ph_4_’s needle-like morphology increased the contact area between the active layer and the anode with high hole conductivity. 1,3,4,5,6,7-Hexaphenyl-2-{3′-(9-ethylcarbazolyl)}-isoindole (HPCzI) HTLs exhibited improved performance in comparison with MoO_3_-based OSCs, reaching a PCE of 1.69% for CuPC:C60-based OSCs, due to larger FF and J_sc_ [372]. *N*,*N*′-bis(1-naphthalenyl)*N*,*N*′-bis-phenyl-(1,1′-biphenyl)-4,4′-diamine (NPB) was incorporated as HTL on inverted P3HT:PC_71_BM OSCs, resulting in a PCE of 2.63%, a J_sc_ of 9.49 mA cm^−2^, and low R_s_ [373]. These results suggested the formation of an ohmic contact between the photoactive layer and anode, which contributed to the hole extraction efficiency. Alternatively, the NPB layer was inserted between the MoO_3_ layer and the photoactive layer in inverted OSCs based on P3HT:PC_61_BM [374]. The PCE was enhanced from 3.20% to 3.94%, owing to the increased J_sc_ and reduced R_s_ by an improved charge transportation and reduced recombination at the interface. MoO_3_ p-doped 4,4′-*N*,*N*′-dicarbazole-biphenyl (CBP:MoO_3_) was also utilized as HTLs in inverted P3HT:PC_61_BM-based OSCs [375]. A 3,6,11,14-Tetramethoxyphenylamine-dibenzo[g,p]chrysene (MeOPhN-DBC) layer was incorporated between MoO_3_ and active-layer P3HT:PC_61_BM-inverted OSCs, showing an enhanced PCE of 3.68%, attributed to improved J_sc_ and FF, and reduced leakage current [376]. Liu et al. reported a tetrathiafulvalene derivative with four carboxyl groups (TTA) as an HTL in OSCs (see Figure 20a) [377]. This HTL displayed a well-matched energy level (see Figure 20b) and an enhanced PCE of 9.09% in comparison with a PEDOT:PSS-based OSC (see Figure 20c). The improved FF and J_SC_ were related to the smooth surface of the TTA layer, improved charge transfer and hole mobility, and reduced charge recombination.

Large-scale printing processes, like roll-to roll, require that the prepared small molecules present good adhesion and compatibility between the anode and the active layer, high carrier transport, good stability, and high solubility in non-pollutant solvents. Flexible devices will additionally require having mechanical flexibility of the layer. Convenient modification of certain functional groups allows the tuning of the electronic properties of the material to match the work function and conductivity. The research activity in these fields is very active, representing the main challenges to pave the road towards the wide commercialization of the OSCs [78].

Finally, Table 2 enlists a series of HTLs based on organic conjugated polymers and small molecules. The anode configuration with its work function, deposition technique for the HTL, active layer composition, and the OSCs performance parameters are provided as well as the reference where the information was taken from.

## 5. Conclusions

In summary, HTLs are fundamental to assure the high performance and stability of OSCs. Inorganic and nanocarbon materials including MoO_3_, WO_3_, V_2_O_5_, NiO_x_, CuO_x_, CoO_x_, CuCrO_x_, CuSCN, MoS_2_, WS_2_, NiS, CuS and GO, QCDs, and CNTs have shown great potential as HTLs in conventional and inverted OSCs. These hole-extracting materials can form an ohmic contact between the active and electrodes depending on their optical and electrical properties. Their high transparency enables them to absorb high light into the active layer to afford the hole-electron pairs generation, and the tuning of the Fermi levels with the donor allows the hole collection. Usually, the hole transport takes place in the HTL valence band, but in *n*-type metals such as MoO_3_, it has been found that the conduction band facilitates the hole transport. Thus, the type of hole-transport path will vary with the WF and energy levels of the inorganic and nanocarbon materials as HTLs. Modification in the particles’ size or addition of metal NPs results in the LSPR effect, which increases the light absorption. Inorganic materials such as Mo and Ni were doped with V and Cu to tune the WF and increase conductivity and transparency, resulting in a high V_oc_, FF, and J_sc_. Hybrid layers, such as MoS_2_:MoO_3_, improved the electron-blocking properties and increased conductivity of the layer. Nanocarbon materials such as GO were doped with F_4_TCNQ to induce a change in the WF by shifting the Fermi levels, resulting in an enhanced hole transport. CNTs were functionalized with amino groups to increase the charge-carrier properties and reduce R_s_, improving FF and J_sc_. HTLs can also be subjected to ultraviolet ozone (UVO), annealing, and microwave-annealing post-treatments to increase V_oc_, FF, and J_sc_ due to the reduction of oxygen defects in the surface morphology. The most-used conjugated polymer is the PEDOT:PSS and its composites such as PEDOT:PSS with NPs, MOs, and GO either in bilayer or composite monolayer. PANI was the second choice of conducting polymers as HTLs. The different modification to PEDOT:PSS in many cases increases the J_SC_, improves the conductivity, and decreases the recombination loss of the device. In general, the addition of metallic NPs to PEDOT:PSS enhances the absorption ability of the photoactive layer, increases the conductivity, and improves the charge carrier collection by increasing the device performance. The incorporation of metal oxides mainly helps to (i) increase the stability of the device (e.g., MoO_3_) by mitigating the degradation, (ii) serve as an electron blocking layer (e.g., V_2_O_5_), and (iii) suppress the charge recombination. On the other hand, GO addition to PEDOT:PSS improves the conductivity and increases the device resistance to degradation. Furthermore, CPEs were also used as HTL for OSCs; in general, these materials were pH-neutral layers that improved the stability of the devices and showed high electrical conductivity and good interface compatibility. In the last decade, the standard HTL small molecule has been spiro-OMeTAD; nevertheless, this review pointed out that research to optimize these materials is growing in activity and importance. Important points like shorter and more efficient chemical synthesis, access from cheaper starting materials, analysis of the active layer (perovskite or organic) interactions with the HTL small molecules, as well as better understanding of the charge transport process (carrier diffusion, recombination process) makes this field very important for optimization of the solar cells.

Additionally, large-area deposition techniques are mandatory to facilitate the commercialization of organic photovoltaics. Compared with the conventional spin-coating technique, laser-assisted and electrospray techniques allow the control of the surface morphology and thickness at low temperatures and short-time processing. The roll-to-roll technique is also attractive for large industrial-scale manufacturing of metal oxides, such as the inkjet printing of NiO_x_. Overall, inorganic and nanocarbon HTLs are very favorable for OSCs, mainly because of their high stability, improved electrical properties, and transparency in the visible range. Solution processing is a great advantage of using small organic molecules as HTL. The continuous investigation of a vast number of new inorganic and organic HTLs, which can assure high efficiency, high stability, low costs, facile preparation, and improved film-forming properties over large areas, is essential for the future commercialization of OSCs. Therefore, not only the chemical or electrochemical properties of the prepared HTL materials are important to study, but also it is required to develop materials that fulfill the technological requirements to apply them at large scales. The future in this direction looks very promising.

## Figures and Tables

**Table 1:** Performance parameters of OSCs with inorganic films as HTLs.

Anode Configuration and WF (eV)	Deposition Technique	Active Layer	V_OC_ (V)	J_SC _ (mA cm^−2^)	FF (%)	PCE (%)	Ref.
*Metallic oxides*							
ITO/s-MoO_3_ (4.92)	spin coating	PBDB-T-2F:Y6	0.84	27.53	73.10	17.00	[107]
ITO/PCM4 (5.4)	blade coating	PBDB-T-2F:Y6	0.83	16.06	68.28	14.30	[119]
HVO/Ag (6.7)	spin coating	PBDTT-FTTE:PC71BM	0.82	22.51	60.19	11.14	[159]
ITO/MoO_3_ (5.3)	spin coating	PBDB-T:PC71BM	0.88	17.48	71.00	10.86	[102]
MoO_3_:NiO_x_ (5.1)	spin coating	PBDB-T:IT-M	0.94	17.26	66.63	10.81	[113]
e-MoO_x_/Ag (5.4)	spin coating	PTB7-Th:PC71BM	0.79	18.70	69.20	10.42	[99]
VO_x_:PEDOT:PSS/Ag (5.28)	spin coating	TPD-3F:IT-4F	0.87	16.80	69.10	10.10	[164]
MoO_3_/AgAl/MoO_3_/AgAl	thermal evaporation	PTB7-Th:PC71BM	0.78	19.60	61.90	9.79	[100]
CuBr-MoO_3_/Ag (5.03)	thermal evaporation	PTB7:PC71BM	0.75	19.65	65.20	9.56	[117]
MoO_x_ NPs/Ag (5.40)	spin coating	PTB7- Th:PC 71 BM	0.79	18.05	65.20	9.50	[99]
ITO/MoO_3_ (5.29)	spin coating	PBDB-T:ITIC	0.91	15.19	66.59	9.17	[106]
ITO/NiO_x_ NPs(5.25)	spin coating	PTB7-Th:PC71BM	0.79	18.32	63.10	9.16	[176]
ITO/s-MoO_3_ (4.92)	spin coating	PTB7-Th:PC71BM	0.79	16.69	67.10	8.90	[107]
ITO/CuO_x_ (5.06)	spin coating	PTB7:PC71BM	0.74	16.44	71.00	8.68	[187]
MoO_3_/Ag (5.52)	thermal evaporation	PTB7-Th:PC70BM	0.81	15.90	67.80	8.67	[98]
ITO/p-MoO_3_ (5.26)	spin coating	PTB7:PC71BM	0.73	17.02	68.10	8.46	[104]
ITO/CTAB-MoO_3_ (5.18)	spin coating	PTB7:PC71BM	0.72	16.88	68.10	8.34	[118]
ITO/VO_x_·nH_2_O (5.0)	spin coating	PTB7-th:PC71BM	0.78	15.76	64.62	8.11	[156]
ITO/V_2_O_5_	spin coating	PTB7:PC70BM	0.71	17.35	65.00	8.05	[154]
ITO/NiO_x_ NPs(5.25)	spin coating	PTB7:PC71BM	0.74	16.10	66.42	7.96	[176]
ITO/NP-V_2_O_5_ (4.7)	spin coating	PTB7:PC70BM	0.72	15.81	69.01	7.89	[165]
WO_x_ nanosheets/Ag	spin coating	PTB7: PC71BM	0.81	16.42	58.19	7.76	[150]
ITO/s-VO_x_ (5.3)	spin coating	PTB7:PC71BM	0.73	15.79	66.82	7.70	[160]
ITO/MoO_3_/AuNPs/MoO_3_ (5.6)	spin coating	PTB7:PC70BM	0.73	14.40	73.00	7.68	[122]
ITO/ESD-VO_x_ (5.6)	spray casting	PTB7:PC71BM	0.74	15.30	67.00	7.61	[168]
ITO/V_2_O_x_ (5.42)	spin coating	PBDTDTTT-S-T:PC71BM	0.68	16.29	67.21	7.44	[114]
ITO/MoO_x_ (5.30)	spin coating	PBDTDTTT-S-T:PC71BM	0.69	16.14	66.02	7.35	[114]
ITO/sMoO_x_	spin coating	PV10:PC70BM	0.73	13.57	72.55	7.19	[101]
FTO/Cu:NiO_x_	spin coating	PCDTBT:PC71BM	0.89	12.40	63.85	7.05	[181]
FTO/c-NiO (5.0)	spin coating	PTB7:PC71BM	0.72	14.28	66.98	6.91	[178]
ITO/MoO_x_	thermal evaporation	PTB7:PC71BM	0.67	14.00	67.00	6.57	[103]
ITO/H_y_MoO_3-x_ (5.6)	spin coating	PTB7:PC70BM	0.77	13.90	61.20	6.55	[129]
ITO/NiO_x_ NPs(5.25)	spin coating	PCDTBT:PC71BM	0.90	11.36	62.35	6.42	[176]
ITO/NiO_x_ (5.6)	spin coating	TQ1:PC70BM	0.87	10.30	71.30	6.39	[175]
ITO/s-V_2_O_x_ (5.25)	spin coating	PFDT2BT-8:PC70BM	0.87	10.20	67.10	6.30	[162]
V_2_O_x_/Ag (5.42)	spin coating	PBDTDTTT-S-T:PC71BM	0.63	15.81	61.02	6.08	[114]
MoO_x_/Ag (5.30)	spin coating	PBDTDTTT-S-T:PC71BM	0.61	15.68	62.78	6.00	[114]
ITO/V_2_O_5_·H_2_O (5.04)	spin coating	PBDSe-DT2PyT:PC71BM	0.72	13.96	59.00	5.87	[155]
ITO/CTAB-MoO_3_ (5.18)	spin coating	P3HT:ICBA	0.82	10.40	67.40	5.80	[118]
ITO/MoO_x_	thermal evaporation	PTB7-Th:PC71BM	0.74	14.50	44.80	5.52	[103]
FTO/s-MoO_3_ (5.3)	spin coating	P3HT:ICBA	0.82	11.50	58.00	5.40	[105]
FTO/MoO_x_ (5.6)	spin coating	P3HT:PC71BM	0.65	12.72	61.00	5.00	[110]
*Metallic sulfides*							
ITO/WS_2_ (5.3)	spin coating	PBDB-T-2F:Y6:SF(BR)_4_	0.89	29.31	80.00	20.87	[204]
ITO/WS_2_ (5.5)	spin coating	PBDB-T-2F: Y6: PC71BM	0.84	26.00	78.00	17.00	[203]
ITO/WS_2_ (5.1)	spin coating	PBDB-T-2F:Y6:PC71BM	0.83	26.00	72.00	15.60	[202]
ITO/CuSCN:TFB (1.0%) (5.50)	spin coating	PM6:Y6	0.85	24.35	73.84	15.28	[214]
ITO/MoS_2_ (5.04)	spin coating	PBDB-T-2F:Y6:PC71BM	0.81	25.30	71.00	14.90	[202]
ITO/CuSCN:AMQS	spin coating	PBDBT-2F:IT-4F	0.80	18.70	67.80	10.14	[209]
ITO/CuSCN:AMQS	spin coating	PTB7-Th:PC71BM	0.79	17.10	65.20	8.80	[209]
ITO/O-MoS_2_ QDs (5.2)	spin coating	PTB7-Th: PC71BM	0.79	16.90	65.00	8.66	[200]
ITO/MoS_x_ (5.10)	spin coating	PTB7-Th: PC71BM	0.77	18.16	53.56	7.50	[201]
ITO/CuSCN:AMQSs	spin coating	PTB7-Th:ITIC	0.82	15.07	59.06	7.15	[209]
ITO/CuSCN:C_60_F_48_ (5.40)	spin coating	PCDTBT:PC70BM	0.92	11.50	61.00	6.60	[213]
*Nanocarbon materials*							
ITO/uSWNTs/PEDOT:PSS	spin coating	PBDB-T-2F:IT-4F	0.85	23.39	73.17	14.60	[254]
ITO/FrGO (4.9)	spray casting	PM6:Y6 PSC.	0.77	24.64	69.60	13.26	[235]
L-GO:NiO/Ag	spin coating	PBDB-T:IT-M	0.91	17.81	71.00	12.13	[245]
G-MoS_2_/Ag (4.42)	spin coating	PTB7-Th:PC71BM	0.80	17.10	67.70	9.50	[236]
ITO/G-MoS_2_/PEDOT:PSS (5.0)	spin coating	PTB7-Th:PC71BM	0.77	17.20	72.00	9.40	[236]
ITO/FrGO (4.9)	spray casting	PTB7-Th:EH-IDTBR	1.00	14.86	61.80	9.22	[236]
ITO/FrGO (5.1)	spin coating	PTB7-Th:PC71BM	0.79	16.89	64.80	8.60	[233]
ITO/P-GO (4.70)	spin coating	PTB7:PC71BM	0.71	16.12	68.40	7.90	[232]
ITO/GO:CuCl_2_ (5.1)	spin coating	PTB7-Th:PC71BM	0.79	15.52	63.00	7.74	[240]
ITO/G-MoO_3_ (5.32)	spin coating	PCDTBT:PC71BM	0.86	12.83	63.67	7.07	[246]
af-MWNTs (5.22)	spin coating	PCDTBT:PC71BM	0.87	12.65	63.50	6.97	[252]
ITO/GO:NPs (4.9)	spin coating	PTB7:PC71BM	0.75	11.55	67.91	5.88	[242]
ITO/F_5_-rGO (5.1)	spin coating	PTB7:PC71BM	0.68	14.78	57.30	5.82	[234]
ITO/GBD (4.9)	spin coating	PBDTTT-C-T:PC70BM	0.71	13.38	52.54	5.01	[241]
ITO/GO:MoO_3_ (5.3)	spin coating	PCDTBT:PC71BM	0.66	16.16	47.11	5.10	[244]

**Table 2:** Performance parameters of some representative OSCs with different organic conjugated polymers and small molecules as HTLs.

Anode Configuration and WF (eV)	Deposition Technique	Active Layer	VOC (V)	JSC (mA cm−2)	FF (%)	PCE (%)	Ref.
*PEDOT:PSS*							
ITO/PEDOT:PSS-DA (5.14)	spin coating	PM6:Y6	0.84	25.52	77.1	16.55	[273]
ITO/g-C_3_N_4_:PEDOT:PSS (4.89)	spin coating	PM6:Y6	0.84	26.71	73.0	16.38	[336]
ITO/PEDOT:PSS:TEMPO^+^Br^-^ (4.95)	spin coating	PM6:Y6	0.82	27.18	72.6	16.10	[270]
ITO/PEDOT:PSS:a-In_2_Se_3_ (5.06)	spin coating	PM6:Y6	0.84	25.47	74.5	15.89	[272]
ITO/WO_x_:PEDOT:PSS (4.7)	spin coating	PM6:IT-4F	0.87	20.73	80.8	14.57	[313]
ITO/PEDOT:PSS/Ti_3_C_2_T_x_ (5.0)	selectively etching/spin coating	PM6:Y6	0.83	25.63	68.4	14.55	[337]
ITO/PEDOT:PSS-MoO_3_ (5.22)	spin coating	PBDB-T-2F:IT-4F	0.86	21.71	70.6	13.19	[311]
ITO/PEDOT:PSS/BPQD (4.92)	spin coating	PM6:IT-4F	0.85	21.14	71.3	12.81	[297]
AgNWs/PEDOT:PSS/H_x_MoO_3_ (5.44)	transfer printing	PM6:IDIC:Y6	0.83	21.00	68.0	11.90	[315]
ITO/PEDOT:PSS:a-In_2_Se_3_ (5.06)	spin coating	PBDB-T:ITIC	0.91	17.31	71.1	11.22	[272]
ITO/PEDOT:PSS/Ti_3_C_2_T_x_ (5.0)	selectively etching/spin coating	PBDB-T:ITIC	0.91	17.08	70.9	11.02	[337]
ITO/NiFD:PEDOT:PSS (5.01)	spin coating	PM6:PC_71_BM	0.98	13.82	79.4	10.76	[269]
WO_3_/PEDOT:PSS/Ag (5.27)	spin coating	SMD2:ITIC-Th	0.90	17.30	66.0	10.30	[314]
ITO/Ag ND/PEDOT:PSS	LIL/spin coating	PTB7:PC_70_BM	0.73	23.26	61.0	10.11	[291]
ITO/PEDOT:PSS-AuNRs	spin coating	PTB7-Th:PC_71_BM-Au NRs	0.80	17.90	68.8	9.89	[300]
ITO/V_2_O_5_: PEDOT:PSS	spin coating	PTB7-Th:PC_71_BM	0.80	16.83	70.1	9.44	[309]
ITO/PEDOT:PSS/TTF-py (5.29)	spin coating	PTB7-Th:PC_71_BM	0.79	17.19	70.6	9.37	[268]
ITO/PEDOT:PSS + Au NPs (5.4)	spin coating	PTB7:PC_71_BM	0.74	18.30	68.0	9.26	[290]
ITO/PEDOT:PSS/BPQD (4.92)	spin coating	PTB7-Th:PC_71_BM	0.80	16.40	69.4	9.11	[297]
ITO/PEDOT:PSS/p-TPCF (5.28)	electrochemical cyclic voltammetry	PTB7-Th:PC_71_BM	0.80	16.98	66.2	8.99	[285]
ITO/GOs/PEDOT:PSS (4.55)	spin coating	PBDB-T:ITIC	0.90	15.10	65.7	8.93	[317]
PMA:PEDOT:PSS/Al (5.02)	spin coating	PTB7-Th:PC_71_BM	0.79	17.10	68.0	8.88	[316]
PMA:PEDOT:PSS/Al (5.02)	spin coating	PffBT4T-2OD:PC_71_BM	0.77	18.44	64.0	8.75	[316]
ITO/PEDOT:PSS/PTPCz (5.23)	spin coating/electrodeposition	PTB7:PC_71_BM	0.74	16.23	71.1	8.54	[284]
ITO/PEDOT:PSS-WSe_2_	spin coating	PTB7:PC_71_BM	0.78	16.60	65.5	8.50	[333]
ITO/PEDOT:PSS:Cu-Au NPs	spin coating	PTB7-Th:PC_71_BM	0.79	17.78	60.1	8.48	[294]
ITO/PEDOT:GSL (5.05)	spin coating	PTB7-Th:PC_71_BM	0.77	15.82	68.7	8.47	[259]
ITO/GO/PEDOT:PSS (4.9)	chemical vapor deposition/drop casting	PTB7:PC_71_BM	0.75	16.10	69.5	8.40	[318]
ITO/PEDOT:PSS:FOS (4.90)	spin coating	PTB7:PC_70_BM	0.70	16.94	69.3	8.26	[263]
ITO/PEDOT:PSS+PFT-D (5.0)	spin coating	PTB7-Th:PC7_1_BM	0.77	14.90	71.3	8.20	[286]
FTO/PMMA/PEDOT:PSS	nanoimprinting/spin coating	PTB7:PC_70_BM	0.73	16.30	68.2	8.12	[274]
ITO/PSS:PEDOT:PSS (4.80)	spin coating	PDCBT:PC_71_BM	0.83	12.44	77.2	7.97	[269]
ITO/PEDOT:PSS:GO (5.1)	spin coating	PTB7:PCBM	0.75	14.90	67.5	7.68	[324]
PEDOT:PSS+MoO_3_ NPs/Ag (5.0)	spin coating/blade coated	PTB7-Th:PC_60_BM	0.78	14.99	63.0	7.39	[310]
ITO/PEDOT:PSS:Ag-Au-Au NRs	spin coating	PTB7:PC_71_BM	0.73	16.87	60.0	7.36	[293]
ITO/PEDOT:PSS/WS_2_	spin coating	PTB7-Th:PC_71_BM	0.79	15.67	58.6	7.24	[332]
ITO/PEDOT:PSS:Cu-Au NPs	spin coating	PTB7-Th:PC_61_BM	0.80	15.50	57.9	7.13	[294]
ITO/PEDOT:PSS:bf-MWCNTs (5.39)	spin coating	PCDTBT:PC_71_BM	0.88	12.51	63.1	6.95	[335]
ITO/PEDOT-S (5.2)	spin coating	P3TI:PC_71_BM	0.73	12.80	72.0	6.70	[265]
ITO/PEDOT:PSS-NiS	spin coating	P3HT:PC_61_BM	0.58	18.65	55.9	6.03	[288]
ITO/PEDOT:PSS + Au NPs (5.0)	spin coating	rrP3HT:PC_71_BM	0.58	16.10	61.0	5.65	[292]
ITO/PEDOT:PSS + Au NPs (5.0)	spin coating	rrP3HT:PC_71_BM	0.58	14.70	61.0	5.29	[292]
ITO/PEDOT:PSS:PSFP-DTBTP (5.14)	spin coating	PCDTBT:PC_71_BM	0.88	9.46	66.3	5.26	[275]
ITO/GO/PEDOT:PSS (4.9)	spin coating	PCDTBT:PC_71_BM	0.85	10.82	57.0	5.24	[331]
ITO/PEDOT:PSS:GO (5.52)	spin casting	PTB7:PC_71_BM	0.65	15.17	53.0	5.22	[327]
ITO/PEDOT:PSS-MoO_3_ (5.3)	spray deposition	PTB7:PC_71_BM	0.69	15.20	48.3	5.11	[307]
PMA:PEDOT:PSS/Ag NWs (5.02)	doctor-blade coating	PTB7-Th:PC_71_BM	0.78	11.28	57.0	5.01	[316]
*Other conjugated polymers*							
ITO/PCPDTKH-TT (5.24)	wire-bar coating	PM6:Y6:PC_71_BM	0.85	25.10	75.9	16.30	[365]
ITO/CuSCN/TFB (5.32)	spin coating	PM6:Y6	0.85	24.45	72.7	15.10	[350]
ITO/PhNa-1T (5.21)	spin coating	PTB7-Th:PC_71_BM	0.79	16.98	71.1	9.89	[357]
ITO/PCP-Na (5.22)	spin coating	PBDT-TS1:PC_71_BM	0.80	17.46	70.6	9.89	[359]
ITO/Cu_2_O/FBT-TH4 (5.08)	sputtered method	PffBT4T-2OD:PC_71_BM	0.77	17.50	70.7	9.56	[339]
ITO/PCPDffPhSO3K (5.18)	spin coating	PTB7-Th:PC_71_BM	0.79	18.08	67.0	9.50	[363]
ITO/PCPDT-T (4.87)	spin casting	PTB7-Th:PC_71_BM	0.77	18.92	63.5	9.30	[361]
ITO/PhNa-DTBT (5.3)	spin coating	PTB7-Th:PC_71_BM	0.79	16.92	69.5	9.29	[358]
ITO/CuSCN/TFB (5.32)	spin coating	PTB7-Th:PC_71_BM	0.79	16.42	66.3	8.56	[350]
ITO/PhNa-1T (5.21)	spin coating	PTB7:PC_71_BM	0.75	16.17	68.6	8.38	[357]
ITO/PFtT-D (5.19)	spin coated	PTB7-Th:PC_71_BM	0.76	16.00	68.4	8.30	[364]
ITO/PFSe (5.1)	spin casting	PTB7:PC_71_BM	0.68	14.40	69.0	7.20	[360]
ITO/NiO_x_:PFN (5.34)	spin casting	PBDTTBO-C8:PC_71_BM	0.71	13.75	63.7	6.20	[338]
ITO/CNT-g-PDDT:P3ThEt-g-PANI	spin coating	PBDT-DTNT:PC_61_BM	0.71	12.84	62.0	5.65	[344]
ITO/CNt:P3ThEt-g-PANI	spin coating	P3HT:PC_71_BM	0.68	12.85	60.7	5.30	[345]
*Small organic molecules*							
ITO/TTA (5.26)	spin coating	PTB7-Th:PC71BM	0.80	16.56	69.04	9.09	[377]
ITO/NiO_x_:F_4_-TCNQ (5.30)	spin coating	PTB7-Th:PC71BM	0.78	16.80	65.20	8.59	[370]
ITO/DIPO-Ph_4_/PEDOT:PSS (4.7)	vacuum deposition/spin coating	P3HT:PC61BM/	0.60	11.50	47.00	4.60	[371]
NPB/MoO_3_/Ag (5.4)	thermal evaporation	P3HT:PC61BM	0.60	10.04	63.00	3.94	[374]
MeOPhN-DBC/MoO3/Al (5.0)	vacuum deposition	P3HT:PC61BM	0.63	12.44	47.00	3.68	[376]
ITO/NiO_x_:F_4_-TCNQ (5.30)	spin coating	P3HT:PC61BM	0.59	9.89	61.60	3.59	[370]
NPB/Ag (5.4)	vacuum deposition	P3HT:PC71BM	0.57	9.49	48.90	2.63	[373]
ITO/BF-DPB:NDP9 (5.23)	spin coating	ZnPC:C60	0.51	7.50	55.00	2.10	[369]
Ag NWs/BF-DPB:NDP9 (5.23)	spin coating	ZnPC:C60	0.49	7.60	55.00	2.10	[369]
ITO/MoO3:HPCzI (5.3/5.1)	thermal evaporation	CuPC:C60	0.49	6.63	53.00	1.71	[372]
ITO/HPCzI (5.1)	thermal evaporation	CuPC:C60	0.49	6.22	53.00	1.62	[372]

## Abbreviations

Y6	(2,2′-((2Z,2Z)-((12,13-bis(2-ethylhexyl)-3,9-diundecyl-12,13-dihydro-[1,2,5]thiadiazolo[3,4-e]thieno[2″,3″:4′,50]thieno[2′,3′:4,5]pyrrolo[3,2-g]thieno[2′,3′:4,5]thieno[3,2-b]indole-2,10-diyl)bis(methanylylidene))bis(5,6-difluoro-3-oxo-2,3-dihydro-1*H*-indene-2,1-diylidene))dimalononitrile)
O-IDTBR	(5Z,5′Z)-5,5′-{[7,7′-(4,4,9,9-tetraoctyl-4,9-dihydro-s-indaceno[1,2-b:5,6-b’]dithiophene-2,7-diyl)bis(benzo[c][1,2,5]thiadiazole-7,4-diyl)]bis(methanylylidene)}bis(3-ethyl-2-thioxothiazolidin-4-one)
PC_71_BM	(6,6)-phenyl-C71-butyric acid methyl ester
HPCzI	1,3,4,5,6,7-hexaphenyl-2-{3′-(9-ethylcarbazolyl)}-isoindole
ICBA	1′,1″,4′,4″-Tetrahydro-di[1,4]methanonaphthaleno[1,2:2′,3′,56,60:2″,3″][5,6]fullerene-C60
TEMPO	2,2,6,6-tetramethylpiperidine-1-oxoammonium
IDIC	2,2′-[(4,4,9,9-tetrahexyl-4,9-dihydro-s-indaceno[1,2-b:5,6-b′]dithiophene-2,7-diyl)bis[methylidyne(3-oxo-1*H*-indene-2,1(3*H*)-diylidene)]]bis-propanedinitrile
DIPO-Ph_4_	2,2′,6,6′-tetraphenyl-dipyranylidene
F4-TCNQ	2,3,5,6-tetrafluoro-7,7,8,8-tetracyanoquinodimethane
EGME	2-methoxyethanol
PTCDA	3,4,9,10-perylenetetracarboxylic dianhydride
IT-4F	3,9-bis(2-methylene-((3-(1,1-dicyanomethylene)-6,7-difluoro)-indanone))-5,5,11,11-tetrakis(4-hexylphenyl)-dithieno[2,3-d:2′,3′-d′]-s-indaceno[1,2-b:5,6-b’]dithiophene
ITIC	3,9-bis(2-methylene-(3-(1,1-dicyanomethylene)-indanone))-5,5,11,11-tetrakis(4-hexylphenyl)-dithieno-[2,3-d:2′,3′-d′]-s-indaceno[1,2-b:5,6-b′]dithiophene
ITIC-Th	3,9-bis(2-methylene-(3-(1,1-dicyanomethylene)-indanone))-5,5,11,11-tetrakis(5-hexylthienyl)-dithieno-[2,3-d:2′,3′-d′]-s-indaceno[1,2-b:5,6-b′]dithiophene
CBP	4,4′-*N*,*N*′-dicarbazole-biphenyl
a-MWNTs	Amino-functionalized multi-walled carbon nanotubes
AHM	Ammonium heptamolybdate
AIL	Anode interfacial layer
AFM	Atomic force microscope
BPQDs	Black phosphorous quantum dots
bf-MWCNTs	Boronic acid functionalized multi-walled carbon nanotubes
BHJ	Bulk heterojunction
CNT	Carbon nanotubes
CTAB	Cetyltrimethylammonium bromide
CoO_x_	Cobalt oxide
CPEs	Conjugated polyelectrolytes
CIGS	Copper indium gallium diselenide
CuO_x_	Copper oxide
CuS_x_	Copper sulfide
MeOPhN-DBC	Dibenzo[g,p]chrysene derivative, 3,6,11,14-tetramethoxyphenylamine- dibenzo[g,p]chrysene
DMSO	Dimethyl sulfoxide
DMF	Dimethylformamide
DC	Direct current
DA	Dopamine
ETL	Electron transport layer
EQE	External quantum efficiency
FF	Fill factor
GSL	Grafted sulfonated-acetone-formaldehyde lignin
GO	Graphene oxide
GQDs	Graphene quantum dots
g-C_3_N_4_	Graphitic carbon nitrile
HOMO	High occupied molecular orbital
HTL	Hole transport layer
H_y_MoO_3-x_	Hydrogenated molybdenum oxide
ITO	Indium tin oxide
P_in_	Input power
IPA	Isopropanol
LIL	Laser interference lithography
LSPR	Localized surface plasmon resonance
LUMO	Lowest unoccupied molecular orbital
P_max_	Maximum power output
MO	Metal oxides
MW	Microwave
NPB	*N*,*N*′-bis(1-naphthalenyl)*N*,*N*′-bis-phenyl-(1,1′-biphenyl)-4,4′-diamine
BF-DPB	*N*,*N*’-((diphenyl-*N*,*N*’-bis)9,9,-dimethyl-fluoren-2-yl)-benzidine
NDs	Nanodots
NPs	Nanoparticles
NRs	Nanorods
NWs	Nanowires
NFD	Nickel formate dihydrate
NiO_x_	Nickel oxide
NiS_x_	Nickel sulfide
nGQDs	Nitrogen doped graphene quantum dots
V_oc_	Open circuit voltage
OLEDs	Organic light-emiting diodes
OSCs	Organic solar cells
P_out_	Output power
PCBM	Phenyl-C61-butyric acid methyl ester
PMA	Phosphomolybdic acid
PL	Photoluminescence
PV	Photovoltaic
PC	Phthalocyanine
PTB7	Poly [[4,8-bis[(2-ethylhexyl)oxy]benzo[1,2-b:4,5-b′]dithiophene-2,6-diyl][3-fluoro-2-[(2-ethylhexyl)carbonyl]thieno[3,4-b]thiophenediyl]]
PCPDT	Poly 3,4-dithia-7*H*-cyclopenta[a]pentalene
FBT-TH4	Poly 5,6-difluorobenzothiadiazole
PDMT	Poly(3,4-dimethoxythiophene)
PEDOT	Poly(3,4-ethylenedioxythiophene)
PEDOT:PSS	Poly(3,4-ethylenedioxythiophene)-poly(styrene sulfonate)
P3HT	Poly(3-hexylthiophene)
P3HT_50_-b-PSS_23_	Poly(3-hexylthiophene)-*b*-poly(p-styrenesulfonate)
PEDOT-S	Poly(4-(2,3-dihydrothieno[3,4-b][1,4]dioxin-2-yl-methoxy)-1-butanesulfonic acid)
PTPCz	Poly(carbazolyl triphenylethylene) derivative
PDMS	Poly(dimethylsiloxane)
PMMA	Poly(methylmethacrylate)
PSS	Poly(styrenesulfonate)
PM6	Poly[(2,6-(4,8-bis(5-(2-ethylhexyl)-4-fluorothiophen-2-yl)benzo[1,2-b:4,5-b′]dithiophene))-co-(1,3-di(5-thiophene-2-yl)-5,7-bis(2-ethylhexyl)-benzo[1,2-c:4,5-c′]dithiophene-4,8-dione))]
PBDB-T	Poly[(2,6-(4,8-bis(5-(2-ethylhexyl)thiophen-2-yl)benzo[1,2-b:4,5-b′]dithiophene)-co-(1,3-di(5-thiophene-2-yl)-5,7-bis(2-ethylhexyl)benzo[1,2-c:4,5-c′]dithiophene-4,8-dione)]
PffBT4T-2OD	Poly[(5,6-difluoro-2,1,3-benzothiadiazol-4,7-diyl)-alt-(3,3‴-di(2-octyldodecyl)-2,2′;5′,2″;5″,2‴-quaterthiophen-5,5‴-diyl)]
PFN	Poly[(9,9bis(3′-(*N*,*N*-dimethylamino)propyl)-2,7-fluorene)-alt-2,7-(9,9dioctylfluorene)]
PSFP-DTBTP	Poly[(9,9-bis(4-sulfonatobutyl sodium) fluorene-alt-phenylene)-ran-(4,7-di-2-thienyl-2,1,3-benzothiadiazole-alt-phenylene)]
TFB	Poly[(9,9-dioctylfluorenyl-2,7-diyl)-alt-(4,4′-(*N*-(4-butylphenyl)))]
PNDIT-F3N	Poly[[2,7-bis(2-ethylhexyl)-1,2,3,6,7,8-hexahydro-1,3,6,8-tetraoxobenzo[lmn] [3,8]phenanthroline-4,9-diyl]-2,5-thiophenediyl[9,9-bis[3′((*N*,*N*-dimethyl)-*N*-ethylammonium)]propyl]-9*H*-fluorene-2,7-diyl]-2,5-thiophenediyl]
PhNa-1T	Poly[1,4-bis(4-sulfonatobutoxy)benzene-thiophene]
PCP-Na	Poly[2,6-(4,4-bis-(propane-1-sulfonate sodium)-4*H*-cyclopenta[2,1-b;3,4-b′ ]dithiophene)-alt-(4,4′-biphenyl)]
P3HTN	Poly[3-(6′-*N*,*N*,*N*-trimethyl ammonium)-hexylthiophene] bromide
PTB7-Th	Poly[4,8-bis(5-(2-ethylhexyl)thiophen-2-yl)benzo[1,2-b;4,5-b′]dithiophene-2,6-diyl-alt-(4-(2-ethylhexyl)-3-fluorothieno[3,4-b]thiophene-)-2-carboxylate-2-6-diyl)]
PBDT-TS1	Poly[4,8-bis(5-(octylthio)thiophen-2-yl)benzo[1,2-b;4,5-b′]dithiophene-2,6-diyl–alt–(4-(2- ethylhexyl)-3-fluorothieno[3,4-b]thiophene-)-2-carboxylate-2-6-diyl]
PFtT-D	Poly[9,9-bis(4′-sulfonatobutyl)fluorene-alt-thieno[3,2-b]thiophene]
PFSe	Poly[9,9-bis(4′-sulfonatobutyl)fluorene-alt-selenophene]
PFT-D	Poly[9,9-bis(4′-sulfonatobutyl)fluorene-*alt*-thiophene]
PBDT-DTNT	Poly[benzodithiophene-bis(decyltetradecyl-thien)naphthothiadiazole]
PCDTBT	Poly[*N*-9′-heptadecanyl-2,7-carbazole-alt-5,5(4′,7′-di-2-thienyl-2′,1′,3′-benzothiadiazole)]
PANI	Polyaniline
PEG	Polyethylene glycol
PSC	Polymer solar cell
PMC	Polynuclear metal-oxo clusters
p-TPCF	Polytriphenylcarbazole fluoranthene
PCE	Power conversion efficiency
QDs	Quantum dots
rrP3HT	Regioregular poly(3-hexylthiophene-2,5-diyl)
RMS	Root meand square
SEM	Scanning electron microscopy
R_s_	Series resistance
J_sc_	Short circuit current
R_sh_	Shunt resistance
SWCNTs	Single-walled carbon nanotubes
SILAR	Successive ionic layer adsorption and reaction
F_4_TCNQ	Tetrafluorotetracyanoquino-dimethane
TTA	Tetrathiafulvalene
TTF-py	Tetrathiafulvalene pyridine derivative
P3HT-Si	Triethoxysilane terminated poly(3-hexylthiophene)
WO_x_	Tungsten oxide
WS_x_	Tungsten sulfide
UVO	Ultraviolet ozone
uSWNT	Unzipped single-walled carbon nanotubes
VO_x_	Vanadium oxide
VoPC	Vanadylphthalocyanine
WF	Work function
